# Oxygen-18 Labeling Defines a Ferric Peroxide (Compound 0) Mechanism in the Oxidative Deformylation of Aldehydes by Cytochrome P450 2B4

**DOI:** 10.1021/acscatal.4c00106

**Published:** 2024-01-31

**Authors:** Yasuhiro Tateishi, Kevin D. McCarty, Martha V. Martin, F. Peter Guengerich

**Affiliations:** Department of Biochemistry, Vanderbilt University School of Medicine, Nashville, Tennessee 37232-0146, United States

**Keywords:** cytochrome P450, C−C bond cleavage, aldehydes, oxygen-18 labeling, compound 0, compound I

## Abstract

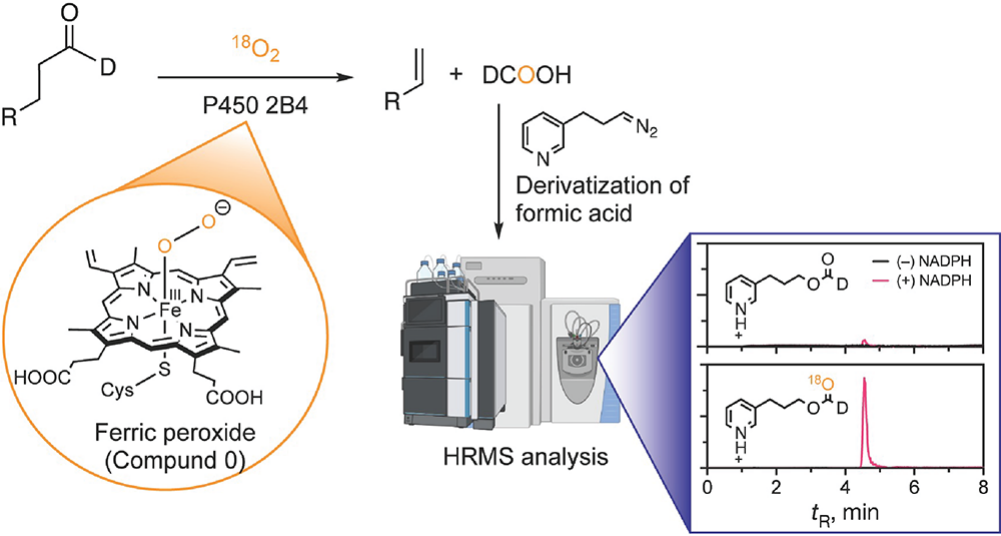

Most cytochrome P450 (P450) oxidations are considered to occur with the active oxidant being a perferryl oxygen (FeO^3+^, Compound I). However, a ferric peroxide (FeO_2_^®^, Compound 0) mechanism has been proposed, as well, particularly for aldehyde substrates. We investigated three of these systems, the oxidative deformylation of the model substrates citronellal, 2-phenylpropionaldehyde, and 2-methyl-2-phenylpropionaldehyde by rabbit P450 2B4, using ^18^O labeling. The formic acid product contained one ^18^O derived from ^18^O_2_, which is indicative of a dominant Compound 0 mechanism. The formic acid also contained only one ^18^O derived from H_2_^18^O, which ruled out a Compound I mechanism. The possibility of a Baeyer–Villiger reaction was examined by using synthesized possible intermediates, but our data do not support its presence. Overall, these findings unambiguously demonstrate the role of the Compound 0 pathway in these aldehyde oxidative deformylation reactions.

Cytochrome P450 (P450, CYP) reactions dominate the oxidation of chemicals, both natural and anthropomorphic.^1,2^ The role of a perferryl oxygen (FeO^3+^, Compound I, Scheme 1) has been established^3,4^ and it is generally considered to be the active oxidant in most, if not all, P450 oxidations.^5,6^ However, other oxidizing species have been proposed, including the ferric peroxide (FeO_2_^–^, Compound 0), which precedes Compound I in the catalytic cycle (Scheme 1) (the nomenclature of Compound 0 usually refers to the ferric hydroperoxo species, but considering that these two are protonated/unprotonated forms with each other and are focused on its reactivity, we denote the ferric peroxide anion as Compound 0). Approaches to discrimination between Compound 0 and Compound I reactions have involved computation,^7,8^ biomimetic model reactions,^9,10^ solvent isotope effects,^11,12^ site-directed mutagenesis,^13−15^ use of oxygen surrogates with P450s,^16−18^ and substrate analogues.^1,19−21^ Perhaps the most definitive approach is an analysis of isotopic oxygen labeling, first applied by Akhtar’s group in 1976.^22,23^ The approach is powerful when it can be applied but is technically challenging regarding the detection of small carboxylic acids as reaction side products.

**Scheme 1:**
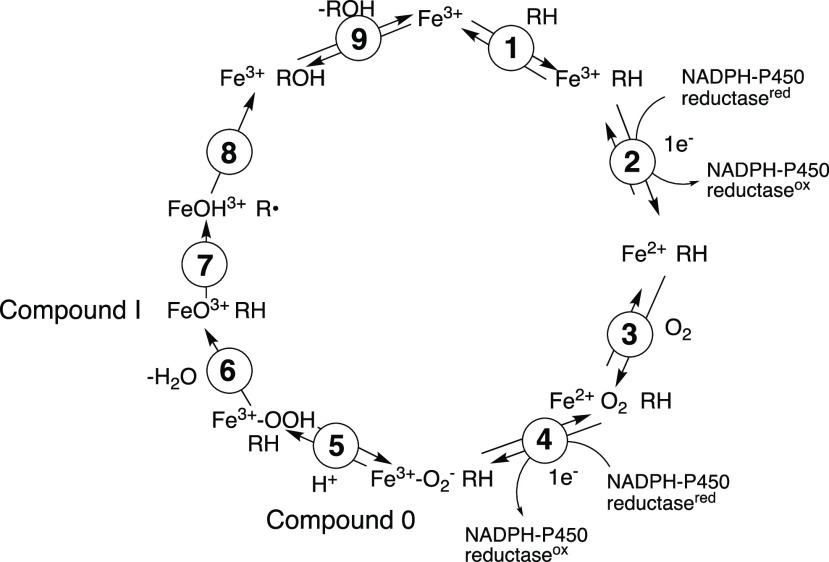
General P450 Catalytic Mechanism

In our previous work, we have employed this latter approach—conducting enzyme reactions in an ^18^O_2_ atmosphere (^18^O experiment)—to study the C–C bond cleavage reactions catalyzed by the steroidogenic P450 19A1,^24^ 11A1,^25^ 17A1,^26^ and 51^27^ enzymes. One of the keys to this work was the development of pyridine-based derivatizing reagents to allow analysis of small carboxylic acid products (e.g., formic acid) with electrospray mass spectrometry (MS) by detecting them as esters with high sensitivity due to the basic pyridine moiety. Incorporation of deuterium into substrates at the position of cleavage allows enzymatically produced acids to be distinguished from ubiquitous endogenous acids.^23^

One classic reaction regarding a proposed role of Compound 0 is the oxidative deformylation of aldehydes catalyzed by rabbit P450 2B4, a xenobiotic-metabolizing P450, producing olefins.^28−30^ The conclusion about a role for Compound 0 was based largely on the results of the use of oxygen surrogates (H_2_O_2_ and iodosylbenzene)^29^ and a P450 2B4 mutant,^13^ but neither of these approaches are unambiguous and, to the best of our knowledge, no ^18^O labeling results have been reported. We have now applied the ^18^O methodology to study the mechanism of the P450 2B4-catalyzed oxidative carbon–carbon cleavage reaction of aldehydes.

The natural terpene citronellal is one of the P450 2B4 substrates and yields 2,7-dimethyl-1,5-heptene and formic acid as products.^28^ Two other model aldehydes, 2-phenylpropionaldehyde and 2-methyl-2-phenylpropionaldehyde, are also substrates and have been shown to be mechanism-based inhibitors of P450 2B4.^31^ In order to avoid the problem of the contribution of the endogenous formic acid, we prepared each aldehyde with a single deuterium atom (citronellal-*d*, **1**; 2-phenylpropionaldehyde-*d*, **2**; and 2-methyl-2-phenylpropionaldehyde-*d*, **3**), which is nonexchangeable (Scheme 2A). The Compound 0, Compound 0/Baeyer–Villiger, and Compound I mechanisms and the expected labeling patterns are shown in Scheme 2B–E using citronellal-*d* (**1**) as an example. The Compound 0 mechanism begins with the nucleophilic attack of ferric peroxide, which results in oxygen incorporation from the heme iron species into the formic acid product. Although it has not been reported in the case of P450 2B4, a Baeyer–Villiger-type reaction yielding a one-carbon homologated ester (**4** in Scheme 2C) might be possible when Compound 0 is acting as a reactive iron species. Indeed, some P450s have been proposed to involve a Baeyer–Villiger mechanism.^27,32^ In a Compound I mechanism, one hydrogen atom of a *gem*-diol is abstracted (but not from the alcohol). Electron transfer yields the carbocation, which then rearranges to release formic acid and form an olefin product.^24,33^ On the basis of these proposed mechanisms, Compound I vs Compound 0 reactions can be distinguished by identifying the source of oxygen incorporated into the formic acid product.

**Scheme 2:**
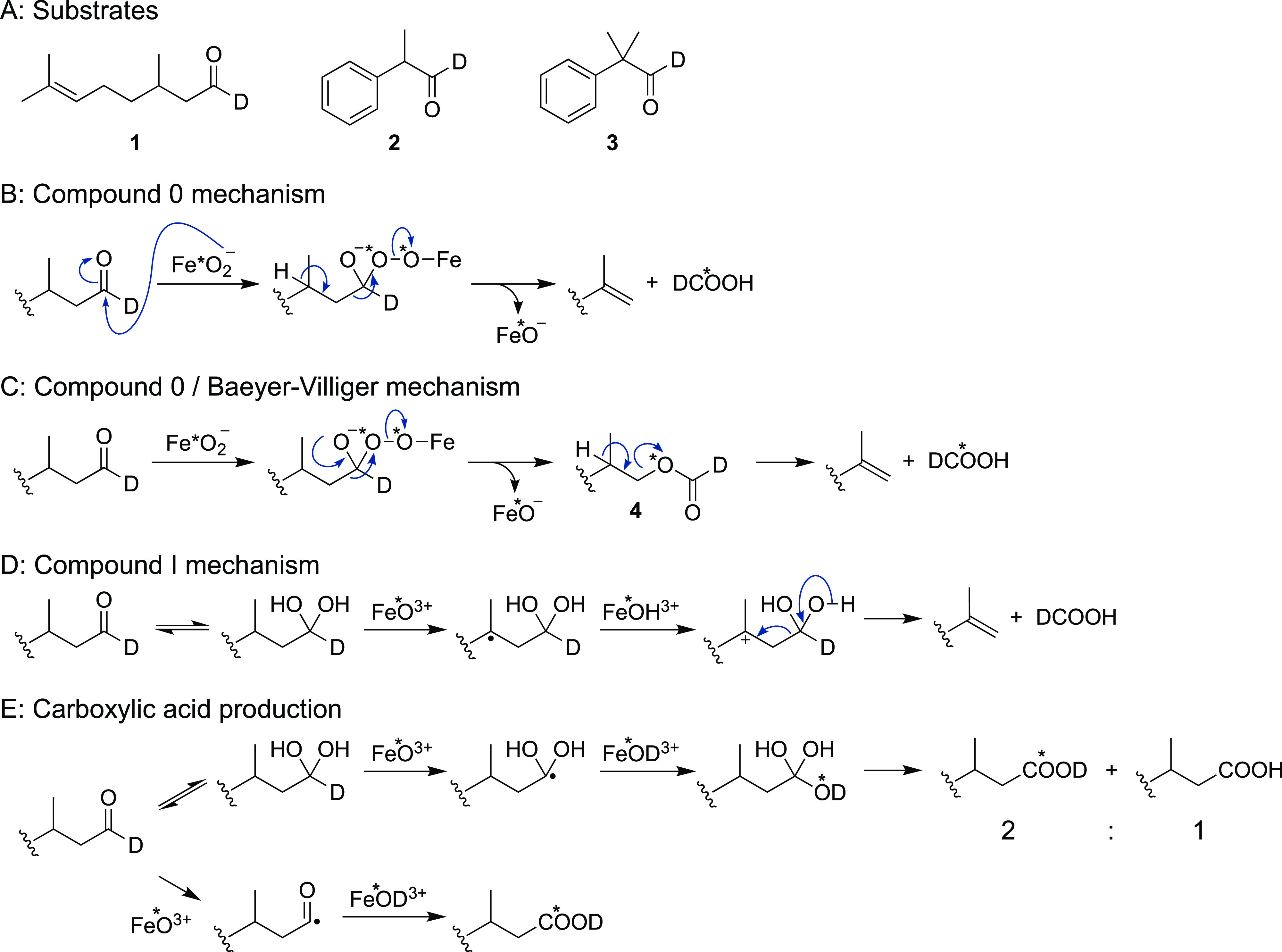
Substrates Used in This Study (A) and Possible Mechanisms for Oxidation of Citronellal-*d* (**1**) (B–E)

Rates of P450 2B4-catalyzed deformylations are relatively slow (0.1–1 min^–1^), which is problematic in obtaining an adequate product for the analysis. To improve the sensitivity, we revised the procedures for extraction and derivatization of formic acid using the diazo reagent 1-diazo-3-(3-pyridinyl)propane (**5**)^24,27^ as detailed in the Supporting Information. Additionally, a P450 17A1-catalyzed 17α-hydroxylation of progesterone reaction was included in all of the samples as an internal control to accurately calculate the percentage of ^18^O incorporation in each sample (i.e., from ^18^O analysis of the product 17α–OH progesterone), thereby correcting for any artifactual loss of ^18^O_2_ (e.g., gas leaks).

Derivatization of formic acid produced in the ^18^O experiment of deuterium-labeled aldehydes will yield 3-(pyridin-3-yl)propyl formate-*d* that either lacks (**6a**) or contains (**6b**) one ^18^O atom (Schemes 2 and 3). The ^18^O experiment with **1** predominantly produced **6b** (with one ^18^O atom) and a very small amount of **6a** (with no ^18^O), which supports a Compound 0 mechanism (Table 1; Figures 1 and 2). The internal control reaction with P450 17A1 showed ∼95% incorporation of ^18^O in these reactions. When the reaction was done in H_2_^18^O (under ^16^O_2_) using an ^18^O-labeled substrate (^18^O-citronellal-*d,***1′**), the formic acid contained a single ^18^O (detected as **6b**) and no doubly labeled formic acid was detected (as the pyridyl ester **6c**) (Figure S4), which is in further support of the Compound 0 mechanism. The carboxylic acid oxidation product (2,8-dimethyloctanoic acid or citronellic acid, Scheme 2D) was also derivatized with **5** to yield ester **7a** (with no ^18^O) and **7b** (with one ^18^O atom), and nearly a single equivalent of ^18^O incorporation was observed (Scheme 3; Figures 1 and 2). If the Compound I mechanism shown (Scheme 2E) were involved, the abstraction of a hydrogen atom from the *gem*-diol and oxygen rebound would create a transient *gem*-triol that would dehydrate to give the carboxylic acid, and only 1/3 of one ^18^O atom would be present in the acid. However, we cannot rule out the possibility that direct hydrogen atom abstraction from the aldehyde occurred with Compound I followed by oxygen rebound to generate the carboxylic acid.

**Figure 1:**
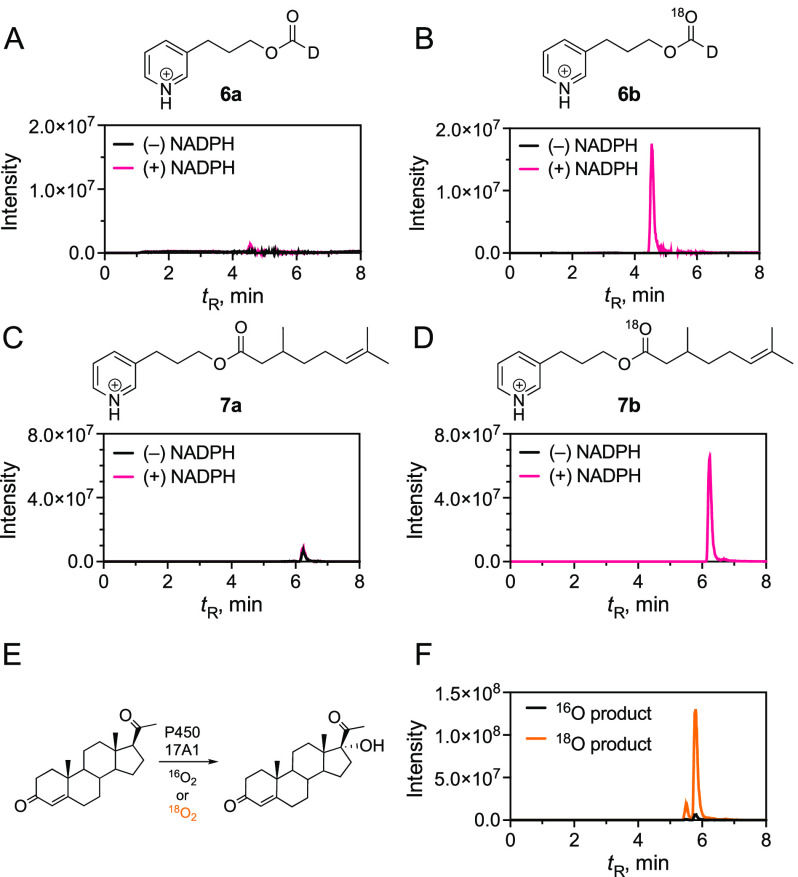
P450 2B4 incubation with citronellal-*d* (**1**) under ^18^O_2_ atmosphere. (A–D) Representative mass traces of **6a** (*m*/*z* 167.0925) (A), **6b** (*m*/*z* 169.0968) (B), **7a** (*m*/*z* 290.2115) (C), and **7b** (*m*/*z* 292.2157) (D) with 5 ppm mass tolerance. The retention times (4.5 min for **6a** and **6b**, 6.2 min for **7a** and **7b**) matched with the synthesized standards. Each trace shows peaks with (**−**, red line) or without (**−**, black line) the nicotinamide adenine dinucleotide phosphate (NADPH)-generating system during incubation. (E) Scheme for 17α-hydroxylation of progesterone by P450 17A1, which was included in each Thunberg tube to normalize the percentage of the ^18^O incorporation. (F) Representative mass trace of *m*/*z* 331.2268 (**−**, black line, 17α-^16^OH progesterone) and 333.2310 (**−**, orange line, 17α-^18^OH progesterone) with 5 ppm mass tolerance. The calculated percentage of ^18^O incorporation from the peak areas was 95%.

**Figure 2:**
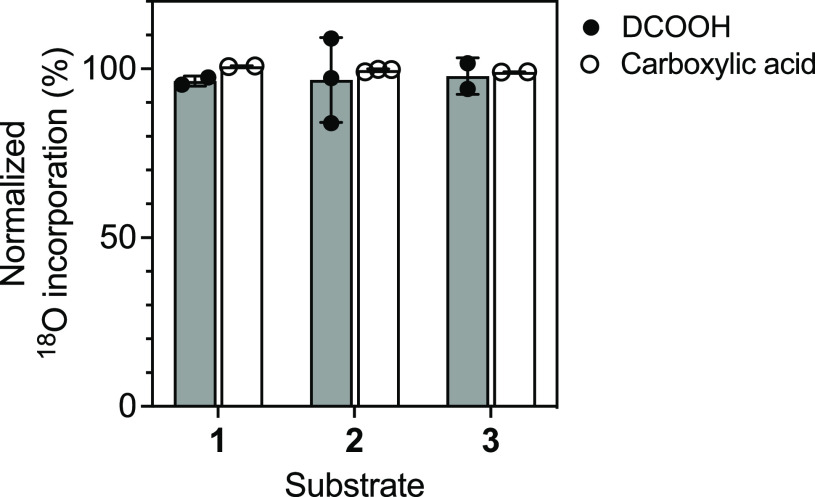
Summary of ^18^O incorporation from ^18^O_2_ into formic acid and carboxylic acids. All values were normalized by the percentage of ^18^O incorporation (calculated with progesterone 17α-hydroxylation by P450 17A1). The results indicate means ± range of duplicate (for **1** and **3**) or SD of triplicate (for **2**) assays.

**Scheme 3:**
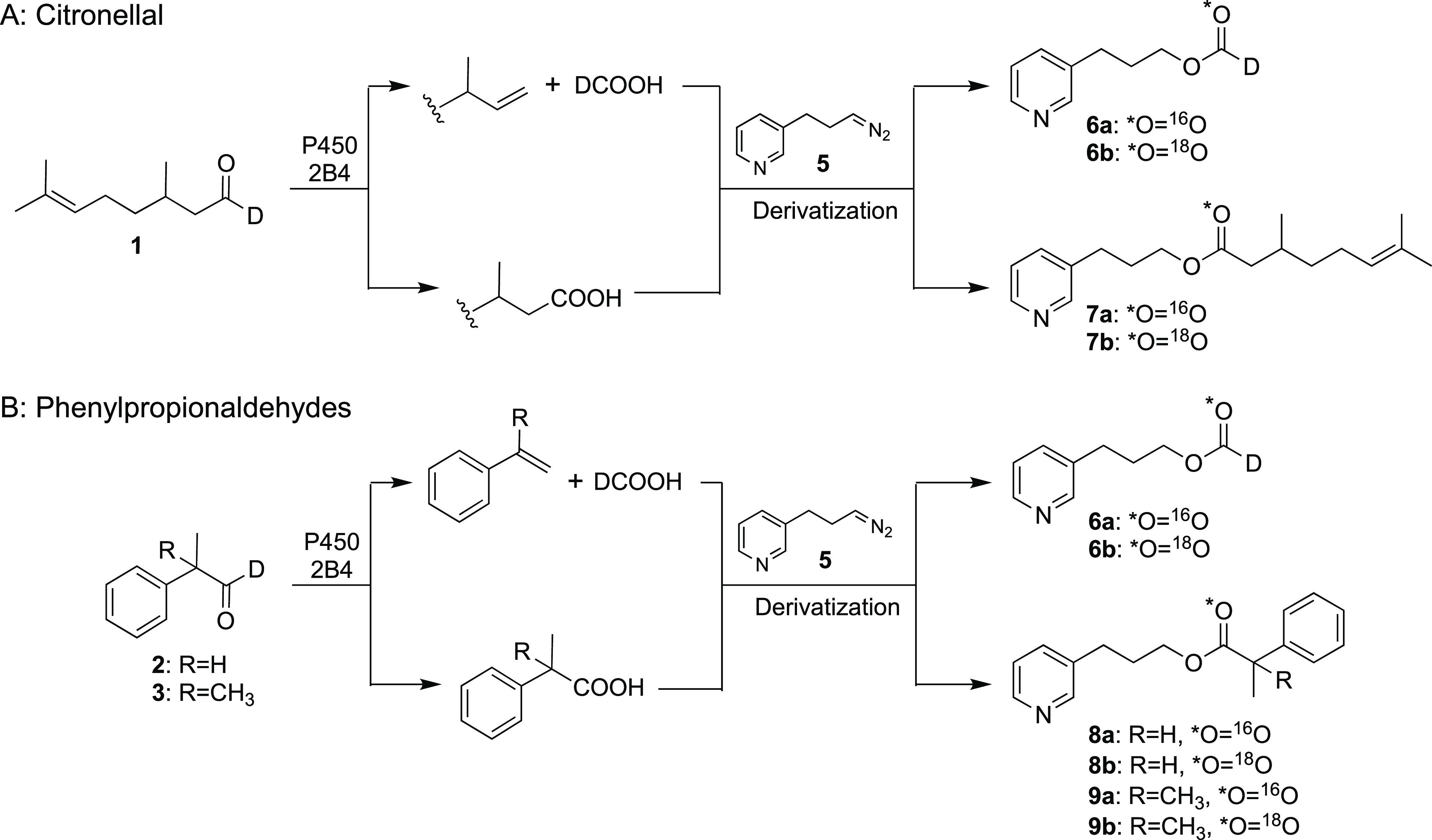
Derivatization of Formic Acid-*d* and Carboxylic Acid Products by Diazo Reagent **5**

**Table 1:** ^18^O Incorporation Results

substrate	^18^O incorporation (17α–OH progesterone) (%)	formic acid-*d* (% ^18^O)	carboxylic acid (% ^18^O)
**1**^a^	95 ± 1	91 ± 1	96 ± 1
**2**^b^	96 ± 1	93 ± 12^c^	95 ± 1
**3**^a^	98 ± 1	96 ± 4^c^	97 ± 1

a The results are presented as mean ± range of duplicate assays.

b The results are presented as mean ± SD of triplicate assays.

c Peak areas of **6a** (^16^O product) detected from minus NADPH samples were subtracted prior to calculations.

Others have attempted to use solvent kinetic isotope effects (KIEs) to distinguish Compound 0 and Compound I mechanisms, but mixed conclusions have been reported.^11,12,26,34^ Jencks, Kresge, and others have criticized the approach in that a myriad of protons are exchanged with deuterium in the protein without knowledge of changes in bonding or structure.^35−39^ We determined solvent KIEs for the production of both formic acid and 2,8-dimethyloctanoic acid, but no significant effects were observed (Figure S5) even though the evidence clearly defines a Compound 0 mechanism.

We also investigated two other model aldehydes, **2** and **3**. These two compounds were both substrates and yielded formic acid plus styrene and α-methylstyrene as products, as well as 1-phenylethanol and 2-phenyl-2-propanol (Scheme 4). For unknown reasons, these compounds were prone to release some formic acid-*d* nonenzymatically (∼0.02% of the substrate), thereby making the interpretation of ^18^O experiments more complicated. However, after subtracting the peak area of **6a** that was detected in the minus nicotinamide adenine dinucleotide phosphate (NADPH) control assays, the extent of incorporation of ^18^O from ^18^O_2_ was >90%, which is also indicative of a Compound 0 mechanism for both (Table 1; Figures 2, S6, and S7). Carboxylic acid products were also detected as pyridyl esters **8** and **9**, predominantly with one ^18^O atom (**8b** and **9b**), as observed in the case of **1** (Table 1; Scheme 3; Figures 2, S6, and S7).

**Scheme 4:**
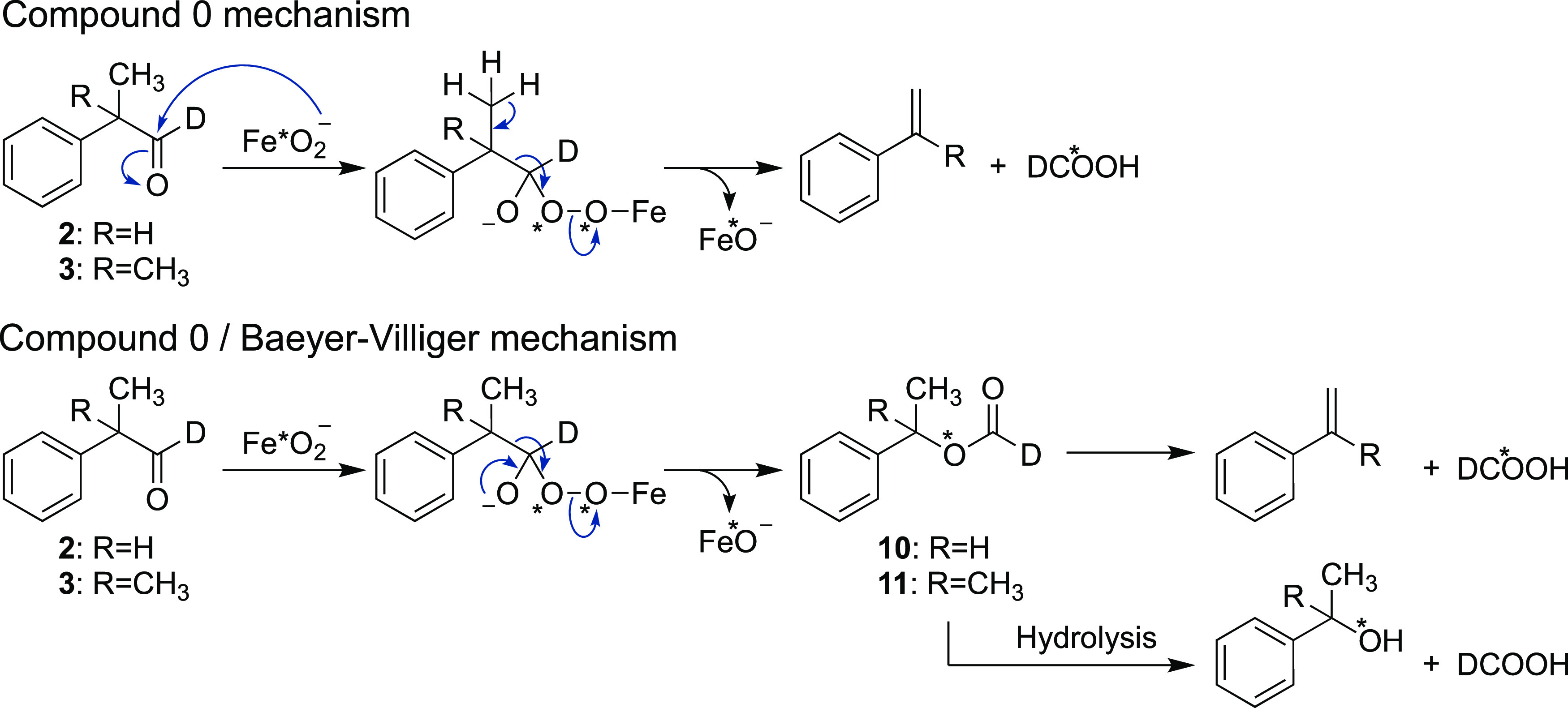
Proposed Mechanism of Oxidation of Deuterated Phenylpropionaldehydes (**2, 3**)

As mentioned, the possibility existed that the Compound 0 mechanism could include a Baeyer–Villiger rearrangement prior to C–C scission.^27,32^ To address this mechanistic question, we synthesized the proposed Baeyer–Villiger intermediates of each of the three substrates (**4**, **10**, and **11**). Because the putative intermediate expected from citronellal (**4**) displayed poor ionization character and minimal UV activity, our analysis was based on experiments with only the phenylpropionaldehyde model substrates. LC-UV analyses of the P450 2B4 reaction products did not provide any evidence of the formation of **10** or **11** (Figure S8). In addition, incubation of **10** with P450 2B4 did not yield the olefin product (styrene) but did produce a trace amount of alcohol product (1-phenylethanol), although the latter product was also formed in the absence of P450, which suggests that Baeyer–Villiger-type chemistry is not utilized in the deformylation of small aldehydes by P450 2B4.

Unlike steroidogenic P450 enzymes (e.g., P450 11A1, 17A1, and 19A1), a xenobiotic-metabolizing P450 2B4 can catalyze a wide variety of reactions at multiple carbons. Indeed, when substrates were incubated under an ^18^O_2_ atmosphere, mono-oxygenated metabolites of **7b**, **8b**, and **9b** were also detected (Figure S9), thereby indicating that the carboxylic acid products were further oxidized by P450 2B4 or vice versa. The possible oxidized positions were investigated using **2** as a model substrate (Figure S10), which provided evidence that the phenyl group rather than the β carbon would be a favorable oxidized position. These data further support our conclusion that the C–C bond cleavage reaction is catalyzed by Compound 0, not Compound I, in that the hydrogen at β-carbon is not abstracted by Compound I during oxidation, at least in the case of **2**.

One interesting question is what determines the fate of Compound 0, i.e., what makes Compound 0 react with the carbonyl group (which leads to C–C bond cleavage) instead of a proton (which yields Compound I). To get some insights into that question, we have compared the rates of deformylation and carboxylic acid formation (Figure S11). A decreasing ratio of deformylation vs carboxylic acid production was observed by adding more methyl groups at α-carbon, which is consistent with what was observed by the Coon group.^31^ The results indicate that the nucleophilic attack by ferric peroxide (Compound 0) is less favorable in an environment with more steric hindrance; thus, the heme ferric peroxide anion would be more likely to be protonated, thereby yielding Compound I and producing carboxylic acid.

With P450 51 enzymes, we have recently found that five of these use a mixture of Compound 0 and Compound I mechanisms in the oxidative deformylation of 14α-formyl(24,25-dihydro)lanosterol.^27^ These P450s varied from 51 to 88% use of the Compound 0 mechanism, but with P450 2B4, nearly quantitative use of the Compound 0 mechanism was observed (Figures 1 and 2), and no detectable Compound I reactions were observed (i.e., H_2_^18^O experiments, Figure S6). We attribute the differences in utilization of Compound 0 and Compound I mechanisms (Scheme 1) to the relative rates for the ferric peroxy anion to be protonated versus nucleophilic attack on an electrophilic carbonyl group, but the roles of individual amino acid residues are only a matter of speculation. In the case of human and rat P450 51A1, there is evidence for a Baeyer–Villiger rearrangement (Scheme 2C),^27,32^ but this product was not observed here for these P450 2B4 reactions. We are also unable to define the elements of the P450s that control the Baeyer–Villiger rearrangement at this time.

The ^18^O labeling approach that we have employed here is applicable to other P450-catalyzed oxidative deformylations, e.g., P450 19A1-catalyzed androstenedione aromatization^24^ (vide supra) and P450 51-catalyzed lanosterol 14α-demethylation.^27,40^ Although Ortiz de Montellano and co-workers employed ^18^O_2_ to study an oxidative terminal side chain carbon cleavage reaction of cholesterol by bacterial P450 125A1,^41^ direct evidence of Compound 0 contribution was not presented, and this question could also be addressed by employing deuterium-labeled formic acid analysis. In principle, the approach can be applied to C–C bond scissions that yield acetic acid (e.g., P450 17A1-catalyzed 17α,20-lyase reaction and P450 1A2-catalyzed side-chain cleavage of nabumetone),^26,42^ but with α-ketols, the ^18^O labeling results are not unambiguous.^20,43,45^

In conclusion, we provide oxygen-18 labeling evidence for a Compound 0 pathway in the deformylation of the classical substrate citronellal^28^ and two other phenylpropionaldehyde model substrates by P450 2B4.^31^ This study defines P450 reactions with aldehydes and ketones that undergo C–C bond scission, although there is still ambiguity about the mechanisms for some other P450s and reactions.^5,20,45^

## Supporting Information Available

Synthetic procedures, enzyme assays, reagents, expression and purification of P450 2B4 and other enzymes, LC-MS analyses, preparation of diazo reagent, enzyme incubations under an ^18^O_2_ atmosphere and in H_2_^18^O, kinetic solvent isotope effects, search for a Baeyer–Villiger intermediate and additional metabolites, comparison of deformylation and carboxylic acid formation, and NMR spectra (PDF)
