# Supplementary material for: Oxygen-18 Labeling Defines a Ferric Peroxide (Compound 0) Mechanism in the Oxidative Deformylation of Aldehydes by Cytochrome P450 2B4
Source: ACS Catal. 2024 Jan 31;14(4):2388–94. doi: 10.1021/acscatal.4c00106 (PMC10877606; doi:10.1021/acscatal.4c00106)
Supplement: Supplementary file 1 — cs4c00106_si_001.pdf [file cs4c00106_si_001.pdf]

SUPPORTING INFORMATION

Oxygen-18 Labeling Defines a Ferric Peroxide (Compound 0) Mechanism in the Oxidative Deformylation of Aldehydes by Cytochrome P450 2B4

Yasuhiro Tateishi, Kevin D. McCarty, Martha V. Martin, and F. Peter Guengerich*

Department of Biochemistry, Vanderbilt University School of Medicine, Nashville, Tennessee 37232-0146, United States

TABLE OF CONTENTS

EXPERIMENTAL PROCEDURES

1. Synthetic procedures S-4

Reagents S-4

General Information S-4

Synthesis of 3,7-dimethyloct-6-enal-1-*d* (citronellal-*d*, **1**) S-5

Synthesis of phenyl aldehydes (**2**, **3**) S-7

Synthesis of 3-(pyridine-3-yl)propyl esters (**6a**, **7a**, **8a**, **9a**) S-10

Synthesis of possible Baeyer-Villiger intermediates (**4**, **10**, **11**) S-12

Synthesis of precursor (nitrosourea) of diazo reagent (**5**) S-14

Synthesis of 3-(pyridine-3-yl)propyl esters of potential products of **8a** (**M1** and **M2**) S-16

2. Enzyme assays S-18

Instruments S-18

Reagents S-18

Expression of P450 2B4 S-19

Purification of P450 2B4 S-21

Preparation of other enzymes S-22

LC-MS analyses S-22

Preparation of diazo reagent (**5**) S-23

Enzyme incubations under an $^{18}O_2$ atmosphere S-23

Enzyme incubations in $H_2{}^{18}O$ S-25

Kinetic solvent isotope effects S-26

Enzyme incubations of **2** and **3** under air S-26

Incubation of **10** with P450 2B4 S-27

SUPPLEMENTAL TABLES AND FIGURES

Table S1. List of identified peptides from proteomic analysis of purified P450 2B4 S-28

Figure S1. P450 2B4 cDNA nucleotide sequence and predicted amino acid sequence S-29

Figure S2. Reduced-CO vs. reduced difference spectrum and SDS-gel electrophoresis of purified P450 2B4 S-30

Figure S3. Mass spectra of 3-(pyridin-3-yl)propyl esters of $H^{13}COOH$ and DCOOH S-31

Figure S4. P450 2B4 incubation with **1** in $H_2{}^{18}O$ S-32

Figure S5. Kinetic solvent isotope effects S-33

Figure S6. P450 2B4 incubation with **2** under an $^{18}O_2$ atmosphere S-34

Figure S7. P450 2B4 incubation with **3** under an $^{18}O_2$ atmosphere S-35

Figure S8. Search for a Baeyer-Villiger intermediate S-36

Figure S9. Search for additional metabolites from $^{18}O_2$ experiment data S-37

Figure S10. LC-MS analysis of other metabolites of **2** S-38

Figure S11. Comparison of deformylation vs carboxylic acid formation S-39

NMR Spectra

^{1}H and ^{13}C NMR spectra of deuterated aldehydes

Citronellal-*d* (**1**) S-40, S-41

2-Phenylpropionaldehyde-*d* (**2**) S-42, S-43

2-Methyl-2-phenylpropionaldehyde-*d* (**3**) S-44, S-45

^{1}H and ^{13}C NMR spectra of 3-(pyridin-3-yl)propyl esters

3-(Pyridin-3-yl)propyl formate-*d* (**6a**) S-46, S-47

3-(Pyridin-3-yl)propyl 3,7-dimethyloct-6-enoate (**7a**) S-48, S-49

3-(Pyridin-3-yl)propyl 2-phenylpropanoate (**8a**) S-50, S-51

3-(Pyridin-3-yl)propyl 2-methyl-2-phenylpropanoate (**9a**) S-52, S-53

^{1}H and ^{13}C NMR spectra of possible Baeyer-Villiger intermediates (synthetic)

6-Methylhept-5-en-2-yl formate-*d* (**4**) S-54, S-55

1-Phenylethyl formate-*d* (**10**) S-56, S-57

2-Phenylpropyl formate-*d* (**11**) S-58, S-59

^{1}H NMR spectrum of nitrosourea S-60

References S-61

Experimental Procedures

1. Synthetic procedures

Reagents

Most chemicals were obtained from Millipore-Sigma-Aldrich (St. Louis, MO) or ThermoFisher Scientific (Waltham, MA) unless otherwise noted. 2-Phenylisobutilic acid and trimethylsilyl diazomethane were purchased from Tokyo Chemical Industries (Tokyo, Japan). Lithium aluminum deuteride ($LiAlD_4$, 98 atom % D) was purchased from Oakwood Chemical (Estill, SC).

General Information

NMR spectra were recorded on Bruker AV-400 or AV-II-600 instruments in the Vanderbilt University Small Molecule NMR Facility Core, operating at 400.13 or 600.13 MHz. $CDCl_3$ (with 0.03% tetramethylsilane, v/v) and CD_3CN were purchased from Cambridge Isotope Laboratories (Andover, MA). Tetramethylsilane (TMS, δ 0.00 ppm) or the solvent residential signal (δ 7.26 ppm for $CDCl_3$, δ 1.94 ppm for CD_3CN) was used in calibration for ^1H experiments, and an NMR solvent signal (δ 77.16 ppm for $CDCl_3$) was used as a reference for ^{13}C experiments. Mass spectra were recorded using an LTQ XL Orbitrap instrument or a Q Exactive Quadruple-Orbitrap instrument (ThermoFisher) in the ESI (positive ion) mode in the Vanderbilt Mass Spectrometry Core Facility. For HRMS, the LTQ XL Orbitrap instrument was set to full scan mode, FTMS analyzer, 60,000 resolution, and scanned from *m/z* 100 to 1000. Column chromatography was

performed using SiliaFlash F60 (230-400 mesh), and analytical TLC was done with SilicaGel 60 F_{254} plates (both from Merck KgaA).

*Synthesis of 3,7-dimethyloct-6-enal-1-*d *(citronellal-*d*, **1**)*

Methyl 3,7-dimethyloct-6-enoate

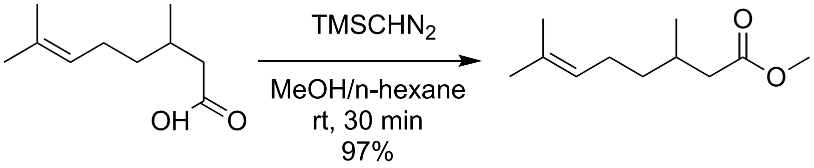

Trimethylsilyl diazomethane (ca. 0.6 M in *n*-hexane, 12 mL) was added slowly to a solution of 3,7-dimethyloct-6-enoic acid (681 mg, 4.0 mmol) in CH_3OH (10 mL) and stirred at room temperature for 30 min. After quenching the reaction by the addition of CH_3CO_2H, the mixture was diluted with EtOAc (100 mL). The resulting mixture was washed 3× with saturated $NaHCO_3$ (aq.) and brine, dried over anhydrous Na_2SO_4, and evaporated to obtain the methyl ester as a colorless oil (712 mg, 97%). This product was used without further purification. ^{1}H NMR (600 MHz, $CDCl_3$): δ 0.94 (d, 3H, *J*=6.7 Hz), 1.19–1.25 (m, 1H), 1.32–1.38 (m, 1H), 1.60 (s, 3H), 1.68 (s, 3H), 1.93–2.03 (m, 3H), 2.12 (dd, 1H, *J*=14.7, 8.3 Hz), 2.32 (dd, 1H, *J*=14.7, 5.9 Hz), 3.67 (s, 3H), 5.09 (tt, 1H, *J*=7.1, 1.3 Hz).

3,7-Dimethyloct-6-en-1,1-d_2-1-ol[1]

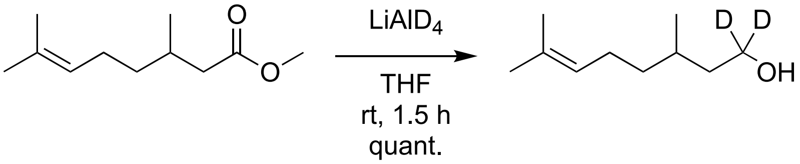

$LiAlD_4$ (486 mg, 12 mmol) was added in portions to a solution of the above methyl ester (712 mg, 3.9 mmol) in anhydrous THF (20 mL) at 0 °C. The mixture was warmed to room temperature and stirred for 1.5 h, and then excess Na_2SO_4-10 H_2O was added to destroy the excess $LiAlD_4$. The resulting mixture was diluted with EtOAc, filtered through Celite, and washed with EtOAc and the filtrate was evaporated to obtain the crude deuterated alcohol as a colorless oil (639

mg, quantitative yield). The product was used without further purification. ^{1}H NMR (600 MHz, $CDCl_3$): δ 0.91 (d, *J*=6.6 Hz, 3H), 1.14–1.21 (m, 1H), 1.31–1.39 (m, 2H), 1.54–1.62 (m, 2H), 1.60 (s, 3H), 1.68 (s, 3H), 1.93–2.04 (m, 2H), 5.10 (tt, 1H, *J*=7.1, 1.3 Hz). ^{13}C NMR (150 MHz, $CDCl_3$): δ 17.5, 19.5, 25.5, 25.7, 29.1, 37.2, 39.7, 60.5 (quint, *J*=21 Hz), 124.7, 131.3.

3,7-Dimethyloct-6-enal-1-*d* (citronellal-*d*, **1**)

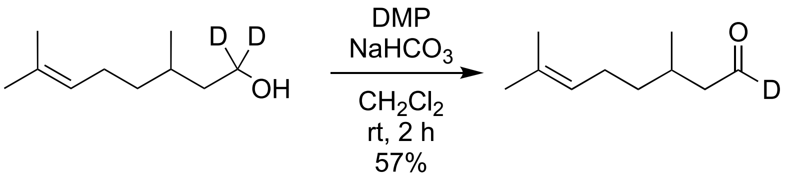

Dess-Martin periodinane[2] (1.27 g, 3.0 mmol) was added in portions to a mixture of the above deuteride alcohol (236 mg, 1.5 mmol) and $NaHCO_3$ (756 mg, 9.0 mmol) in anhydrous CH_2Cl_2 (10 mL) and the reaction mixture was stirred at room temperature for 2 h. The reaction was quenched by the addition of a 1:1 mixture of saturated $NaHCO_3$ (aq) and saturated $Na_2S_2O_3$ (aq) (40 mL,v/v). The resulting mixture was extracted 3× with CH_2Cl_2 and the organic layer was washed with brine, dried over anhydrous Na_2SO_4, and evaporated. The crude product was purified by silicic acid gel column chromatography (hexanes:$CHCl_3$, 1:1, v/v) to obtain the deuterated aldehyde as a colorless oil (174 mg, 57%). ^{1}H NMR (600 MHz, $CDCl_3$): δ 0.97 (d, 3H, *J*=6.9 Hz), 1.24–1.30 (m, 1H), 1.35–1.39 (m, 1H), 1.60 (s, 3H), 1.69 (s, 3H), 1.95–2.10 (m, 3H), 2.23 (dd, 1H, *J*=16.0, 8.0 Hz), 2.40 (dd, 1H, *J*=16.0, 5.6 Hz) 5.10 (t, 1H, *J*=7.0 Hz). ^{13}C NMR (150 MHz, $CDCl_3$): δ 17.7, 19.9, 25.4, 25.7, 27.8, 37.0, 50.8 (t, *J*=3.5 Hz), 124.0, 131.8, 202.8 (t, *J*=25 Hz). HRMS-ESI (*m/z*): $[M+H]^+$ calcd for $C_{10}H_{18}{}^{2}HO$, 156.1493; found, 156.1491 (Δ –1.2 ppm).

*Synthesis of phenyl aldehydes (**2**, **3**)*

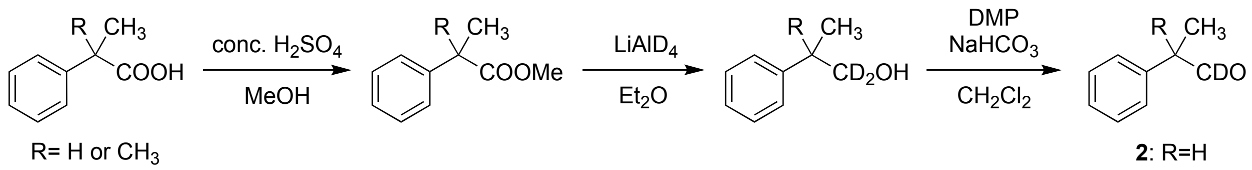

2: R=H
3: R=CH3

General procedure for synthesis of methyl esters

H_2SO_4 (98%, 30 μL mL^{-1}) was added to a solution of the carboxylic acid in CH_3OH (2.0 M) at 0 °C. The mixture was heated at reflux for 1–2 h and then cooled to 0 °C. Saturated $NaHCO_3$ (aq). was added to quench the reaction, which was then extracted with EtOAc. The organic layer was washed with brine, dried over anhydrous Na_2SO_4, and evaporated to obtain the crude methyl ester (93–96% yield), which was used without further purification.

Methyl 2-phenylpropionate

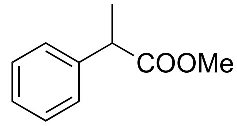

^1H NMR (400 MHz, $CDCl_3$): δ 1.50 (d, 3H, *J*=7.2 Hz), 3.65 (s, 3H), 3.72 (q, 1H, *J*=7.2 Hz), 7.22–7.34 (m, 5H).

Methyl 2-methyl-2-phenylpropionate

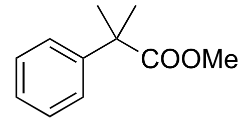

^1H NMR (400 MHz, $CDCl_3$): δ 1.58 (s, 6H), 3.63 (s, 3H), 7.21–7.24 (m, 1H), 7.29–7.34 (m, 4H).

General procedure for the synthesis of alcohols[3]

The methyl ester was added to a suspension of $LiAlD_4$ in anhydrous Et_2O, dropwise at 0 °C. The mixture was warmed to room temperature and stirred for 0.5–3 h before adding excess

Na_2SO_4-10 H_2O to quench the reaction. The resulting mixture was diluted with EtOAc and filtered, then washed with EtOAc, and the filtrate was evaporated to obtain the crude deuterated alcohol.

2-Phenylpropan-1,1-d_2-1-ol

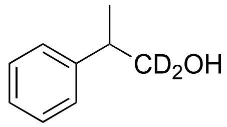

The compound was prepared from methyl 2-phenylpropionate (657 mg, 4.0 mmol), $LiAlD_4$ (252 mg, 6.0 mmol), and Et_2O (10 mL). The crude product was purified by silicic acid column chromatography (hexanes/CH_2Cl_2, 1/1, v/v) to yield the alcohol as a colorless oil (419 mg, 76%). ^{1}H NMR (600 MHz, $CDCl_3$): δ 1.26 (d, 3H, *J*=7.2 Hz), 1.59 (brs, 1H), 2.90 (q, 1H, *J*=7.2 Hz), 7.21–7.23 (m, 3H), 7.30–7.33 (m, 2H). ^{13}C NMR (150 MHz, $CDCl_3$): δ 17.6, 42.3, 68.0 (quin, *J*=22 Hz), 126.7, 127.6, 128.7, 143.8.

2-Methyl-2-phenylpropan-1,1-d_2-1-ol

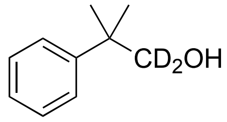

The compound was prepared from methyl 2-methyl-2-phenylpropionate (540 mg, 3.0 mmol), $LiAlD_4$ (189 mg, 4.5 mmol), and Et_2O (8.0 mL). The alcohol, obtained as a yellow oil, was used for the next step without further purification (409 mg, 89% yield). ^{1}H NMR (600 MHz, $CDCl_3$): δ 1.30 (s, 6H), 1.52 (brs, 1H), 7.18–7.21 (m, 1H), 7.30–7.36 (m, 4H). ^{13}C NMR (150 MHz, $CDCl_3$): δ 25.3, 39.9, 72.3 (quin, *J*=22 Hz), 126.2, 126.3, 128.5, 146.5.

General procedure for the synthesis of aldehydes[4]

NaHCO$_3$ and Dess-Martin periodinane (DMP) were added to a solution of the alcohol in CH_2Cl_2 (0.1 M) at 0 °C. The resulting mixture was stirred at room temperature for 1–2 h and then quenched by the addition of a 1:1 mixture (v/v) of saturated $NaHCO_3$ (aq). and saturated $Na_2S_2O_3$

(aq). The resulting mixture was extracted 3× with CH_2Cl_2 and the organic layer was washed with brine, dried over anhydrous Na_2SO_4, and evaporated. The crude product was purified by silicic acid column chromatography (hexanes/CH_2Cl_2, 1/1, v/v).

2-Phenylpropionaldehyde-*d* (**2**)

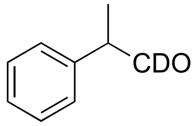

The compound was prepared from 2-phenylpropan-1,1-d_2-1-ol (276 mg, 2.0 mmol), DMP (1.02 g, 2.4 mmol), $NaHCO_3$ (252 mg, 3.0 mmol), and anhydrous CH_2Cl_2 (20 mL). The aldehyde was obtained as a colorless oil (128 mg, 47% yield). ^{1}H NMR (600 MHz, $CDCl_3$): δ 1.45 (d, 3H, *J*=7.1 Hz), 3.63 (q, 1H, *J*=7.1 Hz), 7.21 (d, 2H, *J*=7.5 Hz), 7.30 (t, 1H, *J*=7.4 Hz), 7.38 (t, 2H, *J*=7.6 Hz). ^{13}C NMR (150 MHz, $CDCl_3$): δ 14.7, 53.0 (t, *J*=3.4 Hz), 127.7, 128.5, 129.2, 137.9, 201.0 (t, *J*=27 Hz). HRMS-ESI (*m/z*): $[M+H]^+$ calcd for $C_9H_{10}{}^2HO$, 136.0867; found, 136.0866 (Δ –0.6 ppm).

2-Methyl-2-phenylpropionaldehyde-*d* (**3**)

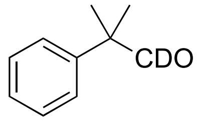

The compound was prepared from 2-methyl-2-phenylpropan-1,1-d_2-1-ol (304 mg, 2.0 mmol), DMP (509 mg, 1.2 mmol), $NaHCO_3$ (126 mg, 1.5 mmol), and anhydrous CH_2Cl_2 (10 mL). The aldehyde was obtained as a colorless oil (209 mg, 70% yield). ^{1}H NMR (600 MHz, $CDCl_3$): δ 1.47 (s, 6H), 7.27–7.30 (m, 3H), 7.38 (t, 2H, *J*= 7.7 Hz). ^{13}C NMR (150 MHz, $CDCl_3$): δ 22.6, 50.5 (t, *J*=3.1 Hz), 126.8, 127.4, 129.0, 141.3, 202.1 (t, *J*=27 Hz). HRMS-ESI (*m/z*): $[M+H]^+$ calcd for $C_{10}H_{12}{}^2HO$, 150.1024; found, 150.1025 (Δ 0.7 ppm).

*Synthesis of 3-(pyridin-3-yl)propyl esters (**6a**, 7**a**, **8a**, **9a**)*

General procedure

N,*N*-Dimethyl-4-aminopyridine (DMAP) and 3-pyridinepropanol were added to a solution of the carboxylic acid and *N*,*N*′-dicyclohexylcarbodiimide (DCC) in CH_2Cl_2 at room temperature. The reaction mixture was stirred for 1 h, unless otherwise noted, before removing the white precipitate by filtration. The filtrate was evaporated and the resulting crude product was purified by silicic acid column chromatography.

3-(Pyridin-3-yl)propyl formate-*d* (**6a**): The compound was prepared from formic acid-*d* (95% in

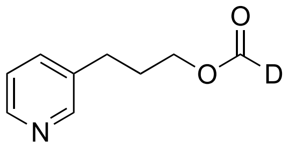

D_2O, 240 μL, 6.0 mmol), 3-pyridinepropanol (480 mg, 3.5 mmol), DCC (1.2 g, 6.0 mmol), and DMAP (74 mg, 0.6 mmol) in CH_2Cl_2 (50 mL). The product was purified by silicic acid chromatography using hexanes:EtOAc (2:3, v/v) as a mobile phase and isolated as a colorless oil (551 mg, 95%). ^{1}H NMR (600 MHz, $CDCl_3$): δ 1.98-2.04 (m, 2H), 2.71-2.74 (m, 2H), 4.20 (t, 2H, *J*=6.4 Hz), 7.26 (dd, 1H, *J*=7.8, 4.9 Hz), 7.50 (d, 1H, *J*=7.9 Hz), 8.46-8.47 (m, 2H). ^{13}C NMR (150 MHz, $CDCl_3$): δ 29.1, 29.7, 62.6, 123.5, 136.2, 147.1, 149.2, 160.7 (t, *J*=34.4 Hz). HRMS-ESI (*m/z*): $[M+H]^+$ calcd for $C_{10}H_{12}{}^2HO$, 150.1024; found, 150.1025 (Δ 0.7 ppm).

3-(Pyridin-3-yl)propyl 3,7-dimethyloct-6-enoate (**7a**): The compound was prepared from

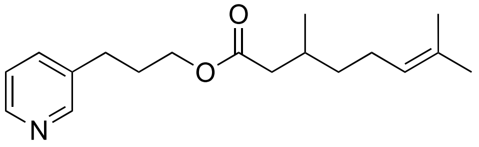

citronellic acid (341 mg, 2.0 mmol), 3-pyridinepropanol (412 mg, 3.0 mmol), DCC (619 mg, 3.0 mmol), and DMAP (37 mg, 0.3 mmol) in CH_2Cl_2 (20 mL). The product was purified by silicic acid chromatography using hexanes:EtOAc (2:1, v/v) as a mobile phase and isolated as a colorless oil

(396 mg, 68%). ^{1}H NMR (600 MHz, $CDCl_3$): δ 0.96 (d, 3H, *J*=6.7 Hz), 1.21–1.27 (m, 1H), 1.33–1.39 (m, 1H), 1.60 (s, 3H), 1.68 (s, 3H), 1.94–2.05 (m, 5H), 2.13 (dd, H, *J*=14.7, 8.2 Hz), 2.32 (dd, 1H, *J*=14.7, 5.9 Hz), 2.70 (t, 2H, *J*=7.8 Hz), 4.11 (t, 2H, *J*=6.5 Hz), 5.09 (tt, 1H, *J*=7.1, 1.3 Hz), 7.22 (dd, 1H, *J*=7.8, 4.9 Hz), 7.50 (td, 1H, *J*=7.7, 1.8 Hz), 8.45–8.46 (m, 2H). ^{13}C NMR (150 MHz, $CDCl_3$): δ 17.1, 19.7, 25.5, 25.8, 30.08, 30.13, 36.9, 41.9, 63.4, 123.4, 124.3, 131.7, 135.9, 136.6, 147.7, 150.0, 173.3. HRMS-ESI (*m/z*): $[M+H]^+$ calcd for $C_{18}H_{28}HO_2N$, 290.2115; found, 290.2127 (Δ 4.4 ppm).

3-(Pyridin-3-yl)propyl 2-phenylpropionate (**8a**): This compound was prepared from 2-phenylpropionic acid (150 mg, 1.0 mmol), 3-pyridinepropanol (206 mg, 1.5 mmol), DCC (309 mg, 1.5 mmol), and DMAP (18 mg, 0.15 mmol) in CH_2Cl_2 (15 mL). The product was purified by silicic acid chromatography using hexanes:EtOAc (2:1, v/v) as a mobile phase and isolated as a colorless oil (222 mg, 83%). ^{1}H NMR (600 MHz, $CDCl_3$): δ 1.51 (d, 3H, *J*=7.2 Hz), 1.86–1.91 (m, 2H), 2.52 (t, 2H, *J*= 7.7 Hz), 3.73 (q, 1H, *J*=7.2 Hz), 4.03–4.10 (m, 2H), 7.17 (dd, 1H, *J*=7.7, 4.8 Hz), 7.26–7.28 (m, 1H), 7.31–7.35 (m, 5H), 8.32 (s, 1H), 8.43 (d, 1H, *J*=4.7 Hz). ^{13}C NMR (150 MHz, $CDCl_3$): δ 18.4, 29.2, 30.0, 45.7, 63.6, 123.4, 127.4, 127.6, 128.8, 135.9, 136.5, 140.7, 147.7, 150.0, 174.6. HRMS-ESI (*m/z*): $[M+H]^+$ calcd for $C_{17}H_{20}HO_2N$, 270.1489; found, 270.1505 (Δ 6.1 ppm).

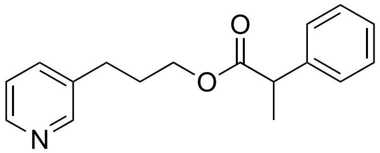

3-(Pyridin-3-yl)propyl 2-methyl-2-phenylpropionate (**9a**): The compound was prepared from 2-methylphenylpropionic acid (164 mg, 1.0 mmol), 3-pyridinepropanol (206 mg, 1.5 mmol), DCC (309 mg, 1.5 mmol), and DMAP (18 mg, 0.15 mmol) in CH_2Cl_2 (15 mL). The product

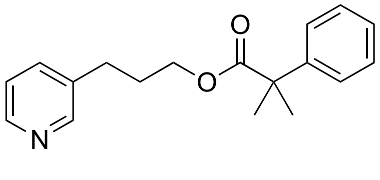

was purified by silicic acid chromatography using hexanes:EtOAc (2:1, v/v) as a mobile phase and isolated as a colorless oil (140 mg, 50%). ^{1}H NMR (600 MHz, $CDCl_3$): δ 1.60 (s, 6H), 1.86 (tt, 2H, *J*=7.4, 6.6 Hz), 2.48 (t, 2H, *J*= 7.7 Hz), 4.06 (t, 2H, *J*=6.2 Hz), 7.16 (dd, 1H, *J*=7.7, 4.9 Hz), 7.25 (t, 1H, *J*=6.8 Hz), 7.31–7.38 (m, 5H), 8.30 (s, 1H), 8.42 (d, 1H, *J*=4.7 Hz). ^{13}C NMR (150 MHz, $CDCl_3$): δ 26.4, 29.1, 29.9, 46.6, 63.5, 123.4, 125.7, 126.8, 128.5, 135.9, 136.4, 144.7, 147.6, 150.0, 176.7. HRMS-ESI (*m/z*): $[M+H]^+$ calcd for $C_{18}H_{22}HO_2N$, 284.1645; found, 284.1659 (Δ 4.8 ppm).

*Synthesis of possible Baeyer-Villiger intermediates (**4**, **10**, **11**)*

2,6-Dimethylhept-5-en-1-yl formate-*d* (**4**).

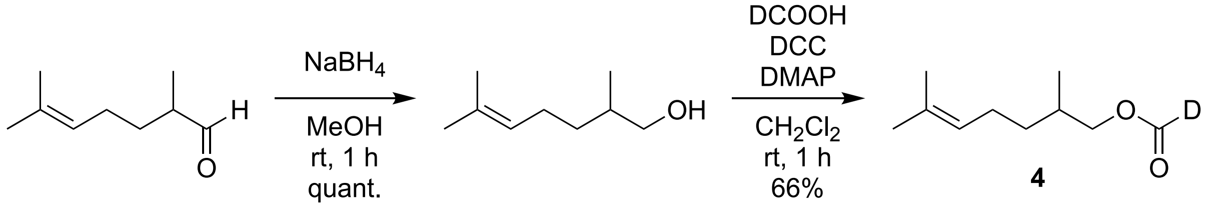

$NaBH_4$ (378 mg, 10 mmol) was added to a solution of 2,6-dimethylhept-5-enal (1.4 g, 10 mmol) in CH_3OH (20 mL) and the resulting solution was stirred at room temperature for 1 h. Saturated $NaHCO_3$ (aq). was added and the mixture was extracted 3× with CH_2Cl_2 and the organic layer was washed with brine, dried over anhydrous Na_2SO_4, and evaporated to obtain the crude alcohol (1.57 g), which was used for the next step without further purification.

The alcohol (363 mg, 2.6 mmol) was dissolved in CH_2Cl_2 (50 mL). DCC (1.05 g, 5.1 mmol) and DMAP (62.3 mg, 0.51 mmol) were added to the mixture, which was stirred at room temperature. Formic acid-*d* (95% in D_2O, 204 µL, 5.1 mmol) was added slowly into the mixture. After stirring at room temperature for 1 h, the precipitate was filtered, and the filtrate was

evaporated. The obtained crude material was purified by silicic acid column chromatography (hexanes/CH_2Cl_2, 1/1, v/v) to yield the ester as a colorless oil (286 mg, 66%). ^{1}H NMR (600 MHz, $CDCl_3$): δ 0.96 (d, 3H, *J*=6.8 Hz), 1.18–1.24 (m, 1H), 1.40–1.46 (m, 1H), 1.61 (s, 3H), 1.69 (s, 3H), 1.83 (sep, 1H, *J*=6.6 Hz), 1.95–2.07 (m, 2H), 3.97 (dd, 1H, *J*=10.7, 6.8 Hz), 4.06 (dd, 1H, *J*=10.8, 5.8 Hz) 5.08 (t, 1H, *J*=6.8 Hz). ^{13}C NMR (150 MHz, $CDCl_3$): δ 16.8, 17.8, 25.3, 25.8, 32.2, 33.4, 68.8, 124.2, 131.9, 161.2 (t, *J*=34 Hz).

1-Phenylethyl formate-*d* and 2-phenylpropan-2-yl formate-*d*.

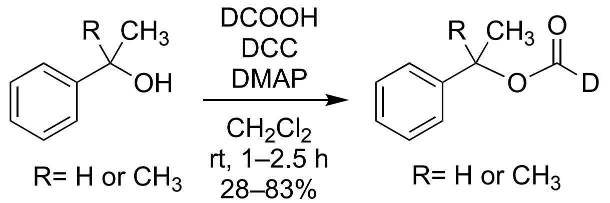

The alcohol (1.0 mmol) was dissolved in CH_2Cl_2 (10 mL). DCC (413 mg, 2.0 mmol) and DMAP (24.4 mg, 0.20 mmol) were added to the mixture, which was stirred at room temperature. Formic acid-*d* (95% in D_2O, 80 μL, 2.0 mmol) was added slowly. After stirring at room temperature for 1–2.5 h, the precipitate was filtered and the filtrate was evaporated. The crude product was purified by silicic acid column chromatography (hexanes/CH_2Cl_2, 2/3, v/v) to yield the ester.

1-Phenylethyl formate-*d* (**10**): The product was synthesized from 1-phenylethanol (122 mg, 1.0 mmol); the reaction was stirred for 1 h. The product was obtained as a colorless oil (125 mg, 83%). ^{1}H NMR (600 MHz, $CDCl_3$): δ 1.58 (d, 3H, *J*=6.7 Hz), 6.00 (q, 1H, *J*=6.6 Hz), 7.29–7.32 (m, 1H), 7.34–7.38 (m, 4H). ^{13}C NMR (150 MHz, $CDCl_3$): δ 22.2, 72.3, 126.3, 128.3, 128.7, 141.0, 160.0 (t, *J*=34 Hz).

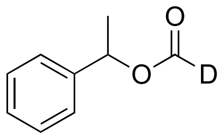

2-Phenylpropan-2-yl formate-*d* (**11**): The product was synthesized from 2-phenyl-2-propanol (136

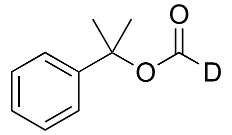

mg, 1.0 mmol); the reaction was stirred for 2.5 h. The product was obtained as a colorless oil (46 mg, 28%). ^{1}H NMR (600 MHz, $CDCl_3$): δ 1.81 (s, 6H), 7.25–7.29 (m, 1H), 7.34–7.40 (m, 4H). ^{13}C NMR (150 MHz, $CDCl_3$): δ 29.0, 82.6, 124.7, 127.6, 128.6, 145.0, 160.4 (t, *J*=34 Hz).

*Synthesis of precursor (nitrosourea) of diazo reagent (**5**)*[5]

Caution! *Diazo reagent **5** and its nitroso precursor are strong alkylating agents and expected to be toxic, mutagenic, and possibly carcinogenic. Handle in a fume hood with adequate ventilation and appropriate skin protection!*

1-(3-(Pyridin-3-yl)propyl)urea

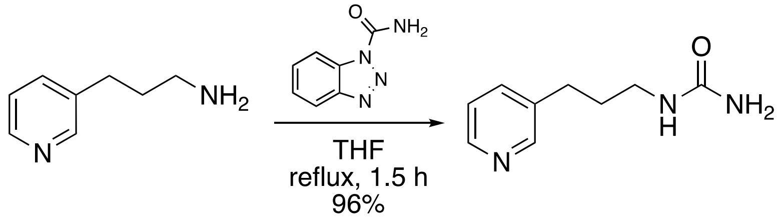

3-(Pyridine-3-yl)propan-1-amine (1.2 g, 8.7 mmol) was added to a solution of benzotriazole-1-carboxamide (1.5 g, 9.2 mmol) in THF (40 mL) and the mixture was heated under reflux for 1.5 h. After cooling to room temperature, the precipitate was filtered. The precipitate was resuspended in CH_3OH and filtered again, and the filtrate was evaporated to obtain the urea as a white solid (1.5 g, 96%). This product was used without further purification. The ^{1}H NMR spectrum matched our previous report.[5] ^{1}H NMR (600 MHz, CD_3CN): 1.74 (sep, 2H, *J*=7.4 Hz),

2.62 (t, 2H, *J*=7.9 Hz), 3.07 (q, 2H, *J*=6.8 Hz), 4.53 (brs, 2H), 5.10 (brs, 1H), 7.25 (dd, 1H, *J*=7.7, 4.8 Hz), 7.58 (dt, 1H, *J*=7.8, 1.7 Hz), 8.39 (dd, 1H, *J*=4.7, 1.4 Hz), 8.43 (d, 1H, *J*=1.9 Hz).

1-Nitroso-1-(3-(pyridin-3-yl)propyl)urea

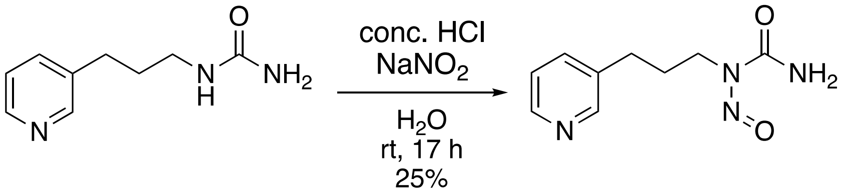

Concentrated HCl (1.0 mL) was added to a solution of the above urea (450 mg, 2.5 mmol) in water (20 mL) at 0 °C. The mixture was stirred at 0 °C for a few minutes and $NaNO_2$ (215 mg, 3.1 mmol) was then added in portions. The resulting mixture was warmed to room temperature and stirred for 16 h. CH_2Cl_2 (50 mL) and saturated $NaHCO_3$ (aq). (50 mL) were added, and the entire mixture was extracted 3× with CH_2Cl_2 (50 mL). The organic layer was washed with brine, dried over anhydrous Na_2SO_4, and evaporated to yield the nitrosourea as a yellow paste (129 mg, 25%). The ^1H NMR spectrum matched our previous report.[5] ^1H NMR (600 MHz, CD_3CN): 1.69 (sep, 2H, *J*=7.6 Hz), 2.55 (t, 2H, *J*=7.8 Hz), 3.78 (t, 2H, *J*=7.3 Hz), 6.11 (brs, 1H), 7.04 (brs, 1H), 7.25 (dd, 1H, *J*=7.7, 4.8 Hz), 7.56 (dt, 1H, *J*=7.7, 2.0 Hz), 8.38–8.40 (m, 2H).

The product was dissolved in CH_2Cl_2, aliquoted into 2 mL amber vials, and then dried under a nitrogen stream to leave ~3 mg of compound in each vial. These aliquots were used for preparing the diazo reagent **5** before use (as described in p. S-20), without further purification.

Synthesis of 3-(pyridin-3-yl)propyl esters of potential products of ***8a*** ***(M1*** *and* ***M2)***

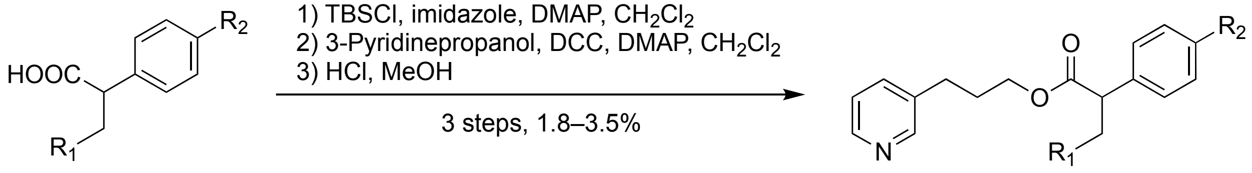

M1: R_1=OH, R_2=H
M2: R_1=H, R_2=OH

3-(Pyridin-3-yl)propyl 3-hydroxy-2-phenylpropanoate (**M1**)

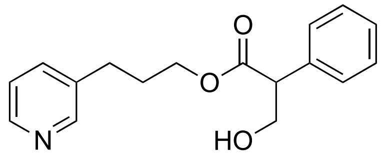

Imidazole (272 mg, 4.0 mmol), DMAP (24 mg, 0.2 mmol), and *tert*-butyldimethylsilyl chloride (332 mg, 2.2 mmol) were added to a solution of DL-tropic acid (332 mg, 2.0 mmol) in CH_2Cl_2 (5.0 mL), and the resulting mixture was stirred overnight at room temperature. The reaction was quenched by the addition of saturated NH_4Cl (aq), and the entire mixture was extracted 3× with Et_2O. The organic layer was washed with brine, dried over anhydrous Na_2SO_4, and evaporated to yield the crude TBS ether.

The crude material was dissolved in CH_2Cl_2 (10 mL) and DCC (619 mg, 3.0 mmol), DMAP (36 mg, 0.30 mmol), and 3-pyridinepropanol (412 mg, 2.0 mmol) were added. After stirring at room temperature for 2 h, the precipitate was filtered and the filtrate was evaporated. The crude product was purified by silicic acid column chromatography (hexanes/EtOAc, 10/1→3/1, v/v) to yield the ester as a colorless oil (89 mg).

The 3-(pyridin-3-yl)propyl ester obtained above was dissolved in CH_3OH (2.0 mL) and 1 M HCl (aq). (2.0 mL) was added. The resulting mixture was stirred at room temperature for 10 min before washing with $CHCl_3$ (10 mL). The aqueous layer was neutralized by saturated $NaHCO_3$ (aq). and extracted with $CHCl_3$ (3×5 mL). The organic layer was washed with brine, dried over

anhydrous Na_2SO_4, and evaporated to yield **M1** as a yellow paste (20 mg, 3.5 % yield over the 3 steps). ^{1}H NMR (600 MHz, $CDCl_3$): δ 1.88–1.93 (m, 2H), 2.54 (t, 2H, *J*= 7.7 Hz), 3.83–3.87 (m, 2H), 4.10–4.19 (m, 3H), 7.17 (dd, 1H, *J*=7.3, 5.0 Hz), 7.29–7.32 (m, 3H), 7.34–7.37 (m, 2H), 8.31 (s, 1H), 8.42 (d, 1H, *J*=4.3 Hz). ^{13}C NMR (150 MHz, $CDCl_3$): δ 29.2, 29.9, 54.2, 63.9, 64.5, 123.5, 127.9, 128.3, 129.0, 135.8, 136.0, 136.4, 147.6, 149.9, 173.2. HRMS-ESI (*m/z*): $[M+H]^+$ calcd for $C_{17}H_{20}HO_3N$, 286.1438; found, 286.1436 (Δ –0.6 ppm).

3-(Pyridin-3-yl)propyl 2-(4-hydroxyphenyl)propanoate (**M2**)

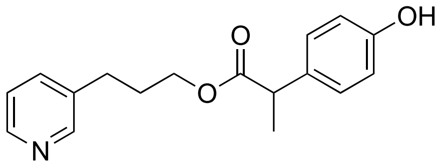

Imidazole (272 mg, 4.0 mmol), DMAP (24 mg, 0.2 mmol), and *tert*-butyldimethylsilyl chloride (332 mg, 2.2 mmol) were added to a solution of 2-(4-hydroxyphenyl)propionic acid (332 mg, 2.0 mmol) in CH_2Cl_2/DMF(5.0/0.5 mL), and the resulting mixture was stirred overnight at room temperature. The reaction was quenched by the addition of saturated NH_4Cl (aq), and the entire mixture was extracted 3× with Et_2O. The organic layer was washed with brine, dried over anhydrous Na_2SO_4, and evaporated to yield the crude TBS ether.

The crude material was dissolved in CH_2Cl_2 (10 mL), and DCC (619 mg, 3.0 mmol), DMAP (36 mg, 0.30 mmol), and 3-pyridinepropanol (412 mg, 2.0 mmol) were added. After stirring at room temperature for 2 h, the precipitate was filtered and the filtrate was evaporated. The crude product was purified by silicic acid column chromatography (hexanes/EtOAc, 3/1→1/2, v/v) to yield the ester as a white solid (126 mg).

The 3-(pyridin-3-yl)propyl ester obtained above was dissolved in CH_3OH (4.0 mL) nd 4.0 mL of 1 M HCl (aq). was added. The resulting mixture was stirred at room temperature for 30 min before washing with $CHCl_3$ (10 mL). The aqueous layer was neutralized by saturated $NaHCO_3$

(aq) and extracted with $CHCl_3$ (3×5 mL). The organic layer was washed with brine, dried over anhydrous Na_2SO_4, and evaporated to yield **M1** as a yellow paste (10 mg, 1.8 % yield over the 3 steps). ^1H NMR (600 MHz, $CDCl_3$): δ 1.48 (d, 3H, *J*=7.0 Hz), 1.83–1.89 (m, 2H), 2.50 (t, 2H, *J*= 7.7 Hz), 3.66 (q, 1H, *J*=7.3 Hz), 3.99–4.03 (m, 1H), 4.11–4.15 (m, 1H), 6.84 (d, 2H, *J*=8.1 Hz), 7.17 (d, 2H, *J*=8.1 Hz), 7.20–7.22 (m, 1H), 7.41 (d, 1H, *J*=7.7 Hz), 8.22 (s, 1H), 8.41 (s, 1H). ^{13}C NMR (150 MHz, $CDCl_3$): δ 18.2, 29.2, 30.0, 44.9, 63.3, 116.0, 123.8, 128.8, 132.2, 136.5, 137.0, 146.9, 149.4, 156.0, 175.0. HRMS-ESI (*m/z*): $[M+H]^+$ calcd for $C_{17}H_{20}HO_3N$, 286.1438; found, 286.1435 (Δ –0.8 ppm).

2. Enzyme assays

Instruments

UV-visible spectra were recorded using an OLIS-Aminco DW2a instrument (On-Line Instrument Systems, Athens, GA) in the split-beam mode.

For LC-MS analyses, a Vanquish Horizon UHPLC system connected to a Q-Exactive HF Quadrupole-Orbitrap mass spectrometer with a heated electrospray ionization probe was used (ThermoScientific) in the Vanderbilt Mass Spectrometry Core Facility. LC-UV analyses were performed using Waters ACQUITY ultra-performance liquid chromatography (UPLC) systems with a Waters ACQUITY UPLC photodiode array (PDA) detector.

Reagents

$^{18}O_2$ gas (97 atom % ^{18}O) was purchased from Sigma-Aldrich (Cat. No. 490474). $H_2{}^{18}O$ (97 atom % ^{18}O) was purchased from Cambridge Isotope Laboratories (Cat. No. OLM-240-97-1). D_2O (99.8 atom % D) was purchased from Alfa Aesar (Ward Hill, MA). All other reagents used for enzyme incubations were of analytical grade, and solvents for LC-UV or LC-MS analyses were of LC-MS grade.

Et_2O and *tert*-butyl methyl ether (^{t}BME) were filtered through basic alumina before use to remove endogenous formic acid.

Expression of P450 2B4

A cDNA was constructed with codon optimization for expression in *Escherichia coli*, using overlapping oligonucleotides[6] and assembled as done elsewhere,[7] done by Dr. Zhong-Liu Wu, formerly of this laboratory. The nucleotide sequence of the cDNA is shown in Figure S1.

The P450 2B4 plasmid (cloned into a pCW vector) was transformed along with molecular chaperones GroES/GroEL into *E. coli* DH5α cells according to a standard heat-shock protocol.[8] An aliquot of the transformation (200 μL, in Super Optimal broth with Catabolite repression (S.O.C.) media) was plated on Luria-Bertani (LB) agar supplemented with antibiotics (ampicillin and kanamycin) and was incubated overnight (37 °C). A single colony was used to inoculate LB media (100 mL) supplemented with ampicillin (0.1 mg mL^{-1}) and kanamycin (0.05 mg mL^{-1}) and the pre-culture was grown overnight (37 °C, 220 rpm). The pre-culture (10 mL) was then used to inoculate Terrific Broth (TB) media (1 L, in 2.8 L Fernbach flasks) supplemented with NaCl (2 mM), trace elements solution (0.25% (v/v)[8]), ampicillin (0.1 mg mL^{-1}), kanamycin (0.05 mg mL^{-1}) and thiamine (2 mM). Bulk cultures were incubated (37 °C, 220 rpm) for 2 h prior to induction of (i) P450 expression with IPTG (1 mM), (ii) heme biosynthesis with 5-aminolevulinic acid (0.5 mM), and (iii) GroES/GroEL expression with arabinose (1 mg mL^{-1}). The cultures were incubated (28 °C, 220 rpm) for 42 h.

Cultures were decanted into centrifuge bottles and harvested via centrifugation (5000 × *g*, 4 °C, 15 min). Cell pellets were resuspended (15 mL (g cell pellet)$^{-1}$) in 2× TES buffer (100 mM Tris-acetate buffer, pH 7.5, containing sucrose (0.5 M) and EDTA (1 mM)) until homogenous. The suspension was treated with lysozyme (120 mg L^{-1} suspension), diluted 2-fold with ice-cold H_2O, and incubated on ice (30 min) with occasional mixing. The centrifugation process was repeated, the supernatant was decanted, and the spheroplast pellets were resuspended in sonication

buffer (potassium phosphate buffer (100 mM, pH 8.0) containing glycerol (20% v/v), magnesium acetate (6 mM), and 2-mercaptoethanol (10 mM)) and stored at -80 °C until purification.

Purification of P450 2B4

Spheroplast solutions were thawed on ice and supplemented with protease inhibitors (phenylmethylsufonyl fluoride (1 mM, from 100 mM stock in isopropanol), leupeptin (2 μM), aprotonin (0.04 U mL^{-1}), and bestatin (1 μM)). The mixture was subjected to sonication (3 × 20 min bursts, 50% power) and the lysate was centrifuged (10^4 ×*g*, 4 °C, 20 min). The supernatant (cloudy red color) was decanted and subjected to ultracentrifugation (10^5 ×*g*, 4 °C, 90 min). Membrane pellets were resuspended in buffer (phosphate buffer (100 mM, pH 7.4) containing glycerol (20%, v/v) and 2-mercaptoethanol (10 mM)) to a final volume of ~300 mL and assayed for total protein with a bicinchoninic acid (BCA) assay, using a standard protocol.[9] The resuspension was diluted to a protein concentration of 2 mg mL^{-1} (~5-fold dilution, to ~1.5 L) in solubilization buffer (20 mM potassium phosphate buffer, pH 7.4, containing glycerol (20%, v/v), KCl (0.5 M), 2-mercaptoethanol (10 mM), sodium cholate (0.625%, w/v), Emulgen 911 (1.25%, w/v), and imidazole (5 mM)) and stirred overnight (at 4 °C).

The solubilized membranes (1.5 L volume) were subjected to ultracentrifugation again (10^5 × *g*, 4 °C, 90 min), and the supernatant was applied to a Ni^{2+}-NTA column (1.5 cm × 7 cm, ~12 mL) equilibrated with solubilization buffer (10 column volumes). When protein loading was complete, the column was washed with solubilization buffer supplemented with 20 mM imidazole (10 column volumes). P450 2B4 was then eluted with the solubilization buffer but (i) devoid of Emulgen 911, (ii) with a reduced concentration of sodium cholate (0.5% (w/v)), and (iii) with an increased concentration of imidazole (400 mM). Colored column fractions were pooled and

dialyzed (3×) against 2 L of potassium phosphate buffer (100 mM, pH 7.4) containing glycerol (20%, v/v) and EDTA (1 mM)), and the protein was stored at -80 °C. This method yielded 80 nmol of purified P450 2B4 protein per 1 L cell culture.

The purity of the P450 2B4 preparation was analyzed by sodium dodecyl sulfate-polyacrylamide gel electrophoresis (Figure S2). The reduced-carbon monoxide vs. reduced difference spectrum is shown in Figure S2, indicating that most of the protein was P450 and not cytochrome P420.

The purified P450 was subjected to trypsin cleavage and the peptides were analyzed in the proteomics facility in the Vanderbilt Mass Spectrometry Research Center. A list of identified peptides is presented in Table S1, and the coverage of the predicted amino acid sequence is shown in Figure S1.

Preparation of other enzymes

Human P450 17A1 was expressed in *E. coli* and purified as described previously.[10] Rat NADPH-P450 reductase (POR) was also expressed in *E. coli* and purified as described.[11]

LC-MS analyses

A Thermo Q-Exactive HF instrument was set to full MS scan mode (positive ion) using a resolution setting of 120,000. The ESI probe spray voltage and capillary temperature were set to 5.0 kV and 300 °C, respectively. UPLC mobile phase A was 95% H_2O and 5% CH_3CN (v/v) with 10 mM NH_4OAc, and mobile phase B was 100% CH_3CN with 10 mM NH_4OAc (all v/v). Chromatographic separation was performed using an AQUITY UPLC® BEH C18 column (1.7 µm, 2.1 mm × 100 mm, P/N 186002352) equipped with an AQUITY UPLC® BEH C18

VanGuard™ pre-column (1.7 μm, 2.1 × 5 mm, P/N 186003975) (Waters) at a flow rate of 0.2 mL min^{-1} using gradient elution as follows: 2 to 100% B from 0 to 4 min, 100% B for 0.5 min, 100 to 2% B over 1.3 min, then 2% B for 2.2 min (8 min in total, all v/v). Data were processed using QualBrowser version 2.0.7 software.

*Preparation of diazo reagent (**5**)*

The diazo reagent was prepared immediately prior to the derivatization process according to a reported procedure,[5] with slight modification. Briefly, filtered Et_2O (0.5 mL, prepared as described above) was added to the nitrosourea (~3 mg) in a 2 mL amber vial followed by the addition of 0.5 mL of aqueous KOH (30% w/v). The contents were transferred to an 8 mL amber vial, and this process was repeated (3×) and the contents were combined (2 mL of Et_2O layer in total). The organic layer was extracted, dried with $MgSO_4$, and filtered through a cotton-plugged Pasteur pipette. The resulting solution was used for derivatization.

Enzyme incubations under an $^{18}O_2$ atmosphere (^{18}O experiment)

A reconstituted enzyme system consisting of 50 mM potassium phosphate buffer (pH 7.4) with P450 2B4 (0.2–0.5 μM), NADPH-P450 reductase (POR) (0.4–1.0 μM), P450 17A1 (0.2 μM), L-α-1,2-didodecanoyl (dilauroyl)-*sn*-glycero-3-phosphocholine (DLPC, 30 μg mL^{-1}, i.e. 45 μM), and progesterone (50 μM, substrate of P450 17A1) was added to a Thunberg tube. An NADPH-generating system consisting of 0.5 mM $NADP^+$, 10 mM glucose 6-phosphate, and 2.0 μg mL^{-1} yeast glucose 6-phosphate dehydrogenase[12] was placed in the cap of the Thunberg tube. The joint was sealed with Dow Corning High Vacuum Grease (Inland Vacuum Industries, Churchville, NY) and wrapped with parafilm to prevent leaks. The tube was evacuated and backfilled with argon gas

(purified through an in-line Resteck Oxygen Scrubber cartridge) and this cycle was repeated 10 times, using a gas train with a glass manifold.[13,14] After placing the tube under vacuum, the sidearm was flushed with argon gas and capped with a septum cap, $^{18}O_2$ gas was injected (from a lecture bottle equipped with a 2-stage regulator) for ~150 s followed by the injection of deuterated aldehydes (**1**, 100 μM; **2**, 500 μM; **3**, 1.0 mM). The Thunberg tube was preincubated at 37 °C for 3 min before initiating the reaction by mixing with the NADPH-generating system (by inversion). The reaction mixture (1.0 mL in total) was incubated at 37 °C for 15 min (for **2**) or 30 min (for **1** and **3**) before being quenched by the addition of ^{t}BME (3.0 mL) and mixed with a vortex device. HCl (3 M, 50 μL) was added to acidify the aqueous layer (to pH ~2) and mixed with a vortex device, and the organic layer (2.5 mL) was removed to a fresh vial. The extraction with ^{t}BME was repeated. The combined organic solvent (5.0 mL in total) was mixed with CH_3OH (500 μL) and diazo reagent in Et_2O (~200 μL), and incubated at room temperature for 10 min. The solvent was dried under an N_2 stream, and the residue was redissolved in CH_3OH (200 μL) and 10 μL of the sample was injected into LC-MS.

Relative ^{18}O incorporation (%) in each Thunberg tube was calculated from the peak area ratios of the 17α-^{16}OH progesterone and 17α-^{18}OH progesterone yielded from progesterone by P450 17A1 (Figure S2, *E* and *F*). The % ^{18}O incorporation of formic acid and carboxylic acid was calculated from the peak area ratio of the derivatized ^{16}O and ^{18}O products divided by the % ^{18}O incorporation in each Thunberg tube. Major changes from our original procedure[5] were (i) the use of ^{t}BME to extract formic acid instead of CH_2Cl_2, (ii) omitting the drying step with $MgSO_4$, and (iii) the inclusion of 10% CH_3OH (v/v) in the derivatization step.[15-18] Surprisingly, extraction of formic acid from the aqueous reaction mixture was found to be relatively inefficient with CH_2Cl_2, and the formic acid recovery was increased from ~3% to 70%. While drying the organic extract

with $MgSO_4$ was performed previously based on the premise that the derivatization reaction was sensitive to H_2O, the derivatization of acid with diazo reagent was shown to be stimulated by both H_2O and CH_3OH at 10% (v/v).[15-18] The drying step routinely reduced formic acid recovery to <10%, so the step was omitted (modification 2). Finally, inclusion of CH_3OH (10%, v/v) in the derivatization mixture was found to increase the yield of formic acid recovered as the pyridyl ester from ~15% to ~85%, and accordingly, this was subsequently included in all derivatizations. Collectively, these modifications increased the recovery of the formate ester by three orders of magnitude.[18]

Enzyme incubations in $H_2{}^{18}O$

^{18}O-Citronellal-*d* (**1′**) was prepared by incubating **1** (2.5 mM) in $H_2{}^{18}O$ (~93% ^{18}O) at room temperature for 24 h. The ^{18}O incorporation in **1** was calculated to be 92% from the peak area ratio of **1** and **1′** (Figure S4).

A reconstituted enzyme system prepared in 50 mM potassium phosphate buffer (pH 7.4) with P450 2B4 (0.5 μM), POR (1.0 μM), DLPC (30 μg mL^{-1}, i.e. 45 μM), and **1′** (100 μM) was added to a 1 mL reaction vial capped with a Teflon seal and preincubated at 37 °C for 3 min. The reaction was initiated by the addition of NADPH (1 mM), and the reaction mixture (200 μL total, 90% ^{18}O) was incubated at 37 °C for 30 min before being quenched by the addition of 3 M HCl (10 μL). The reaction mixture was extracted 2× with ^{t}BME, and the combined organic layer (3.0 mL) was mixed with CH_3OH (300 μL). The extract was derivatized and subjected to LC-MS analysis as described above.

Kinetic solvent isotope effect assays

The assays were conducted in 0.5 mL volumes of various D_2O compositions (0, 20, 40, 60, and 80%, v/v) containing 50 mM potassium phosphate buffer (pH or pD 7.4[19]), P450 2B4 (0.1 μM), POR (0.2 μM), DLPC (30 μg mL^{-1}, i.e. 45 μM), **1** (100 μM), and an NADPH-generating system. The reaction mixture, except for the NADPH-generating system, was preincubated in a 4 mL reaction vial capped with a Teflon seal at 37 °C for 3 min, followed by 30 min of incubation at 37 °C after initiation (by the addition of the NADPH-generating system). After quenching the reaction with 3 M HCl (25 μL), the resulting mixture was extracted and derivatized, and the sample was analyzed with LC-MS as described above.

The derivatized enzymatic products (both formic acid and citronellic acid) were quantified from their peak areas from *m/z* 167.0925 (**6a**) and 290.2115 (**7a**) (with 5 ppm mass tolerance) using the external standard curve (Figure S5, *A* and *B*), and the turnover rate was expressed as nmol product formed min^{-1} (nmol P450)$^{-1}$.

*Enzyme incubations of **2** and **3** under air*

A reconstituted enzyme system consisting of 50 mM potassium phosphate buffer (pH 7.4) with P450 2B4 (0.5 μM), POR (1.0 μM), DLPC (30 μg mL^{-1}, i.e. 45 μM), and substrate (**2** or **3**, 500 μM) was preincubated at 37 °C for 3 min. The reaction was initiated by the addition of an NADPH-generating system (consisting of 0.5 mM $NADP^+$, 10 mM glucose 6-phosphate, and 2.0 μg mL^{-1} yeast glucose 6-phosphate dehydrogenase[12]), and the reaction mixture (200 μL in total) was incubated at 37 °C for 5, 10, 20, and 30 min before being quenched by the addition of an equal amount of ice-cold CH_3CN. The contents were transferred to a 1.5 mL centrifuge tube and mixed with a vortex device. The tubes were centrifuged at $10^4 \times g$ for 10 min to precipitate the protein,

and the supernatant was maintained at 4 °C and injected (20 μL) on an AQUITY UPLC® BEH C18 column (1.7 μm, 2.1 mm × 100 mm, P/N 186002352) equipped with an AQUITY UPLC® BEH C18 VanGuard™ pre-column (1.7 μm, 2.1 mm × 5 mm, P/N 186003975, Waters), which was held at 40 °C. UPLC mobile phase A was 95% H_2O and 5% CH_3CN (v/v) with 10 mM NH_4OAc, and mobile phase B was 100% CH_3CN. Chromatographic separation was performed at a flow rate of 0.2 mL min^{-1} using gradient elution as follows: 0% B from for 0.5 min, 0 to 95% B from 0.5 to 7.0 min, 95% B for 0.9 min, 95 to 0% B over 0.1 min, then 0% B for 2.0 min (10 min in total, all v/v). Products were detected at a wavelength of 258 nm (for carboxylic acid products) and 245 nm (olefin products).

*Incubation of **10** with P450 2B4*

A reconstituted enzyme system consisting of 50 mM potassium phosphate buffer (pH 7.4) with P450 2B4 (0.4 μM), POR (0.8 μM), DLPC (30 μg mL^{-1}, i.e. 45 μM), and an NADPH-generating system[12] was preincubated at 37 °C for 3 min. **10** (200 μM) was added to start the reaction, and 100 μL of reaction mixture was aliquoted after each time point and mixed with 100 μL of ice-cold CH_3CN. After mixing using a vortex device, the contents were centrifuged and the supernatant was subjected to the LC-UV analysis as described above, using UPLC mobile phase C (H_2O with 0.1 % (v/v) CF_3CO_2H) and mobile phase B. A gradient elution profile was as follows: 5% B from for 0.5 min, 5 to 95% B from 0.5 to 7.0 min, 95% B for 0.9 min, 95 to 5%B over 0.1 min, then 5% B for 2.0 min (10 min in total, all v/v). As a control, **10** (200 μM) was incubated in 50 mM potassium phosphate buffer (pH 7.4) for the same time.

Supplemental Tables and Figures

Table 1. List of identified peptides from proteomic analysis of purified P450 2B4. P450 2B4 was digested with trypsin (following treatment with dithiothreitol and iodoacetamide) and the fragments were analyzed in the Vanderbilt Mass Spectrometer Research Center. Numbering of the amino acids is based on the FASTA amino acid sequence (Fig. S1B).

Peptide	*z*	Observed *m/z*	Delta ppm	Modifications
59-EKYGDVFTVYLGSRPVVVLCGTDAIR-85	4	729.64	1.0	C (+57)
63-YGDVFTVYLGSR-73	2	688.85	-0.5	
63-YGDVFTVYLGSRPVVVLCGTDAIR-85	3	886.46	0.7	C (+57)
63-YGDVFTVYLGSRPVVVLCGTDAIREALVDQAEAFSGR-98	4	1008.51	-0.5	C (+57)
74-PVVVLCGTDAIR-85	2	650.36	0.1	C (+57)
86-EALVDQAEAFSGR-98	3	464.9	0.1	
101-IAVVDPIFQGYGVIFANGER-120	3	722.38	-1.1	
127-FSLATMRDFGMGKR-140	4	404.96	2.0	
141-SVEERIQEEAR-151	3	449.23	-1.0	
160-SKGALLDNTLLFHSITSNIICSIVFGK-186	3	983.54	0.8	C (+57)
162-GALLDNTLLFHSITSNIICSIVFGK-186	3	911.83	-0.1	C (+57)
162-GALLDNTLLFHSITSNIICSIVFGKR-187	3	963.86	0.1	C (+57)
162-GALLDNTLLFHSITSNIICSIVFGKRFDYKDPVFLR-197	5	835.05	0.8	C (+57)
187-RFDYKDPVFLR-197	3	485.93	2.2	
188-FDYKDPVFLR-197	3	433.9	0.1	
237-NLQEINTFIGQSVEK-251	2	860.45	2.3	
237-NLQEINTFIGQSVEKHR-253	4	504.02	2.4	
252-HRATLDPSNPR-262	3	421.89	-1.8	
254-ATLDPSNPR-262	2	485.75	-2.1	
263-DFIDVYLLR-271	2	577.32	0.2	
275-DKSDPSSEFHHQNLILTVLSLFFAGTETTSTTLR-308	4	948.98	0.5	
275-DKSDPSSEFHHQNLILTVLSLFFAGTETTSTTLRYGFLLMLK-316	5	952.9	-0.5	
277-SDPSSEFHHQNLILTVLSLFFAGTETTSTTLR-308	3	1183.93	0.5	
309-YGFLLMLK-316	2	500.78	-0.5	M (+16)
324-VQKEIEQVIGSHRPPALDDR-343	5	458.25	2.7	
324-VQKEIEQVIGSHRPPALDDRAK-345	6	415.23	-0.5	
327-EIEQVIGSHRPPALDDR-343	3	644.67	0.9	
327-EIEQVIGSHRPPALDDRAK-345	5	427.03	-0.1	
344-AKMPYTDAVIHEIQR-358	4	447.73	2.7	M (+16)
346-MPYTDAVIHEIQR-358	3	530.26	-1.4	M (+16)
359-LGDLIPFGVPHTVTK-373	3	531.97	1.2	
385-NTEVFPVLSSALHDPR-400	3	594.65	2.7	
422-RNEGFMPFSLGK-433	3	466.9	-3.5	M (+16)
422-RNEGFMPFSLGKR-434	3	513.6	3.4	
423-NEGFMPFSLGK-433	2	621.8	3.3	M (+16)
423-NEGFMPFSLGKR-434	3	466.9	2.3	M (+16)
434-RICLGEGIAR-443	3	382.21	-2.2	C (+57)
434-RICLGEGIARTELFLFFTTILQNFSIASPVPPEDIDLTPR-473	2	1137.61	0.7	C (+57)
435-ICLGEGIARTELFLFFTTILQNFSIASPVPPEDIDLTPR-473	3	1464.44	-1.1	C (+57)
444-TELFLFFTTILQNFSIASPVPPEDIDLTPR-473	3	1141.27	0.1	
474-ESGVGNVPPSYQIR-497	2	751.88	-2.2	

A

```
ATGGCTAAGAAAACGAGCTCTAAAGGTAAGCTCCCCCCGGGACCCTCCCCTCTGCCCGTCCTGGGGAACCTTCTGCAGATGGACAGGAAGGGCCTGCTCCGCTCCTTCCTGCGGCTCCGA
 M  A  K  K  T  S  S  K  G  K  L  P  P  G  P  S  P  L  P  V  L  G  N  L  L  Q  M  D  R  K  G  L  L  R  S  F  L  R  L  R

GAGAAATACGGGGACGTGTTCACGGTGTACCTGGGATCCAGACCCGTGGTCGTGCTGTGTGGGACGGATGCCATCCGCGAGGCCCTCGTGGACCAAGCGGAGGCCTTTTCTGGCAGGGGG
 E  K  Y  G  D  V  F  T  V  Y  L  G  S  R  P  V  V  V  L  C  G  T  D  A  I  R  E  A  L  V  D  Q  A  E  A  F  S  G  R  G

AAGATCGCCGTGGTGGATCCGATCTTCCAGGGATACGGAGTGATCTTTGCCAACGGGGAGCGCTGGCGGGCCCTTCGGAGATTCTCCCTGGCCACCATGCGGGACTTCGGCATGGGGAAG
 K  I  A  V  V  D  P  I  F  Q  G  Y  G  V  I  F  A  N  G  E  R  W  R  A  L  R  R  F  S  L  A  T  M  R  D  F  G  M  G  K

CGGAGCGTGGAGGAGCGCATTCAGGAGGAGGCCCGGTGTCTGGTGGAGGAGCTGCGGAAATCCAAGGGAGCCCTCCTGGACAACACCTTGCTGTTCCACTCAATCACCTCCAACATCATC
 R  S  V  E  E  R  I  Q  E  E  A  R  C  L  V  E  E  L  R  K  S  K  G  A  L  L  D  N  T  L  L  F  H  S  I  T  S  N  I  I

TGCTCCATTGTCTTTGGAAAACGCTTTGACTACAAGGACCCCGTGTTCCTGCGGCTGCTGGACTTGTTCTTCCAGTCCTTCTCCCTCATCAGCTCCTTCTCCAGCCAGGTGTTCGAGCTC
 C  S  I  V  F  G  K  R  F  D  Y  K  D  P  V  F  L  R  L  L  D  L  F  F  Q  S  F  S  L  I  S  S  F  S  S  Q  V  F  E  L

TTCTCGGGCTTCCTAAAGCACTTTCCTGGCACGCACAGGCAGATCTACAGGAACCTGCAGGAGATCAACACTTTCATCGGCCAGAGCGTAGAGAAGCACCGCGCAACCCTGGACCCCAGC
 F  S  G  F  L  K  H  F  P  G  T  H  R  Q  I  Y  R  N  L  Q  E  I  N  T  F  I  G  Q  S  V  E  K  H  R  A  T  L  D  P  S

AACCCCAGGGATTTCATCGACGTCTACCTGCTCCGCATGGAAAAAGACAAGTCCGACCCAAGCAGCGAGTTCCACCACCAGAACCTCATCCTCACCGTGCTCTCGCTCTTCTTCGCCGGC
 N  P  R  D  F  I  D  V  Y  L  L  R  M  E  K  D  K  S  D  P  S  S  E  F  H  H  Q  N  L  I  L  T  V  L  S  L  F  F  A  G

ACCGAGACCACCAGCACCACCCTCCGCTACGGCTTCCTGCTCATGCTCAAGTACCCACACGTCACAGAGAGAGTCCAGAAGGAGATTGAGCAGGTGATCGGCTCCCACCGCCCTCCGGCC
 T  E  T  T  S  T  T  L  R  Y  G  F  L  L  M  L  K  Y  P  H  V  T  E  R  V  Q  K  E  I  E  Q  V  I  G  S  H  R  P  P  A

CTCGATGACCGAGCCAAAATGCCCTACACGGACGCGGTCATCCACGAGATCCAGCGGCTCGGGGACCTCATCCCCTTCGGGGTGCCCCACACGGTCACAAAAGACACACAGTTCCGAGGC
 L  D  D  R  A  K  M  P  Y  T  D  A  V  I  H  E  I  Q  R  L  G  D  L  I  P  F  G  V  P  H  T  V  T  K  D  T  Q  F  R  G

TATGTCATCCCCAAGAACACGGAAGTGTTCCCCGTCCTGAGCTCGGCTCTCCATGACCCGCGCTACTTTGAAACACCGAACACCTTCAACCCCGGCCACTTTCTGGATGCCAACGGGGCA
 Y  V  I  P  K  N  T  E  V  F  P  V  L  S  S  A  L  H  D  P  R  Y  F  E  T  P  N  T  F  N  P  G  H  F  L  D  A  N  G  A

CTGAAGAGGAATGAAGGCTTTATGCCCTTCTCCCTGGGGAAGCGCATTTGTCTGGGCGAAGGCATCGCGCGGACCGAGCTGTTCCTCTTCTTCACCACCATCCTGCAGAACTTCTCCATC
 L  K  R  N  E  G  F  M  P  F  S  L  G  K  R  I  C  L  G  E  G  I  A  R  T  E  L  F  L  F  F  T  T  I  L  Q  N  F  S  I

GCCAGCCCCGTGCCTCCCGAGGACATCGACCTCACTCCCCGGGAGAGTGGCGTGGGCAACGTGCCCCCGAGCTACCAGATCCGCTTCCTGGCCCGCCACCACCACCACCAC
 A  S  P  V  P  P  E  D  I  D  L  T  P  R  E  S  G  V  G  N  V  P  P  S  Y  Q  I  R  F  L  A  R  H  H  H  H  H
```

B

```
FASTA      MEFSLLLLLAFLAGLLLLLFRGHPKAHGRLPPGPSPLPVLGNLLQMDRKGLLRSFLRLRE   60
2B4        -------------------MAKKTSSKGKLPPGPSPLPVLGNLLQMDRKGLLRSFLRLRE   41

FASTA      KYGDVFTVYLGSRPVVVLCGTDAIREALVDQAEAFSGRGKIAVVDPIFQGYGVIFANGER   120
2B4        KYGDVFTVYLGSRPVVVLCGTDAIREALVDQAEAFSGRGKIAVVDPIFQGYGVIFANGER   101

FASTA      WRALRRFSLATMRDFGMGKRSVEERIQEEARCLVEELRKSKGALLDNTLLFHSITSNIIC   180
2B4        WRALRRFSLATMRDFGMGKRSVEERIQEEARCLVEELRKSKGALLDNTLLFHSITSNIIC   161

FASTA      SIVFGKRFDYKDPVFLRLLDLFFQSFSLISSFSSQVFELFPGFLKHFPGTHRQIYRNLQE   240
2B4        SIVFGKRFDYKDPVFLRLLDLFFQSFSLISSFSSQVFELF SGFLKHFPGTHRQIYRNLQE  221

FASTA      INTFIGQSVEKHRATLDPSNPRDFIDVYLLRMEKDKSDPSSEFHHQNLILTVLSLFFAGT   300
2B4        INTFIGQSVEKHRATLDPSNPRDFIDVYLLRMEKDKSDPSSEFHHQNLILTVLSLFFAGT   281

FASTA      ETTSTTLRYGFLLMLKYPHVTERVQKEIEQVIGSHRPPALDDRAKMPYTDAVIHEIQRLG   360
2B4        ETTSTTLRYGFLLMLKYPHVTERVQKEIEQVIGSHRPPALDDRAKMPYTDAVIHEIQRLG   341

FASTA      DLIPFGVPHTVTKDTQFRGYVIPKNTEVFPVLSSALHDPRYFETPNTFNPGHFLDANGAL   420
2B4        DLIPFGVPHTVTKDTQFRGYVIPKNTEVFPVLSSALHDPRYFETPNTFNPGHFLDANGAL   401

FASTA      KRNEGFMPFSLGKRICLGEGIARTELFLFFTTILQNFSIASPVPPEDIDLTPRESGVGNV   480
2B4        KRNEGFMPFSLGKRICLGEGIARTELFLFFTTILQNFSIASPVPPEDIDLTPRESGVGNV   461

FASTA      PPSYQIRFLAR-----   491
2B4        PPSYQIRFLARHHHHH   477
```

Figure S1. P450 2B4 cDNA nucleotide (A) and predicted amino acid (B) sequences. The coverage of analyzed peptides (Table S1) is indicated in Part B with yellow shading. The amino acid sequence is identical to that of Scott et al.[20]

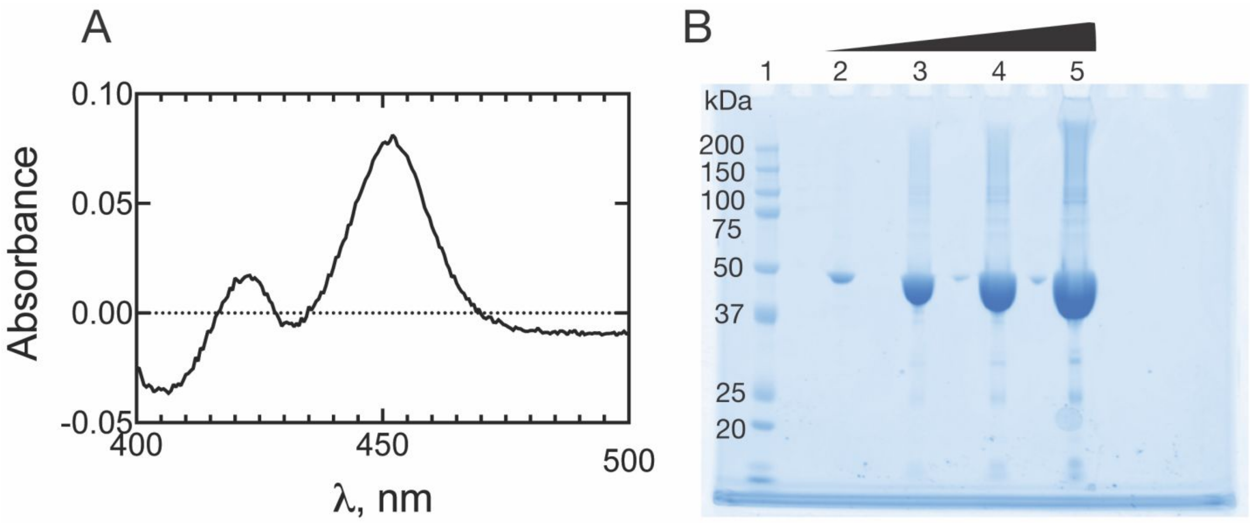

Figure S2. Reduced-CO vs. reduced difference spectrum and SDS-gel electrophoresis of purified P450 2B4. *A*, Reduced-CO vs. reduced difference spectrum of P450 2B4. The spectrum was recorded according to a reported procedure.[12,21] *B*, SDS gel electrophoretogram of purified P450 2B4. Lane 1, protein ladder (Precision Plus Protein Kaleidoscope, Bio-Rad); Lanes 2–5, increasing amounts of P450 2B4 (10, 50, 100, and 200 pmol). Proteins were separated on a NuPAGE 10% Bis-Tris gel with MOPS running buffer and then stained with SimplyBlue SafeStain (Thermo Fisher Scientific). The masses of the standard markers are shown on the left.

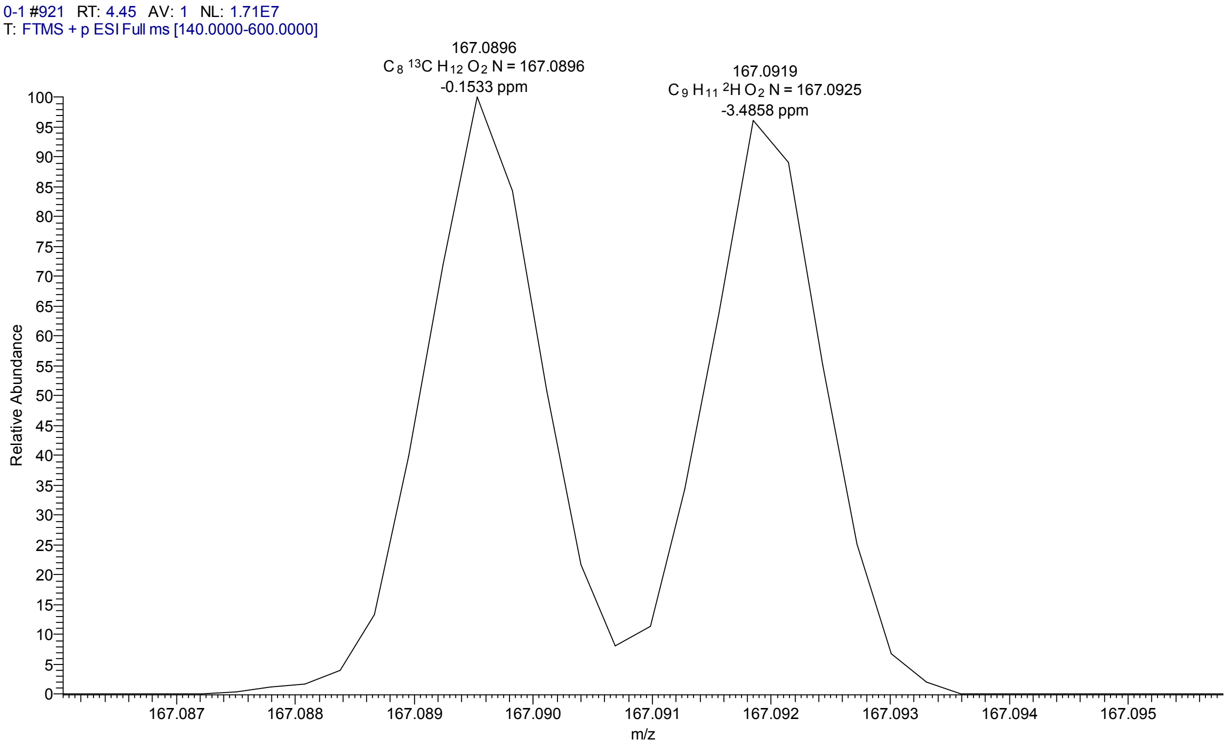

Figure S3. Mass spectra of 3-(pyridin-3-yl)propyl esters of $H^{13}COOH$ (*m/z* 167.0896) and DCOOH (*m/z* 167.0925). Each peak is labeled with the observed *m/z* value, chemical formula with theoretical mass, and deviation (ppm) between theoretical and observed mass (from top to bottom).

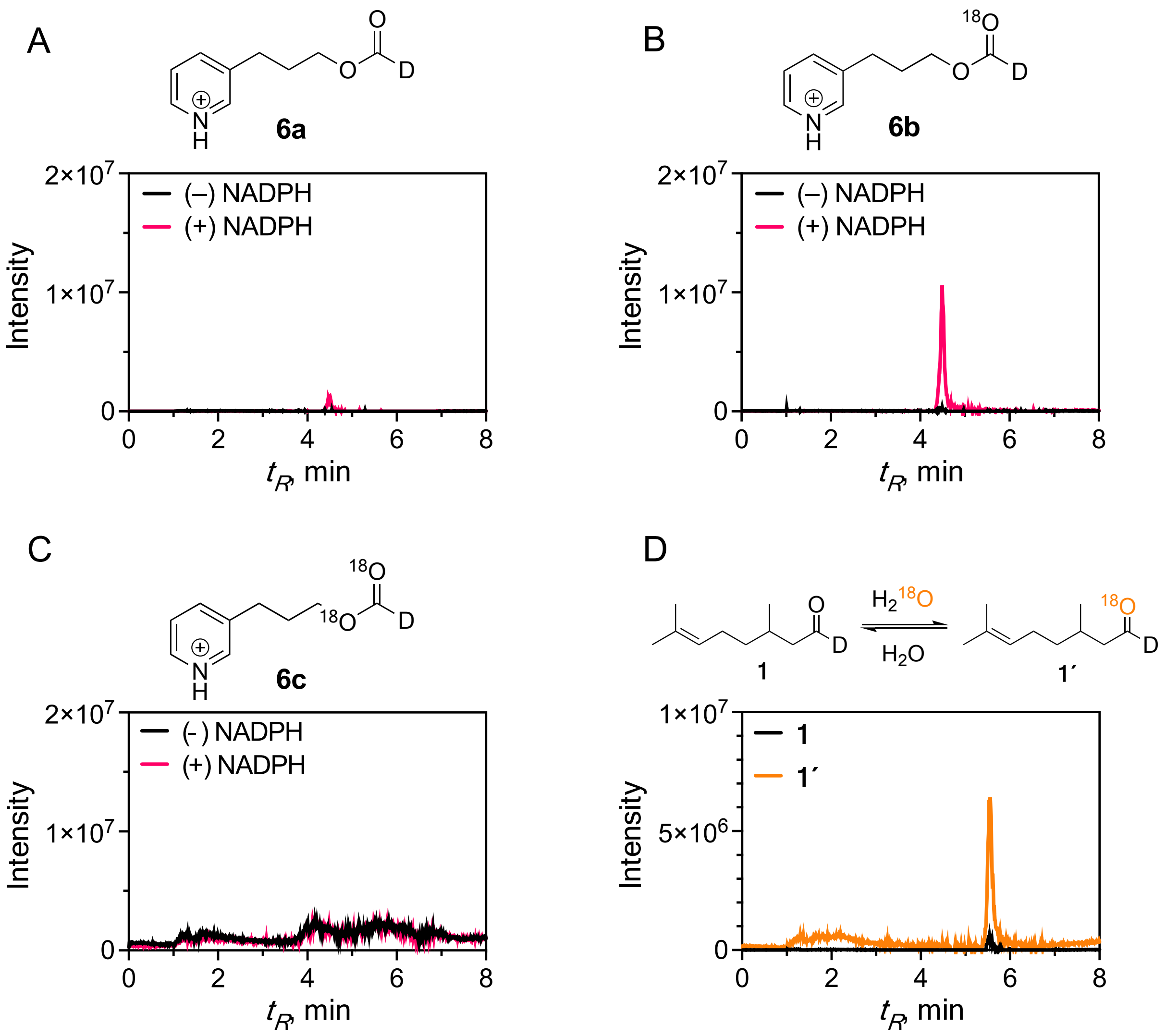

Figure S4. P450 2B4 incubation with **1** in $H_2{}^{18}O$. *A–C*, representative mass traces of **6a** (*m/z* 167.0925) (A), **6b** (*m/z* 169.0968) (B), and **6c** (*m/z* 171.1010) (C) with 5 ppm mass tolerance. Each trace shows peaks with (–, red line) or without (–, black line) the NADPH-generating system at the time of incubation; *D*, representative mass trace of *m/z* 155.1420 (–, black line, **1**) and 157.1463 (–, orange line, **1´**) with 5 ppm mass tolerance. The calculated percentage of ^{18}O incorporation in citronellal-*d* was 92%.

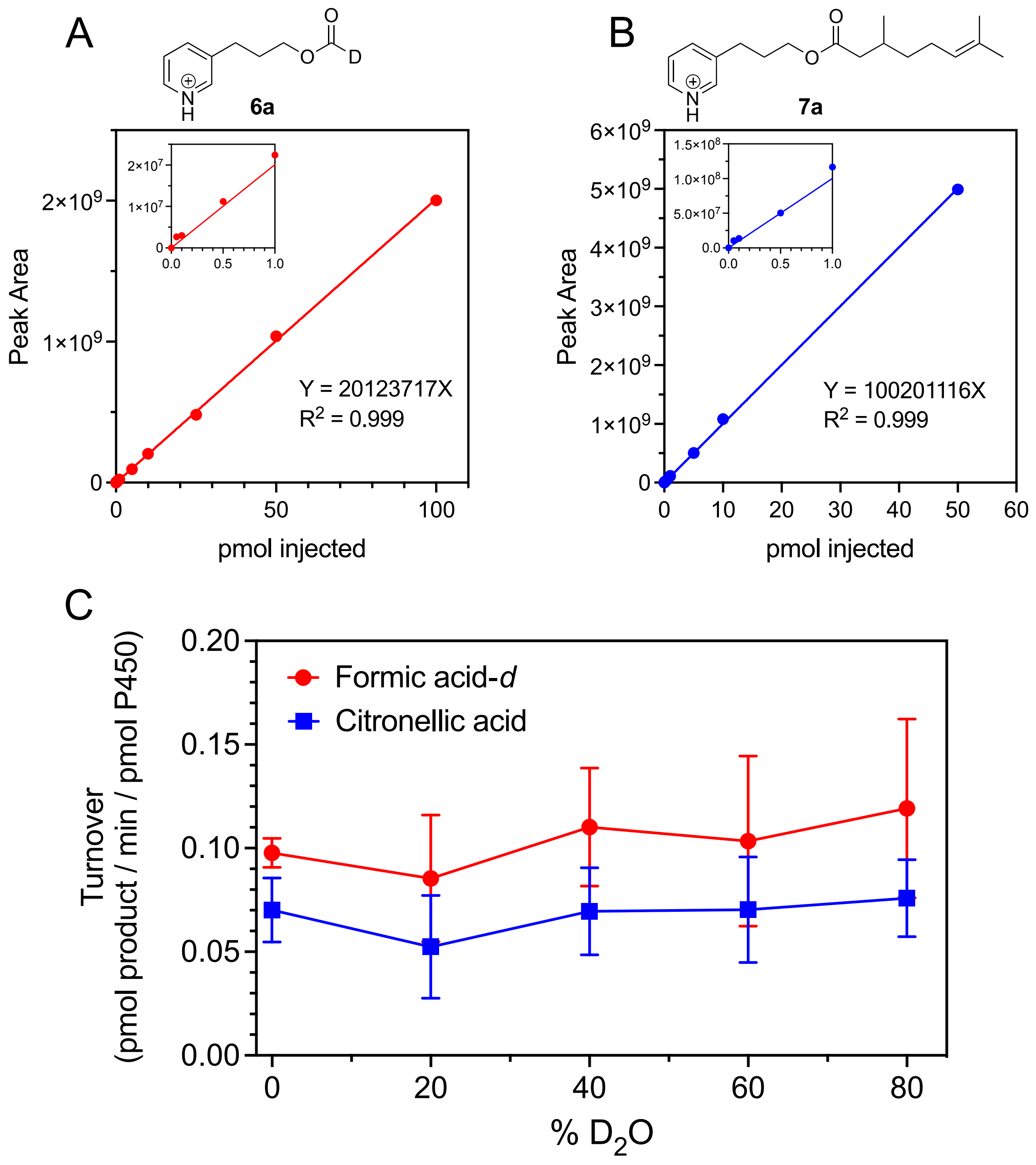

Figure S5. Kinetic solvent isotope effects. *A*, *B*, Standard curves for synthesized **6a** (A) and **7a** (B); *C*, kinetic solvent isotope effects on deformylation (–, red line) and formation of the carboxylic acid derivative (–, blue line) of **1** by P450 2B4. Each product (formic acid-*d* and citronellic acid) was derivatized and detected as a 3-(pyridin-3-yl)propyl ester, then quantified using the above standard curves. The points are means ± SD from triplicate assays.

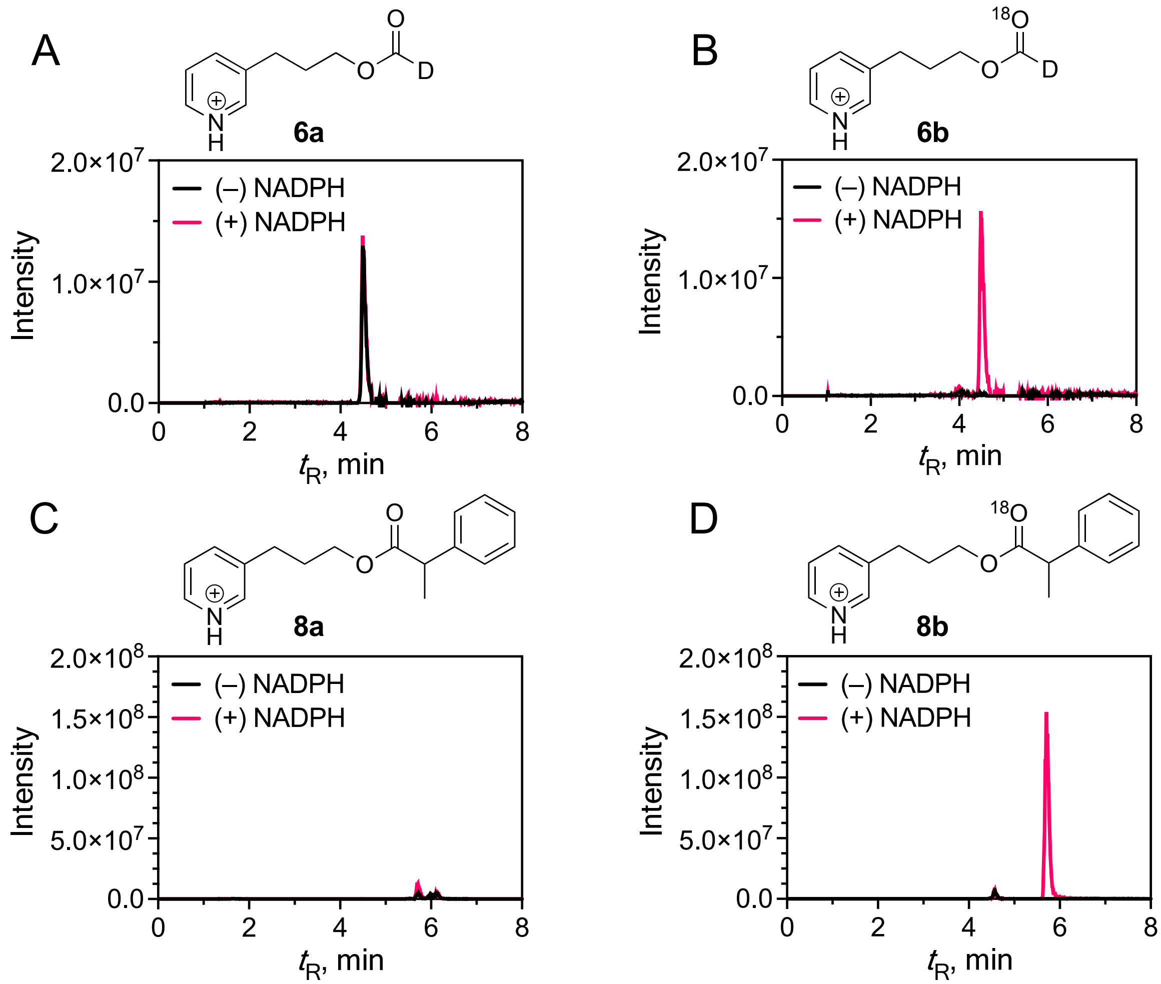

Figure S6. P450 2B4 incubation with **2** under an $^{18}O_2$ atmosphere. Representative mass traces of **6a** (*m/z* 167.0925) (A), **6b** (*m/z* 169.0968) (B), **8a** (*m/z* 270.1489) (C), and **8b** (*m/z* 272.1531) (D) with 5 ppm mass tolerance. Each trace shows peaks with (–, red line) or without (–, black line) the NADPH-generating system in the incubation. The calculated amount of non-enzymatically produced formic acid-*d* (detected as a pyridyl ester **6a**) was ~100 pmol, which corresponds to ~0.02% of **2** in the reaction mixture.

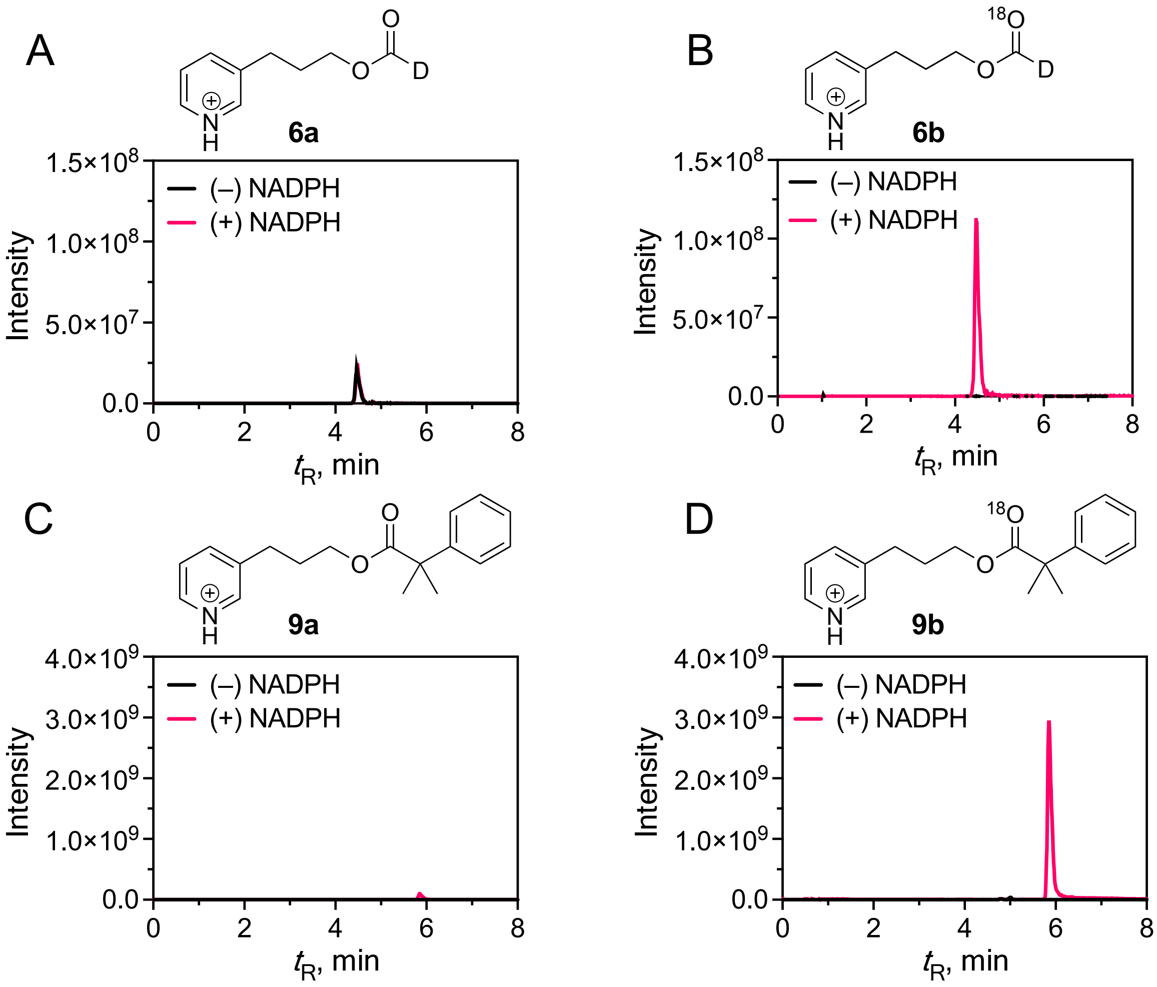

Figure S7. P450 2B4 incubation with **3** under an $^{18}O_2$ atmosphere. Representative mass traces of **6a** (*m/z* 167.0925) (A), **6b** (*m/z* 169.0968) (B), **9a** (*m/z* 284.1645) (C), and **9b** (*m/z* 286.1688) (D) with 5 ppm mass tolerance. Each trace shows peaks with (–, red line) or without (–, black line) the NADPH-generating system in the incubation. The calculated amount of non-enzymatically produced formic acid-*d* (detected as a pyridyl ester **6a**) was ~200 pmol, which corresponds to ~0.02% of **3** in the reaction mixture.

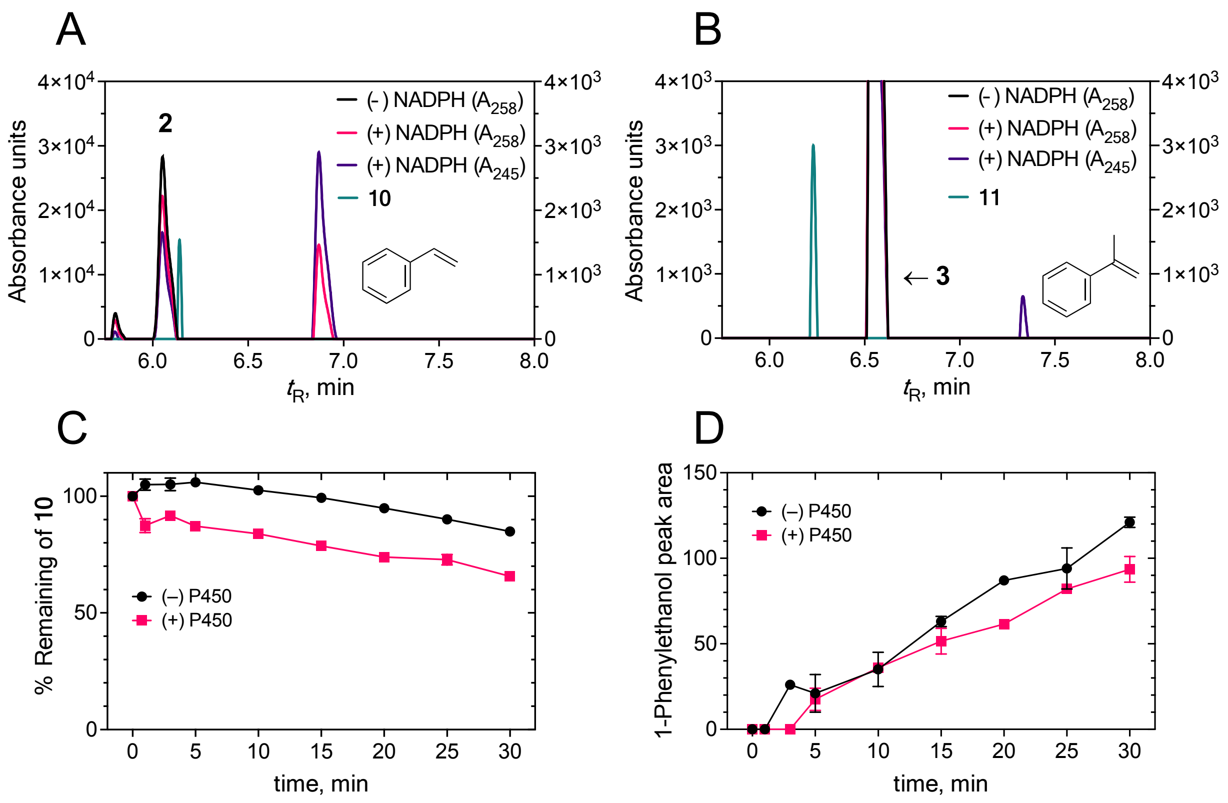

Figure S8. Search for a Baeyer-Villiger intermediate. *A* and *B*, P450 2B4 (0.5 μM) incubation of **2** (A) or **3** (B). Chromatograms show absorbance with (A_{258}, –, red line, left y-axis; A_{245}, –, purple line, left y-axis) or without (A_{258}, –, black line, left y-axis) the NADPH-generating system during the time of incubation. Synthetic authentic standards of possible Baeyer-Villiger intermediates (**10** and **11**, A_{258}, –, blue line, right y-axis) are included in each panel. *C* and *D*, P450 2B4 (0.4 μM) incubation of **10**. The graphs show the percentage of remaining **10** (t_R 6.07 min, C) or peak area of 1-phenylethanol (t_R 4.71 min, D) after incubating with (–, red line) or without (–, black line) reconstituted P450 enzymes. Each time point is the mean ± range of duplicate assays.

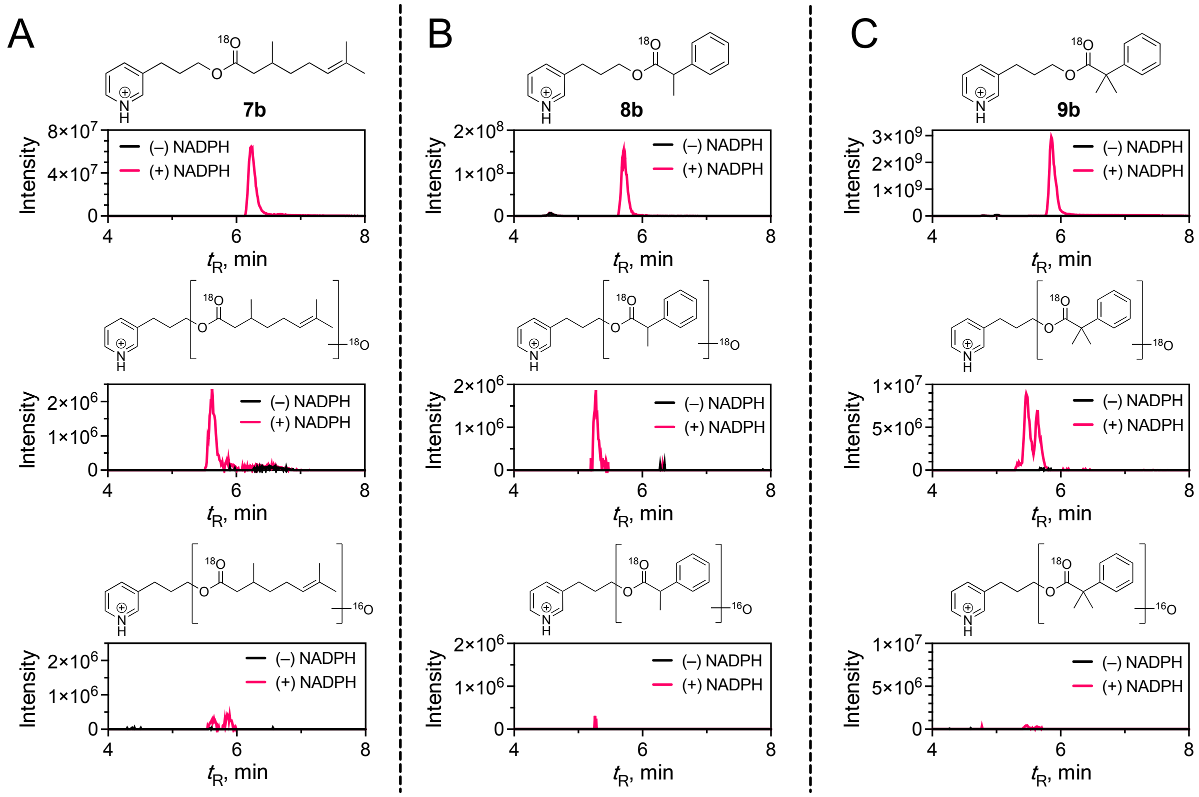

Figure S9. Search for additional metabolites from $^{18}O_2$ experiment data. Representative mass traces of mono-oxygenated carboxylic acid products of **1** (A), **2** (B), and **3** (C) are shown. Top row, carboxylic acid products, showing the same trace from Figures 1D, S6D, and S7D; middle and bottom rows, mono-^{18}O (middle) or ^{16}O (bottom) incorporated carboxylic acid products. Note that these products are eluting slightly before carboxylic acid products (**7b**, **8b**, and **9b**) and have 50- to 100-fold weaker peak intensity. Oxygenated positions of these products were not determined. Exact masses of these metabolites were: *A*, *m/z* 292.2157 (top), 310.2149 (middle), and 308.2106 (bottom); *B*, *m/z* 272.1531 (top), 290.1523 (middle), and 288.1480 (bottom); *C*, *m/z* 286.1688 (top), 304.1679 (middle) and 302.1637 (bottom). All mass traces are with 5 ppm mass tolerance and each trace shows peaks with (–, red line) or without (–, black line) an NADPH-generating system in the incubation. Traces between t_R 4 and 8 min are presented to emphasize the difference of retention time, and no peaks were detected before 4 min.

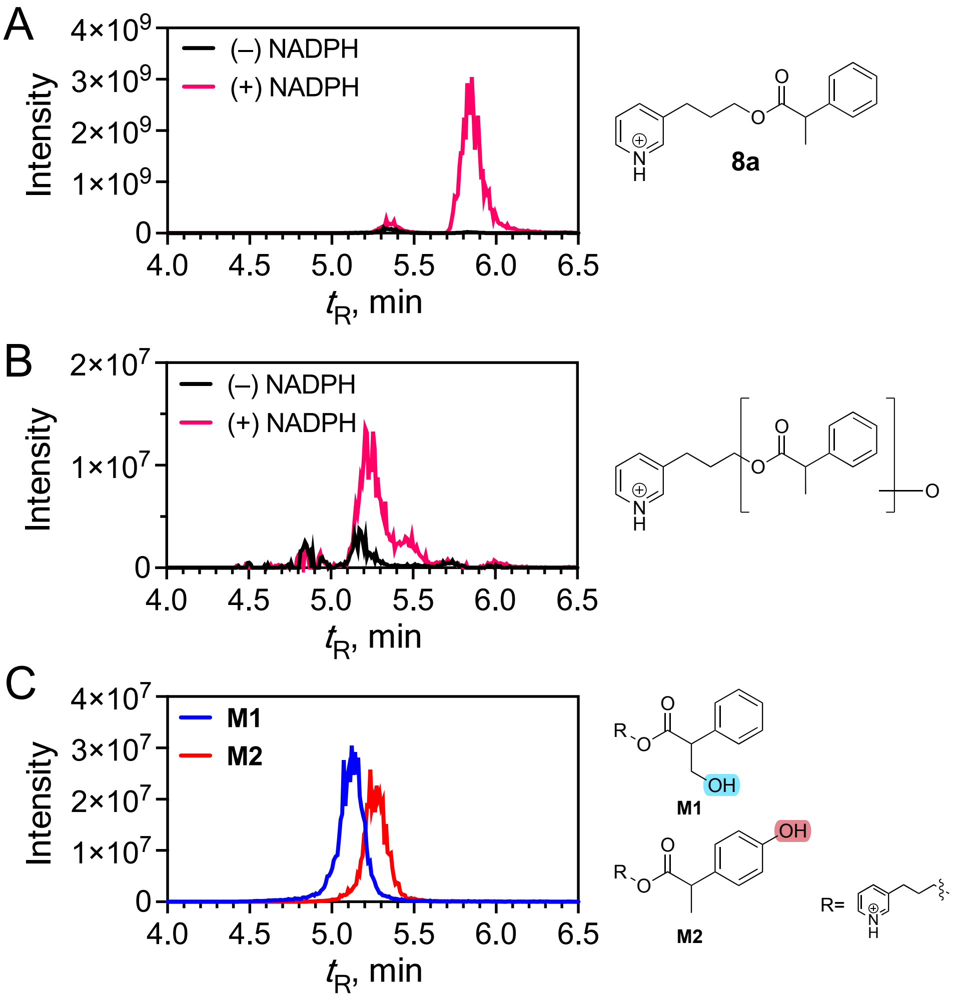

Figure S10. LC-MS analysis of other metabolites of **2**. *A* and *B*, representative mass traces of **8a** (*m/z* 270.1489, A), and mono-oxygenated carboxylic acid products (*m/z* 286.1438, B) from P450 2B4 incubation, which were derivatized after the incubation as described above and detected as 3-(pyridin-3-yl)propyl esters. Each trace shows peaks with (–, red line) or without (–, black line) the NADPH-generating system in the incubation; *C*, synthesized standards of mono-oxygenated carboxylic acid products (*m/z* 286.1438, **M1**; t_R=5.13 min, **M2**; t_R=5.25 min). All mass traces are with 5 ppm mass tolerance and traces between 4 and 6.5 min are presented to emphasize the difference of retention time.

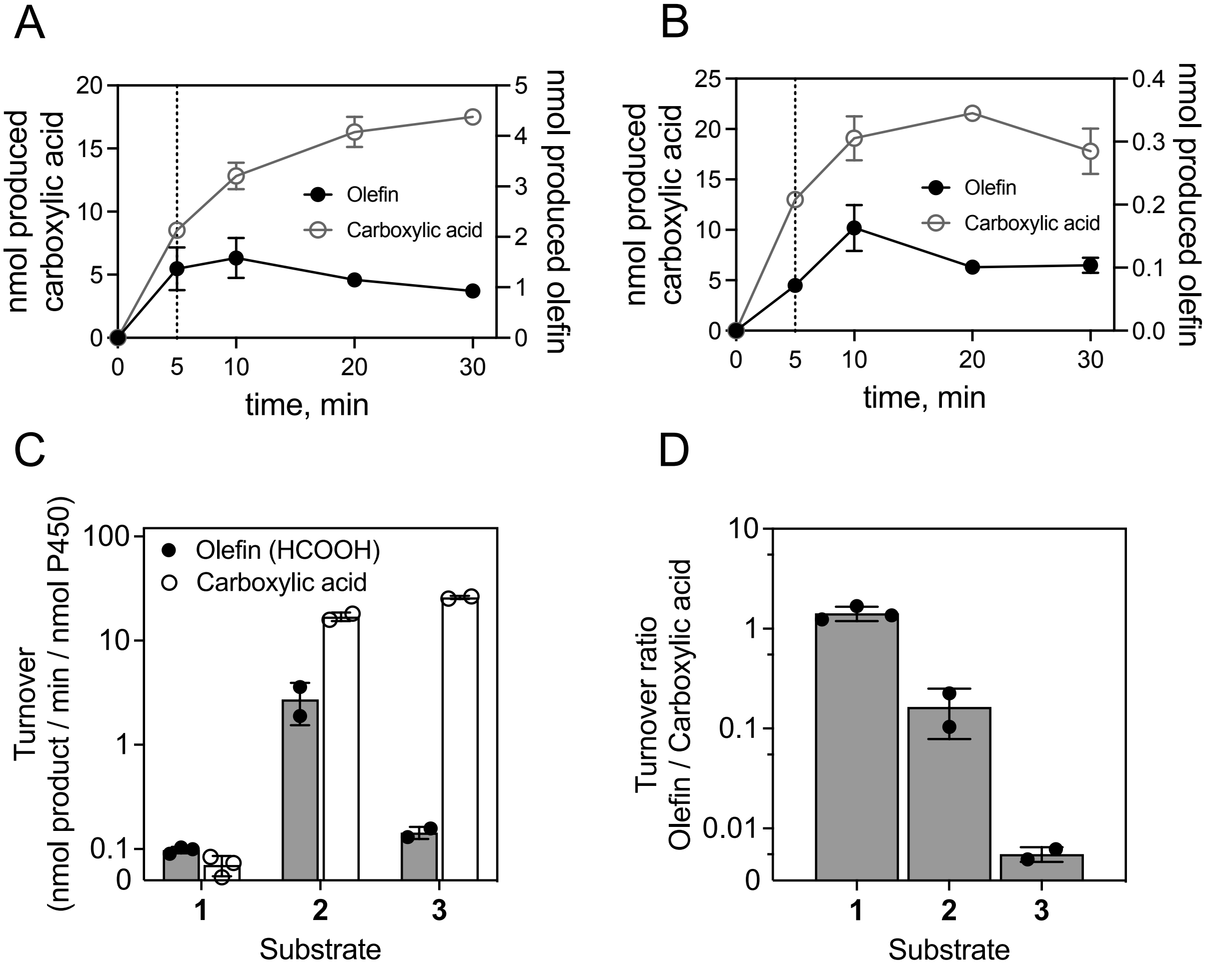

Figure S11. Comparison of deformylation vs carboxylic acid formation. *A* and *B*, time dependent product formation from **2** (A) and **3** (B). Data points represent mean ± range of duplicate assays; *C* and *D*, Turnover rates. Y-axis is presented as log scale in both panels. Turnover rates of **1** are the same as presented in Figure S5 (0% D_2O), and rates of **2** and **3** are calculated from 5 min incubation time data (indicated as a dotted line in panels A and B). Each graph represent mean ± SD of triplicate (**1**) or mean ± range of duplicate (**2**, **3**) assays.

^{1}H NMR ($CDCl_3$ with 0.03 v/v% TMS, 600 MHz)

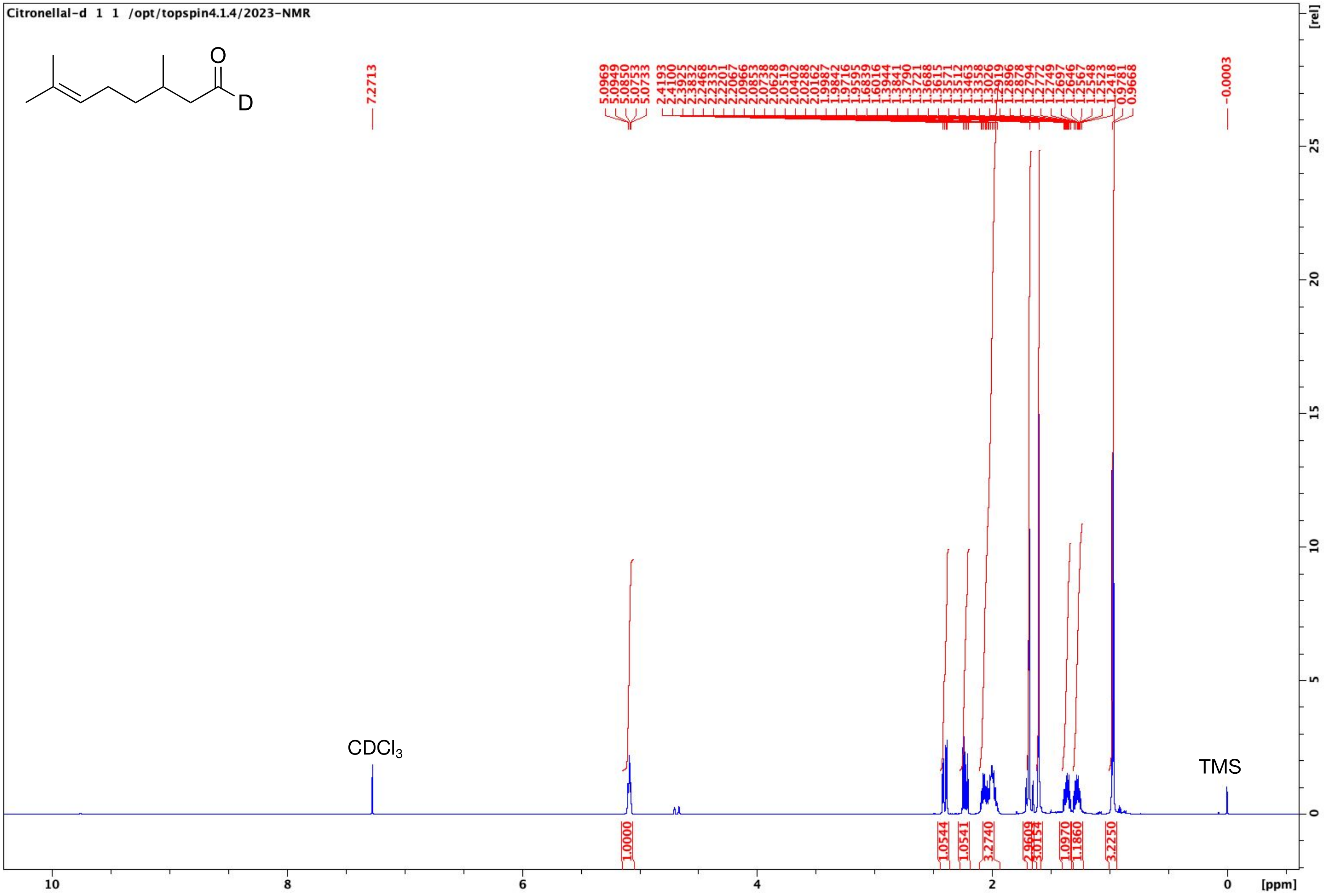

^{13}C NMR ($CDCl_3$ with 0.03 v/v% TMS, 150 MHz)

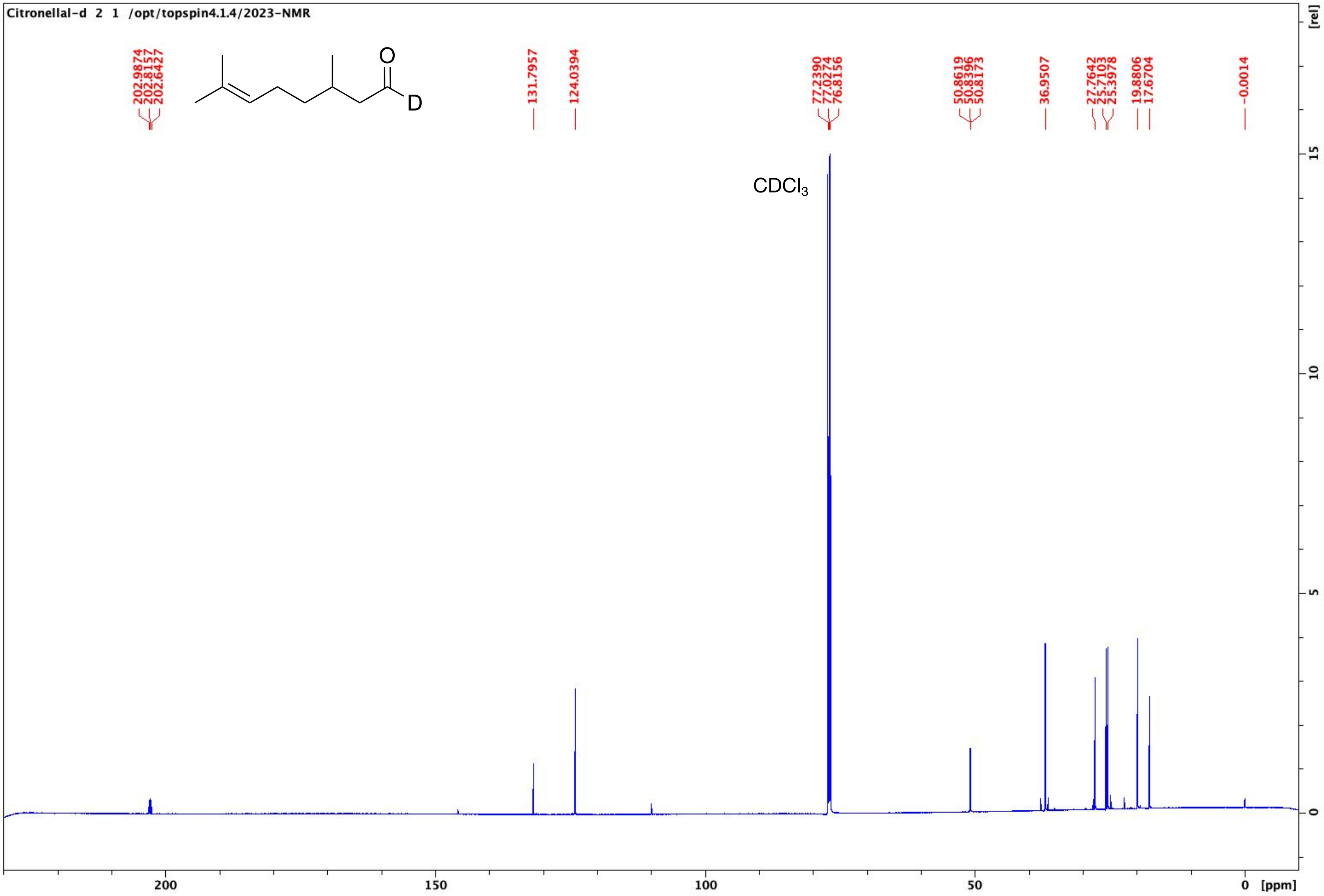

^{1}H NMR ($CDCl_3$ with 0.03 v/v% TMS, 600 MHz)

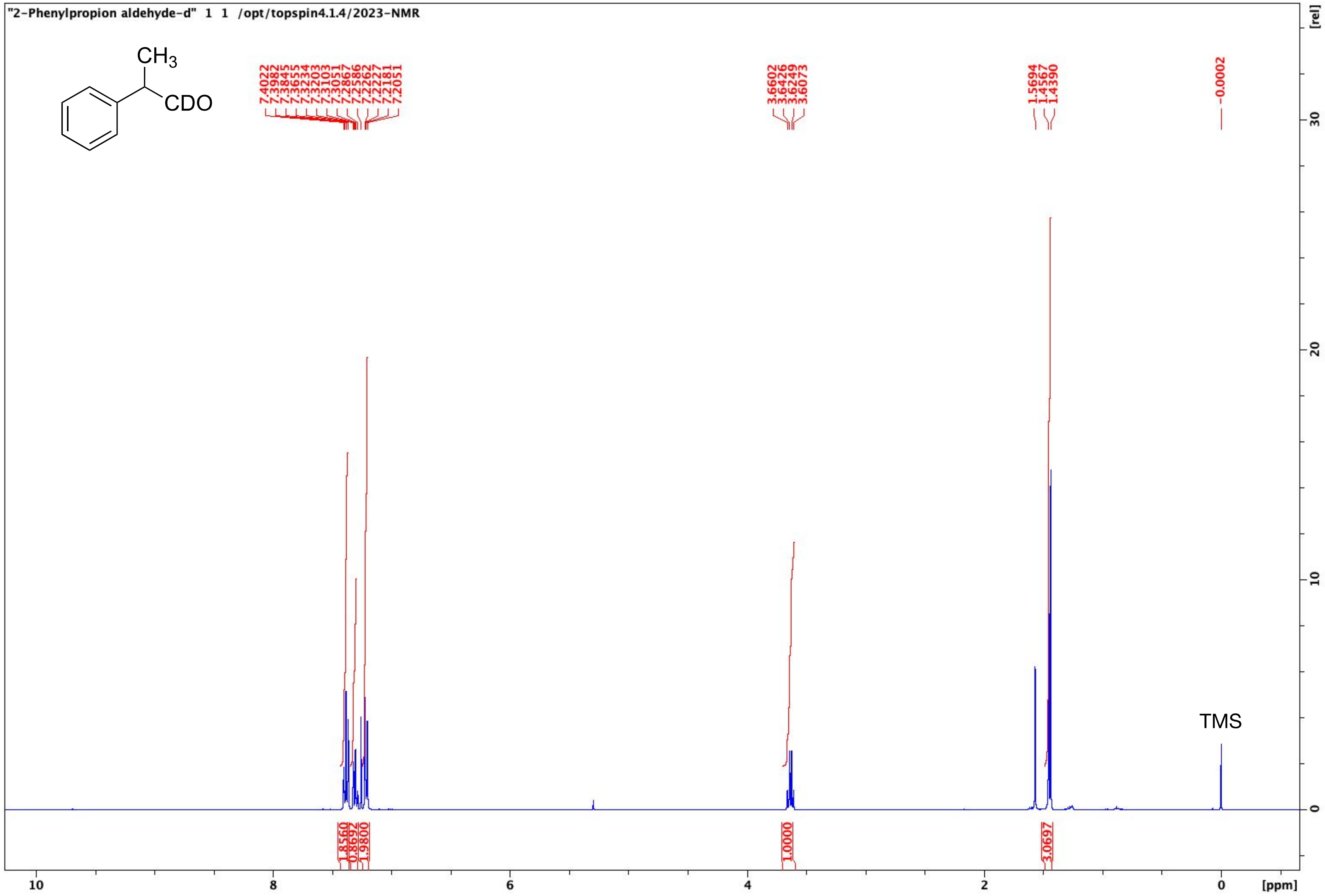

^{13}C NMR ($CDCl_3$ with 0.03 v/v% TMS, 150 MHz)

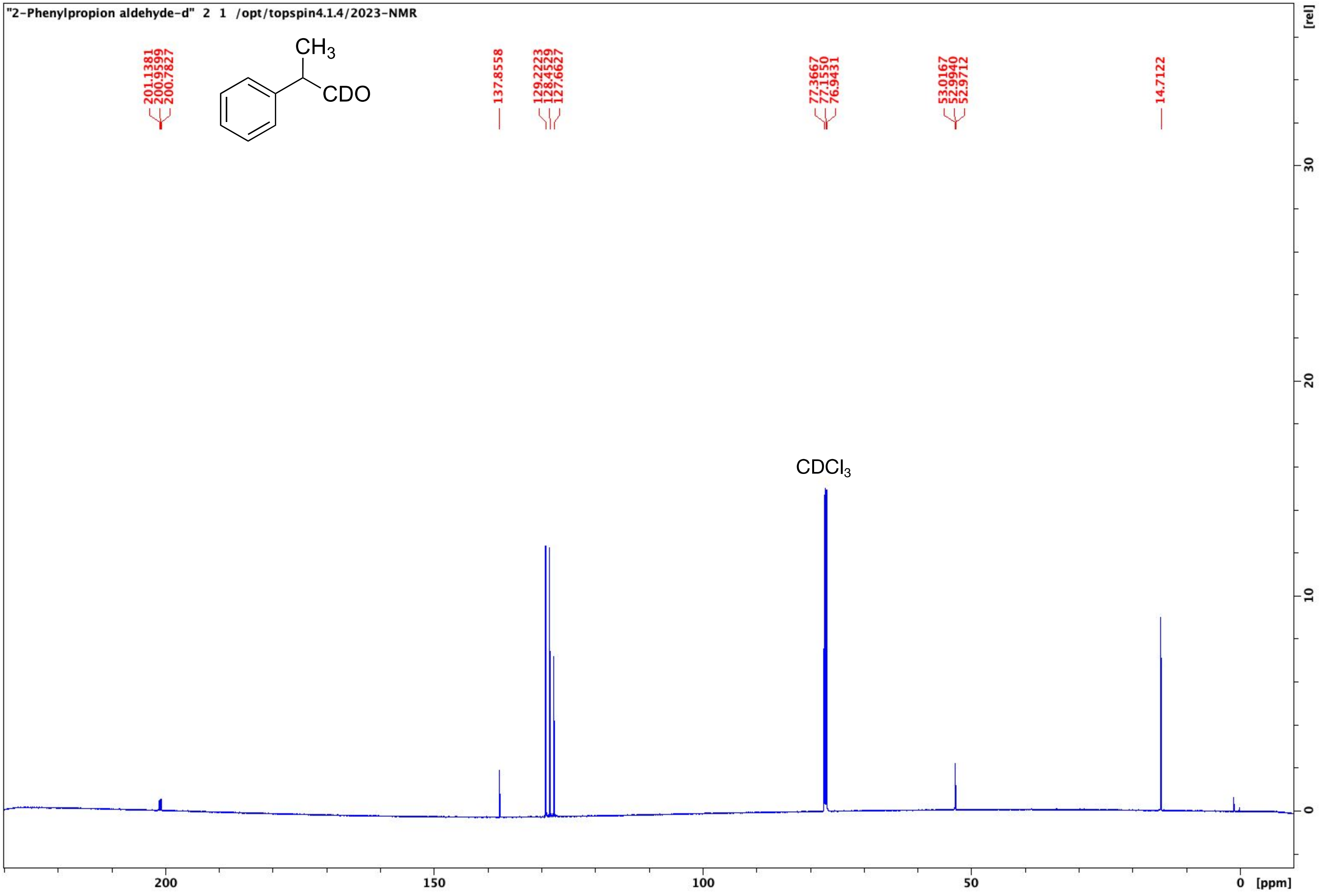

¹H NMR ($CDCl_3$ with 0.03 v/v% TMS, 600 MHz)

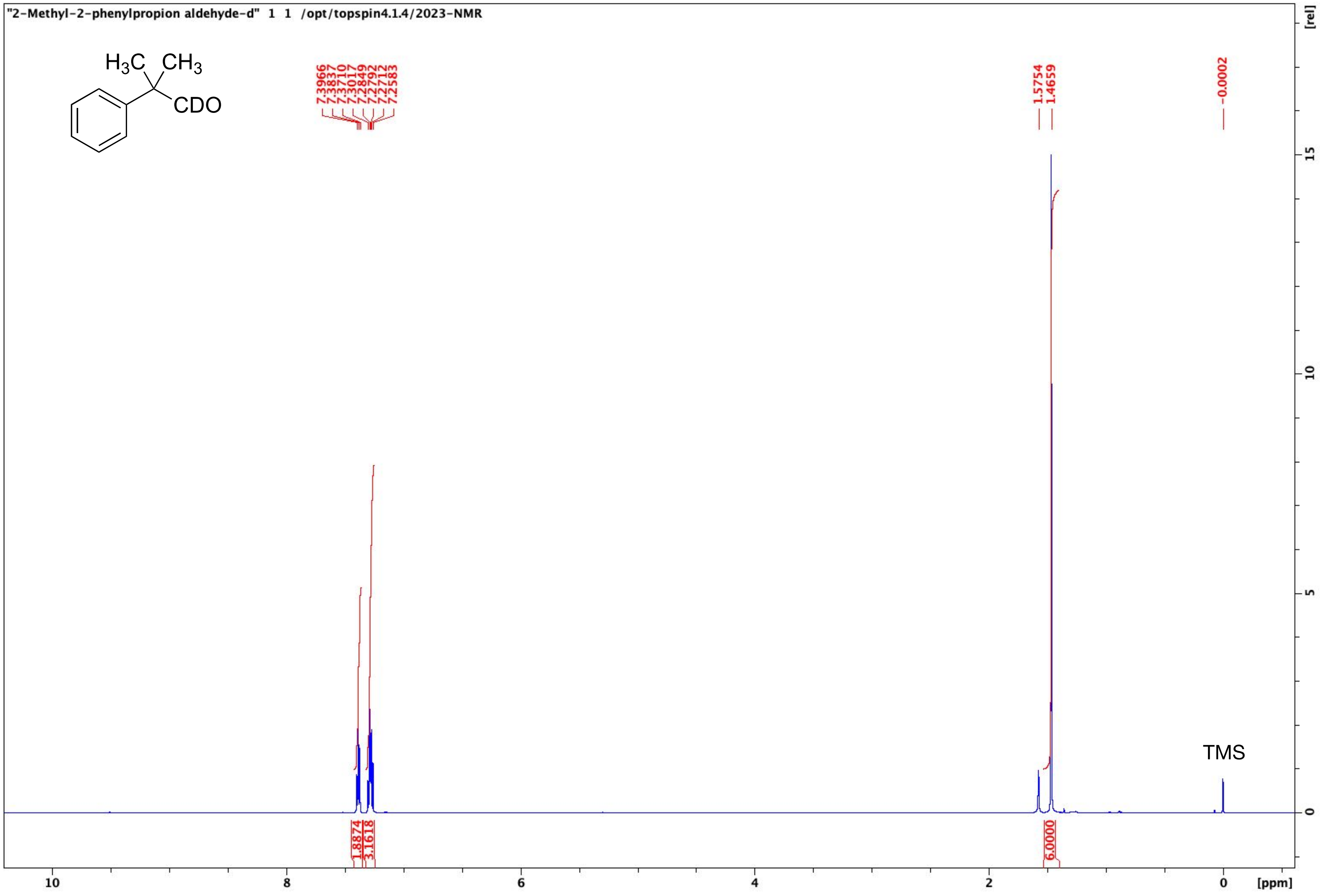

^{13}C NMR ($CDCl_3$ with 0.03 v/v% TMS, 150 MHz)

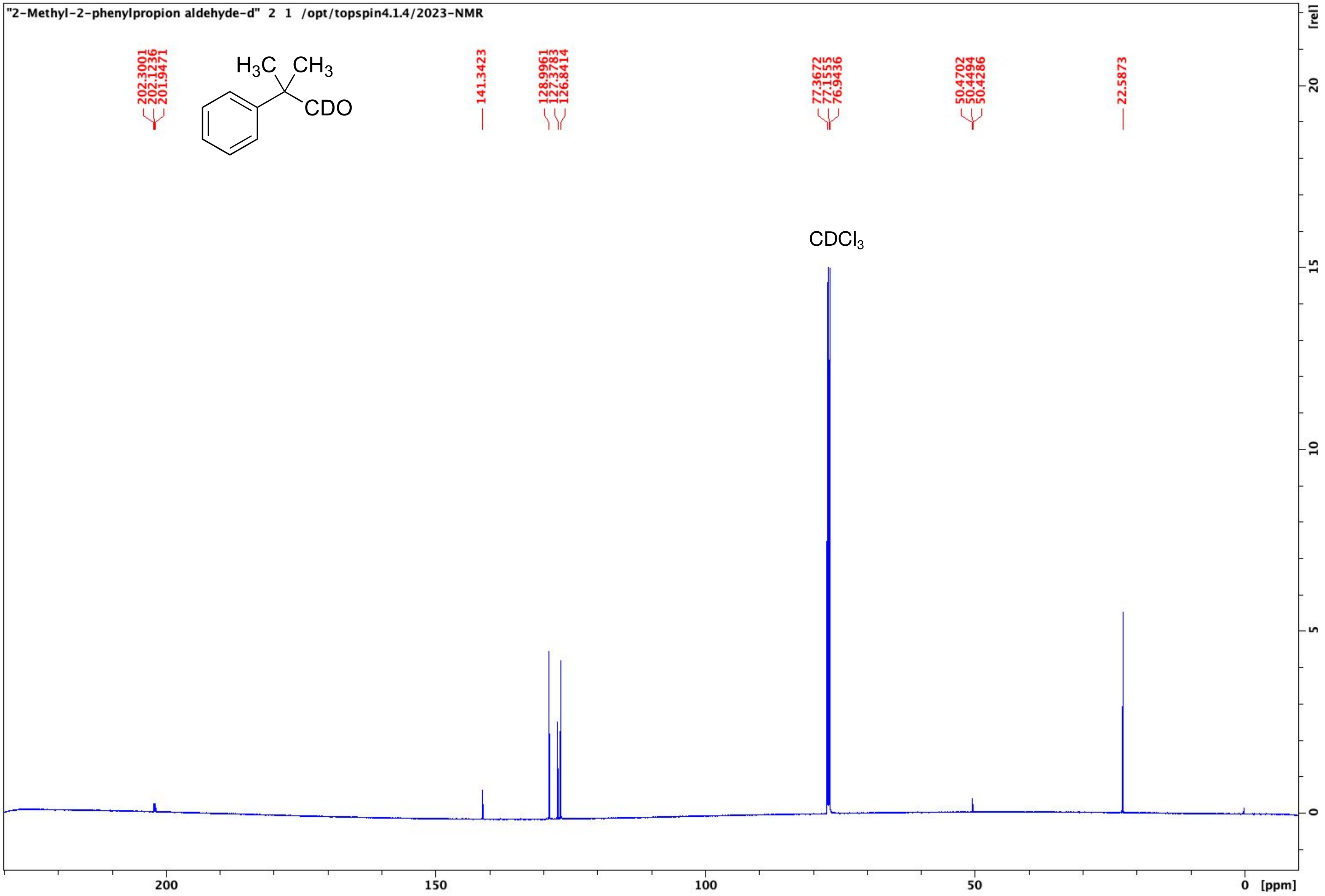

^{1}H NMR (CDCl$_3$ with 0.03 v/v% TMS, 600 MHz)

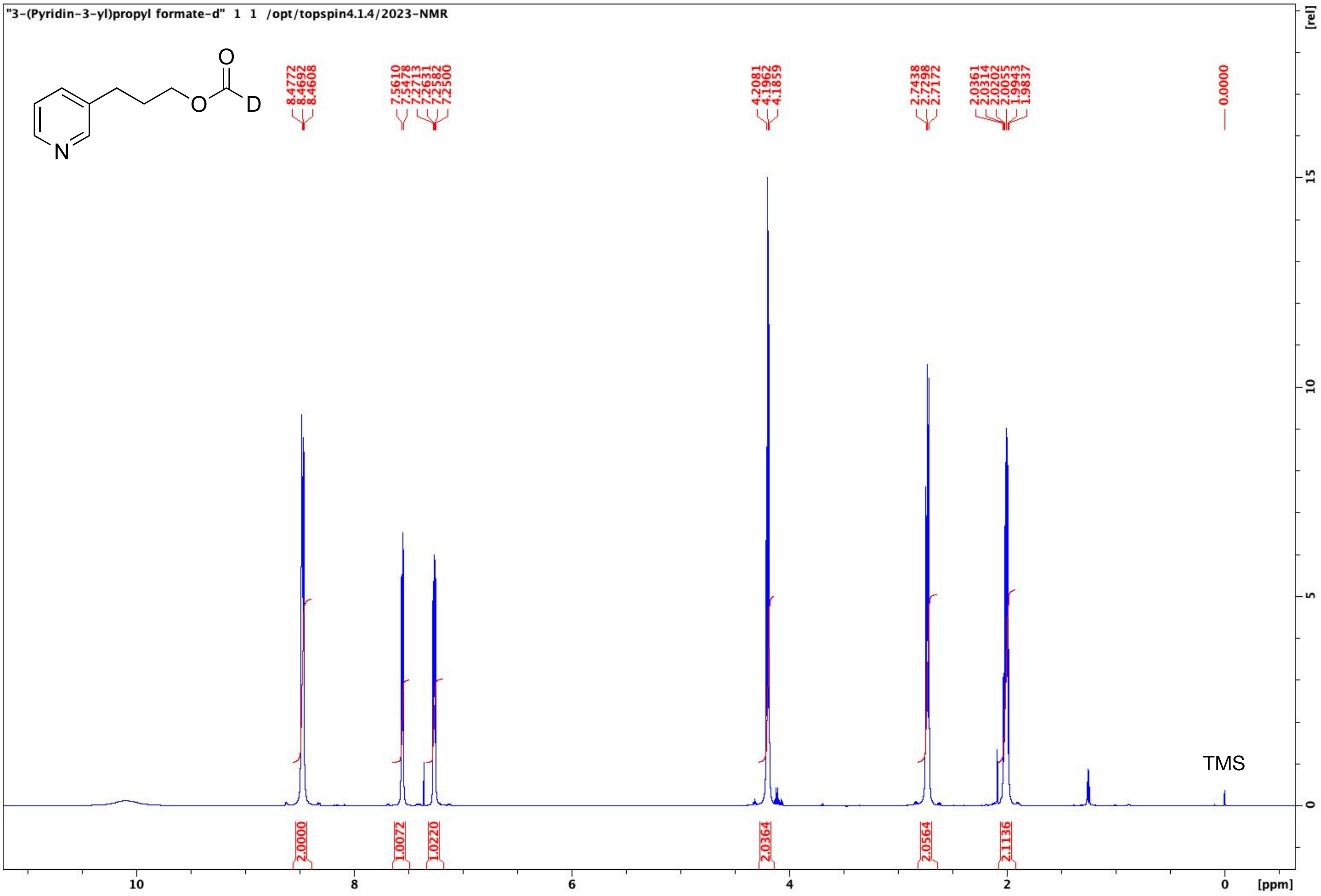

^{13}C NMR ($CDCl_3$ with 0.03 v/v% TMS, 150 MHz)

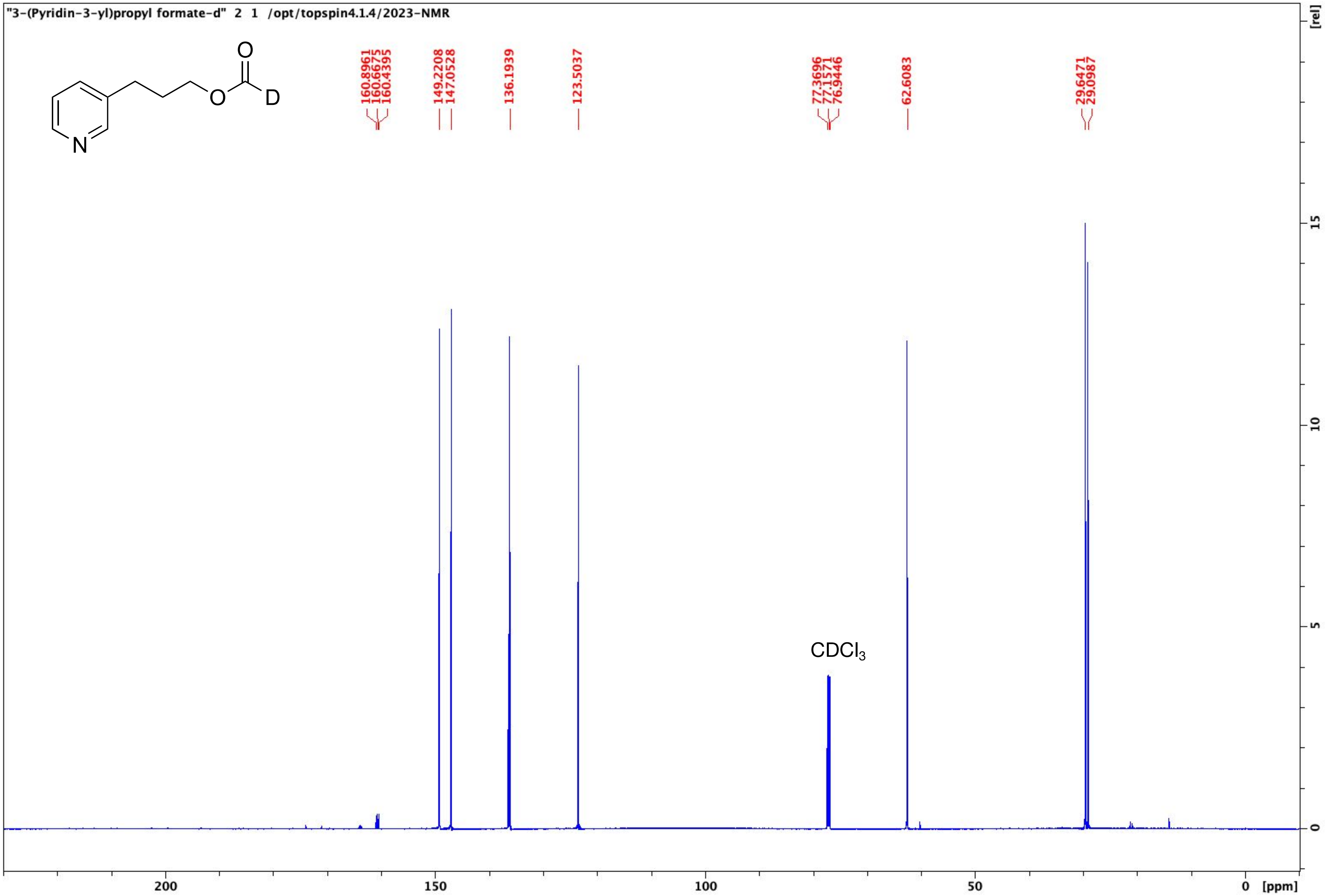

^1H NMR ($CDCl_3$ with 0.03 v/v% TMS, 600 MHz)

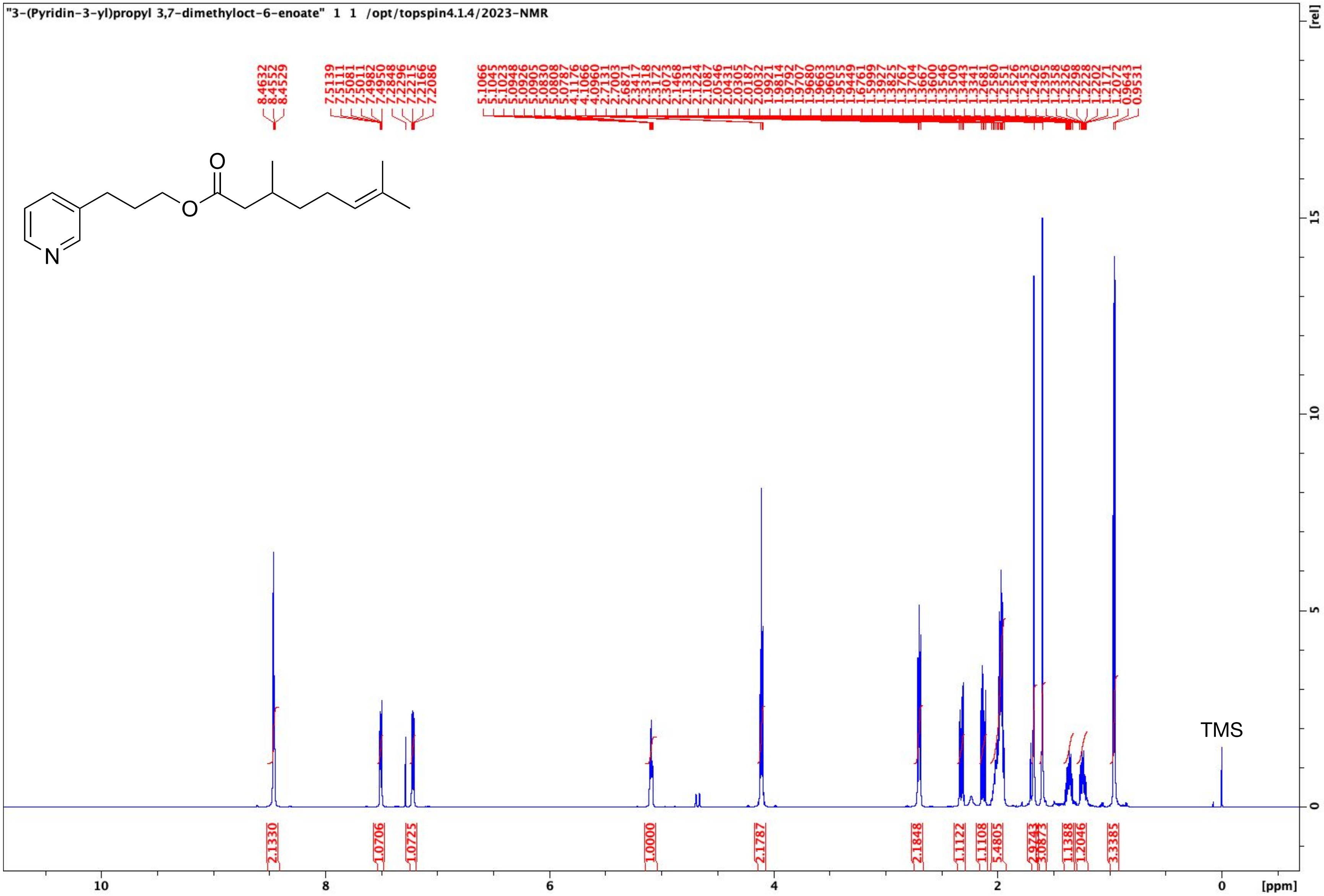

^{13}C NMR ($CDCl_3$ with 0.03 v/v% TMS, 150 MHz)

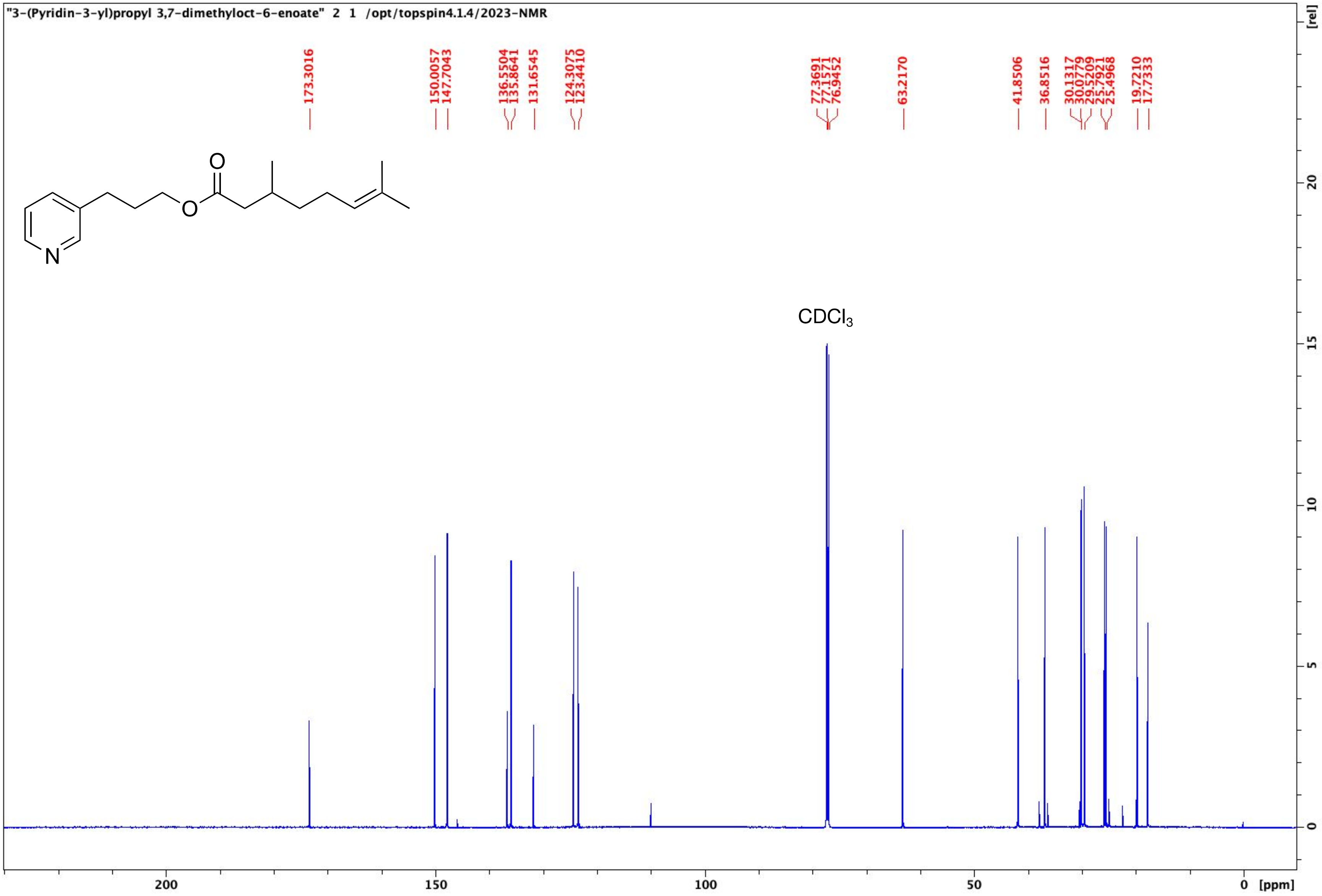

^1H NMR ($CDCl_3$ with 0.03 v/v% TMS, 600 MHz)

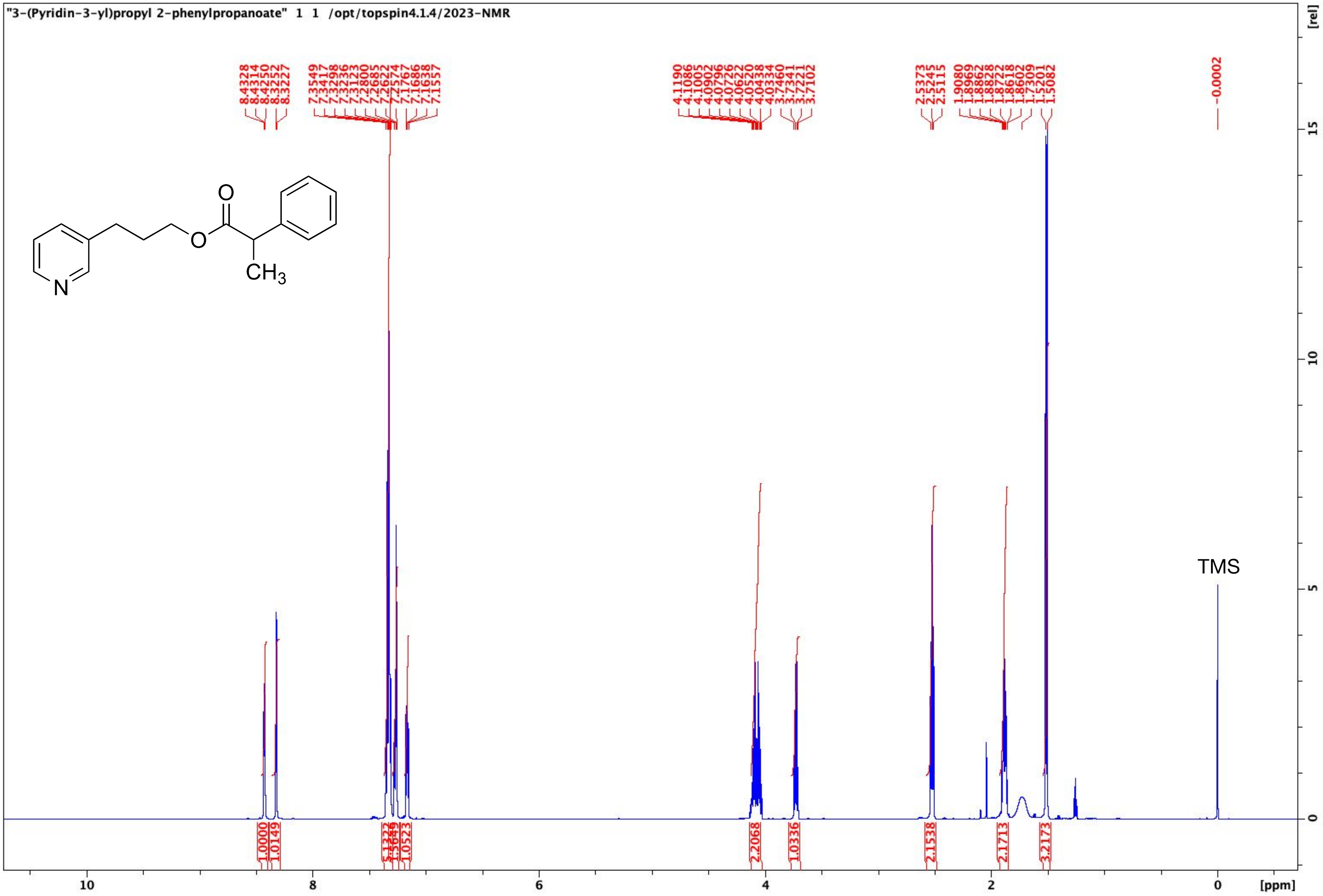

^{13}C NMR ($CDCl_3$ with 0.03 v/v% TMS, 150 MHz)

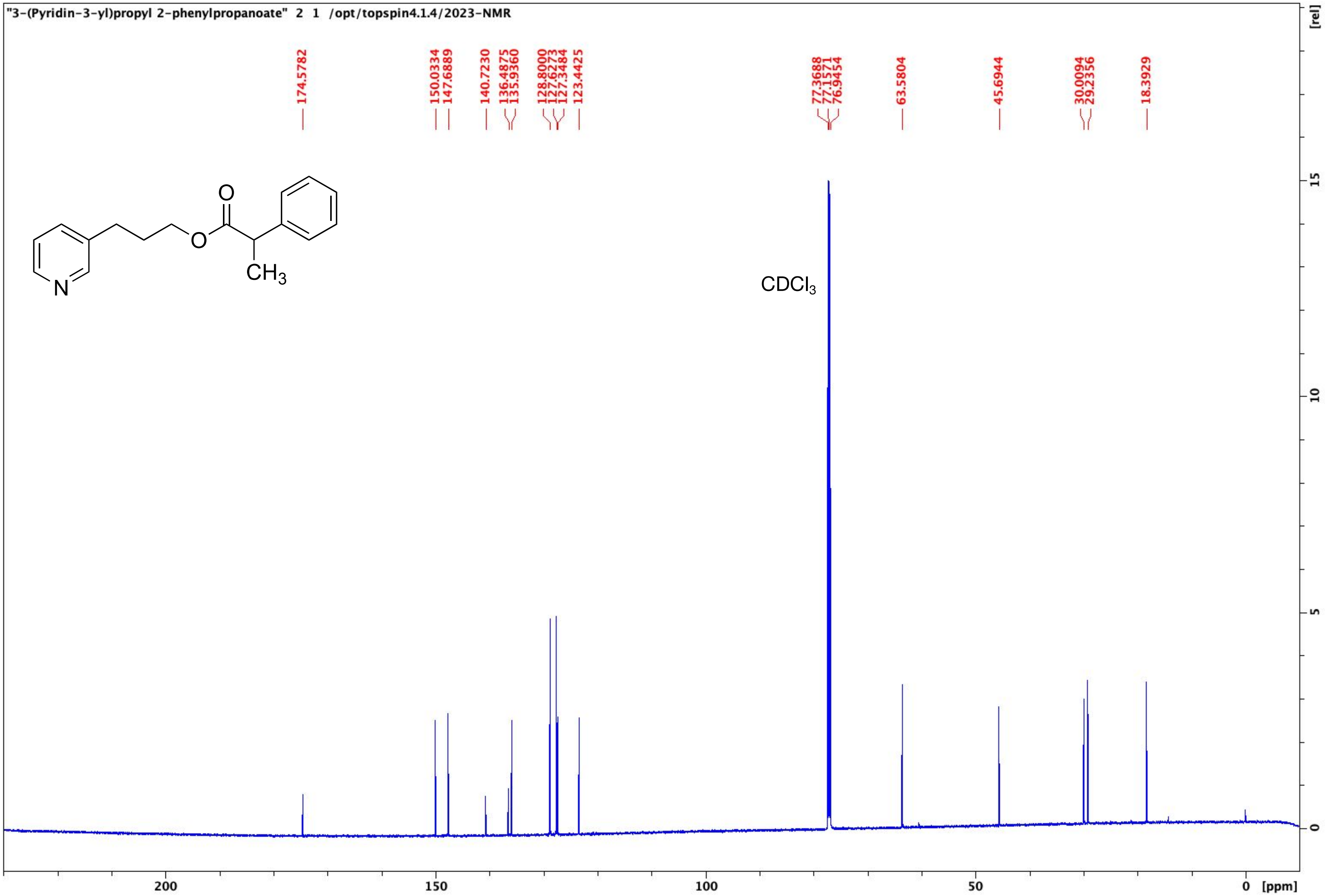

^{1}H NMR ($CDCl_3$ with 0.03 v/v% TMS, 600 MHz)

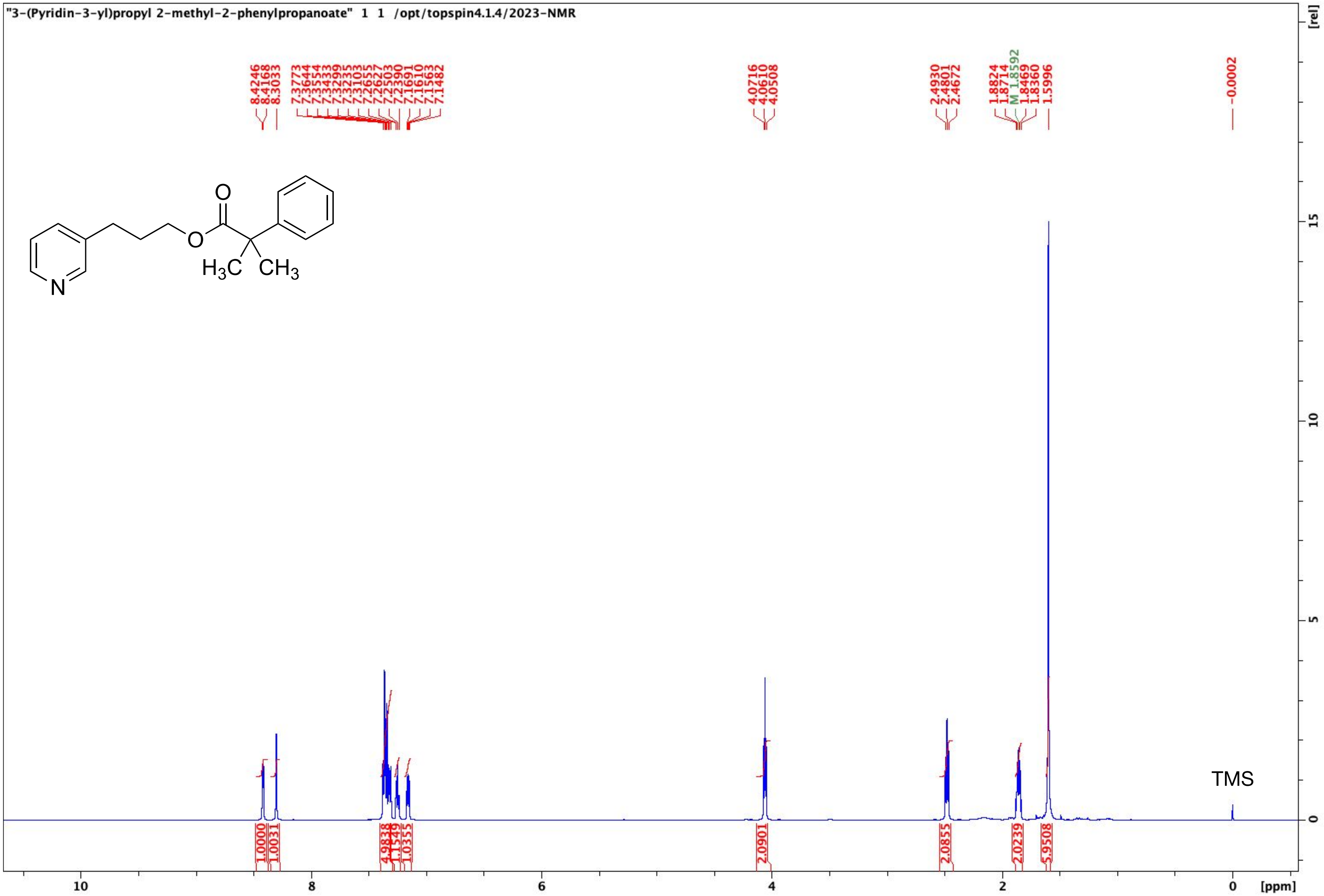

^{13}C NMR ($CDCl_3$ with 0.03 v/v% TMS, 150 MHz)

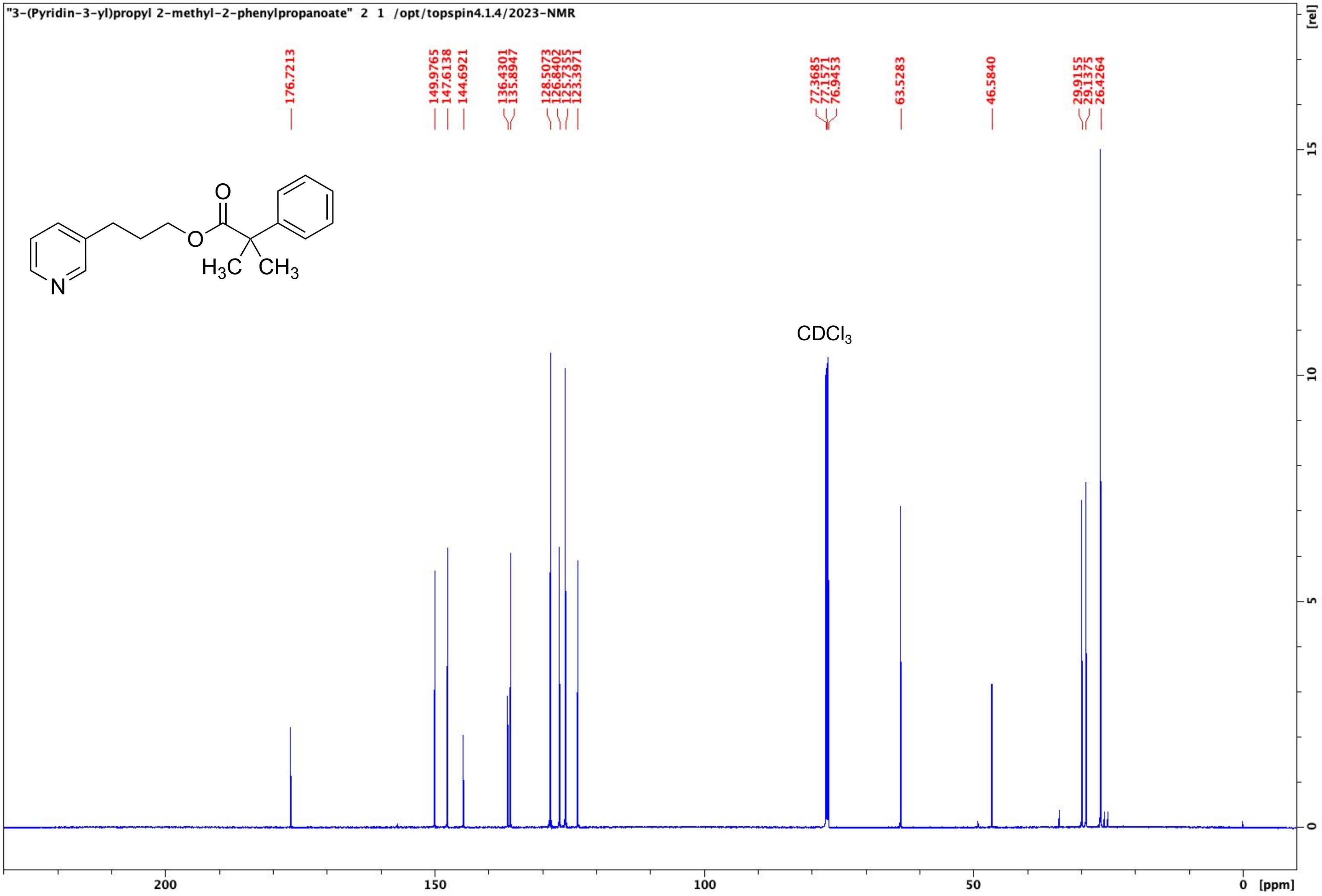

^1H NMR ($CDCl_3$ with 0.03 v/v% TMS, 600 MHz)

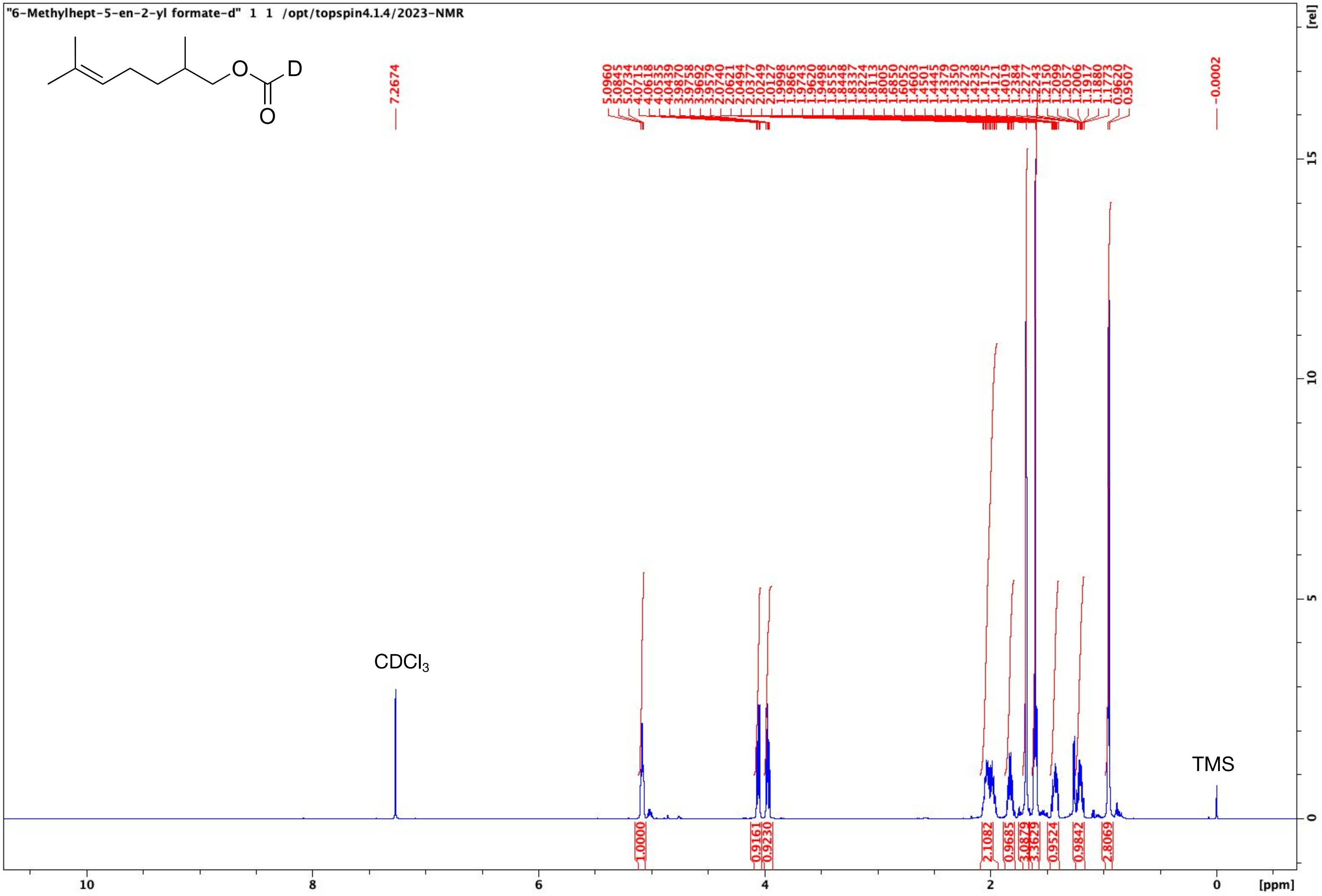

^{13}C NMR ($CDCl_3$ with 0.03 v/v% TMS, 150 MHz)

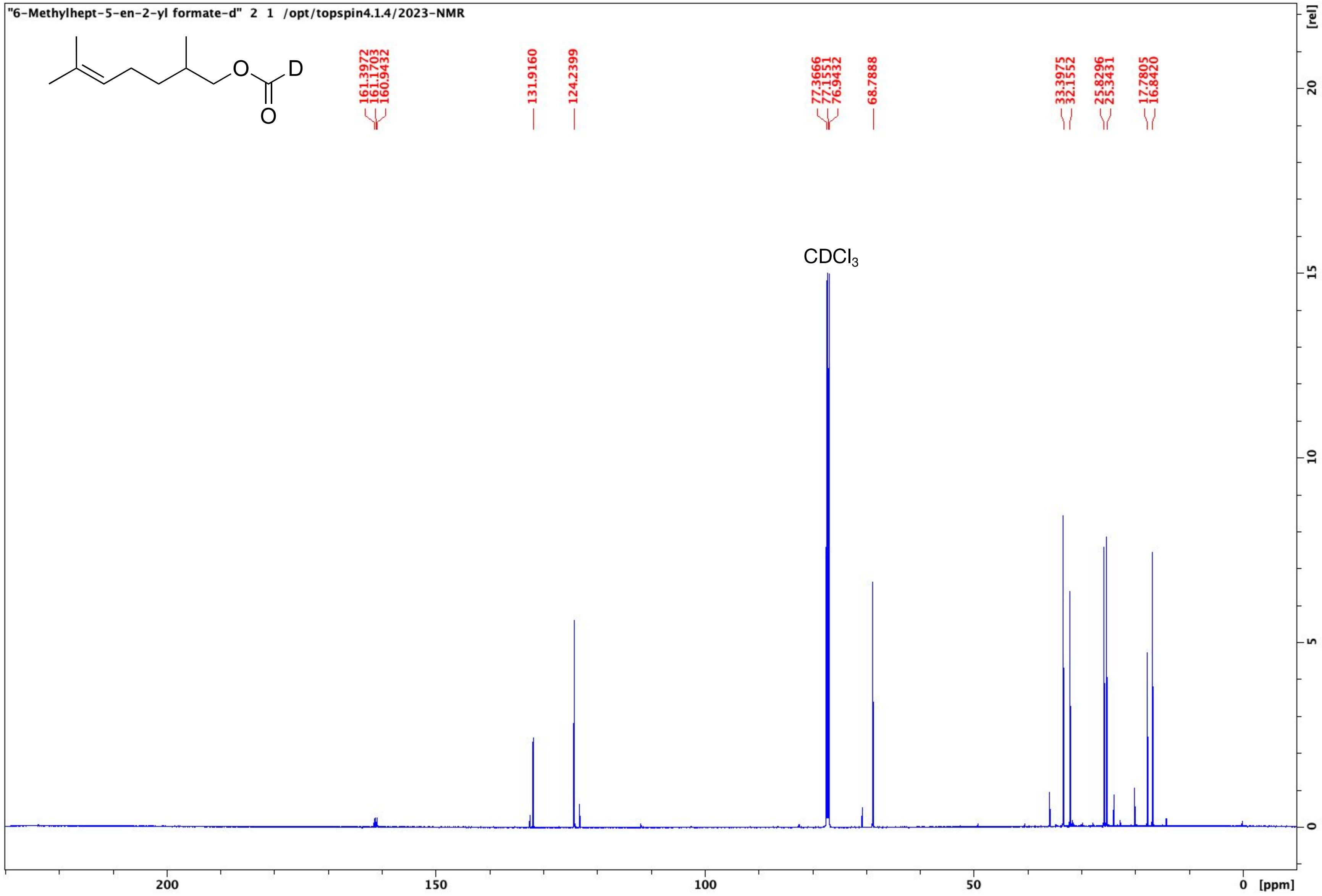

^{1}H NMR ($CDCl_3$ with 0.03 v/v% TMS, 600 MHz)

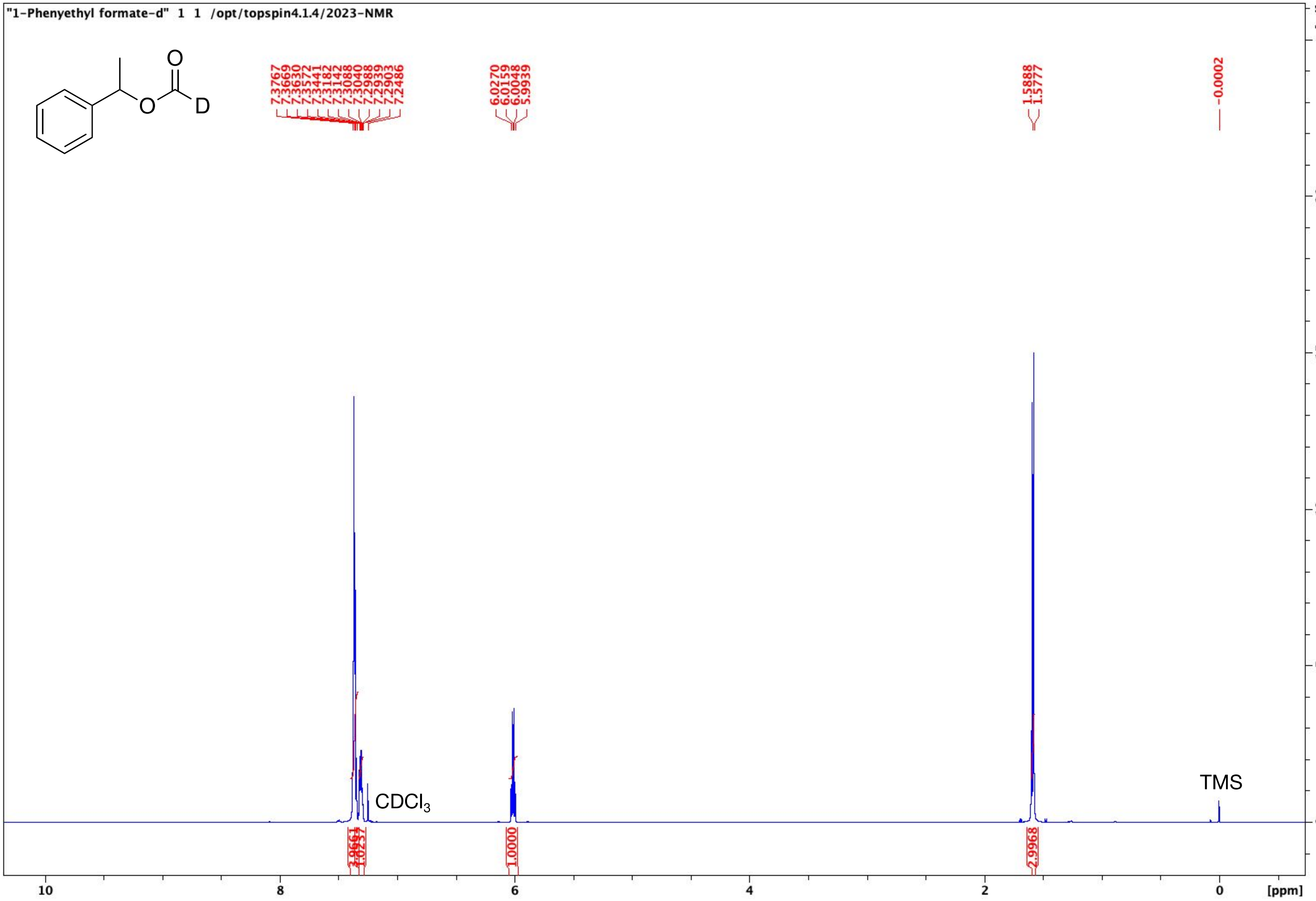

^{13}C NMR ($CDCl_3$ with 0.03 v/v% TMS, 150 MHz)

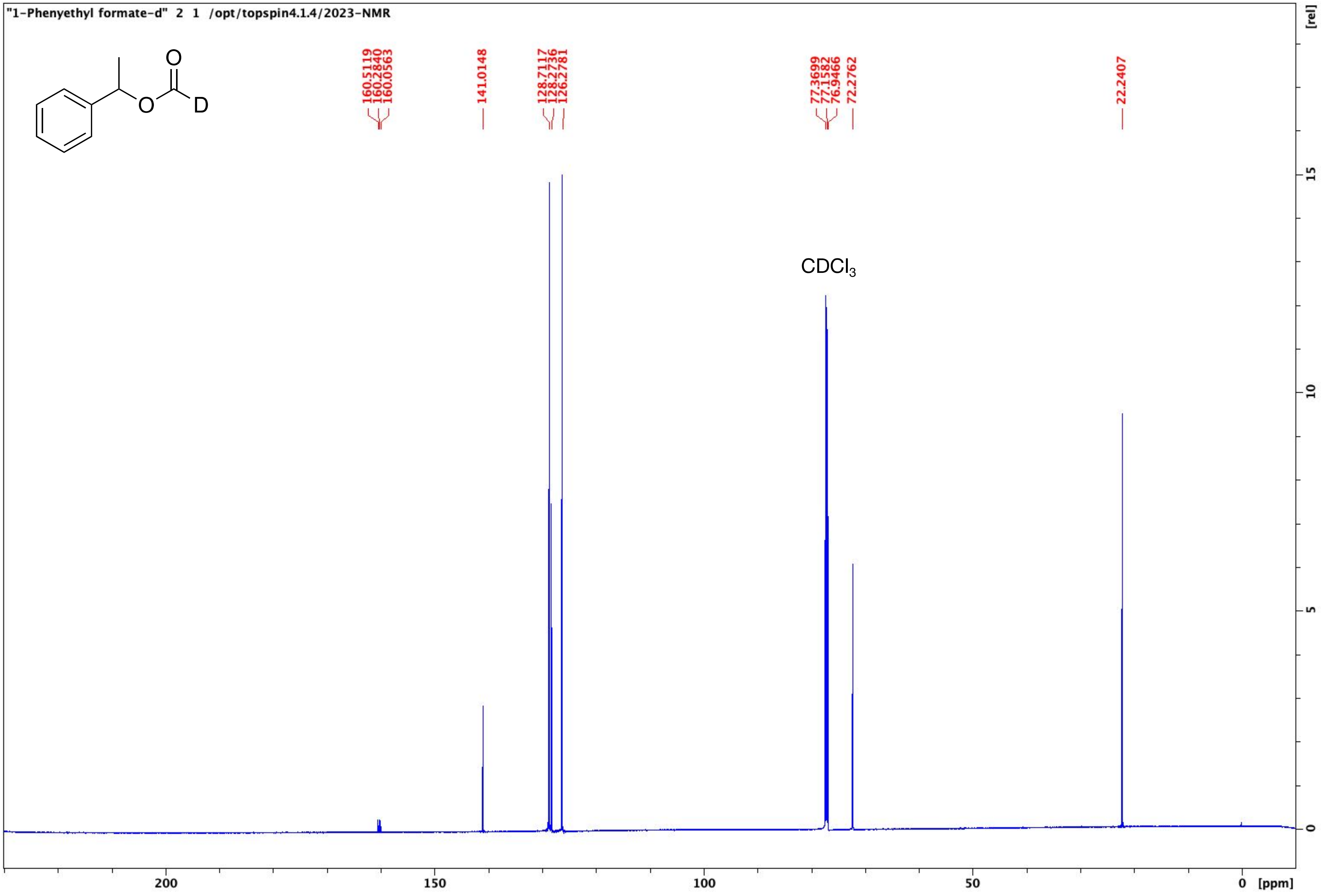

^{1}H NMR ($CDCl_3$ with 0.03 v/v% TMS, 600 MHz)

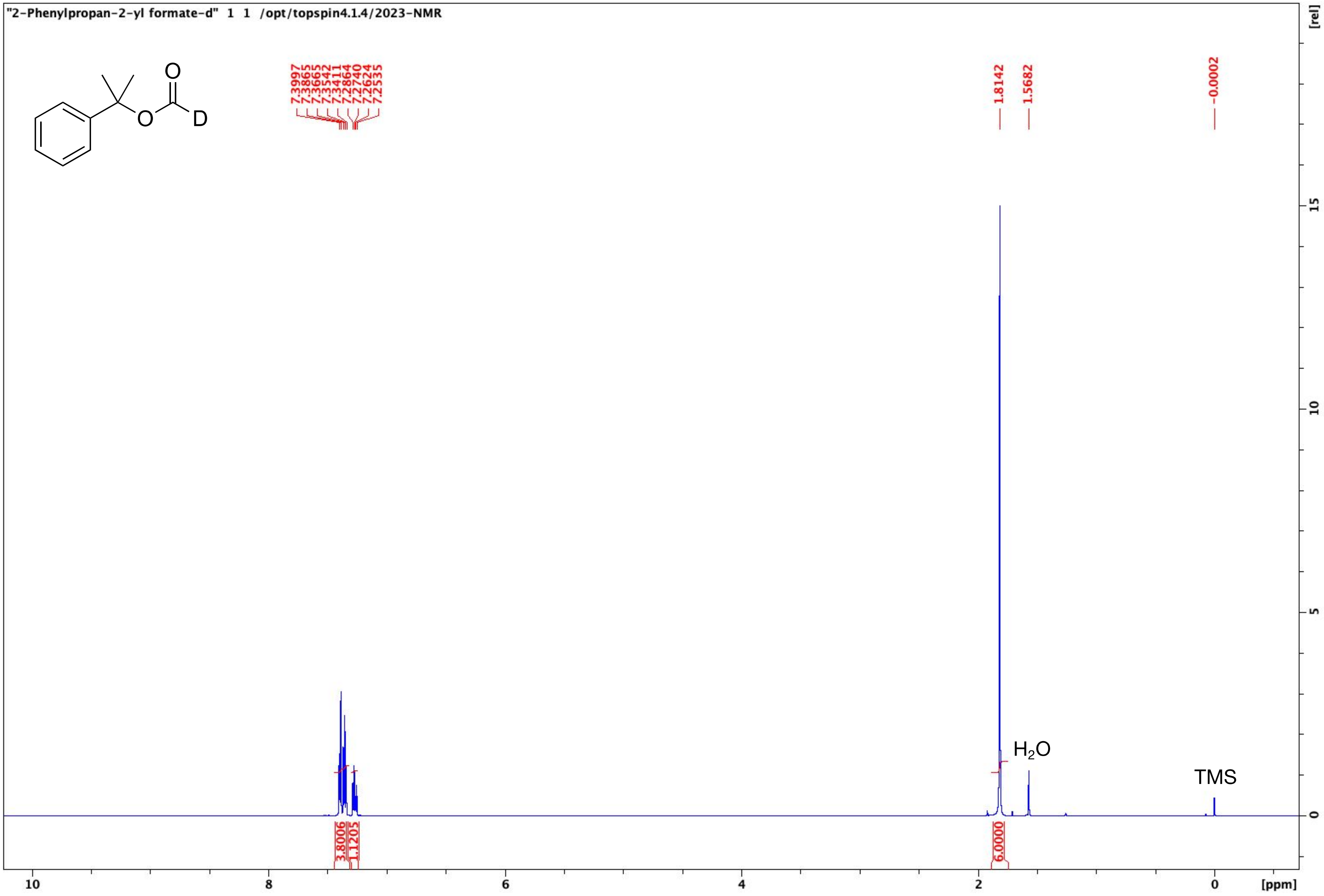

^{13}C NMR ($CDCl_3$ with 0.03 v/v% TMS, 150 MHz)

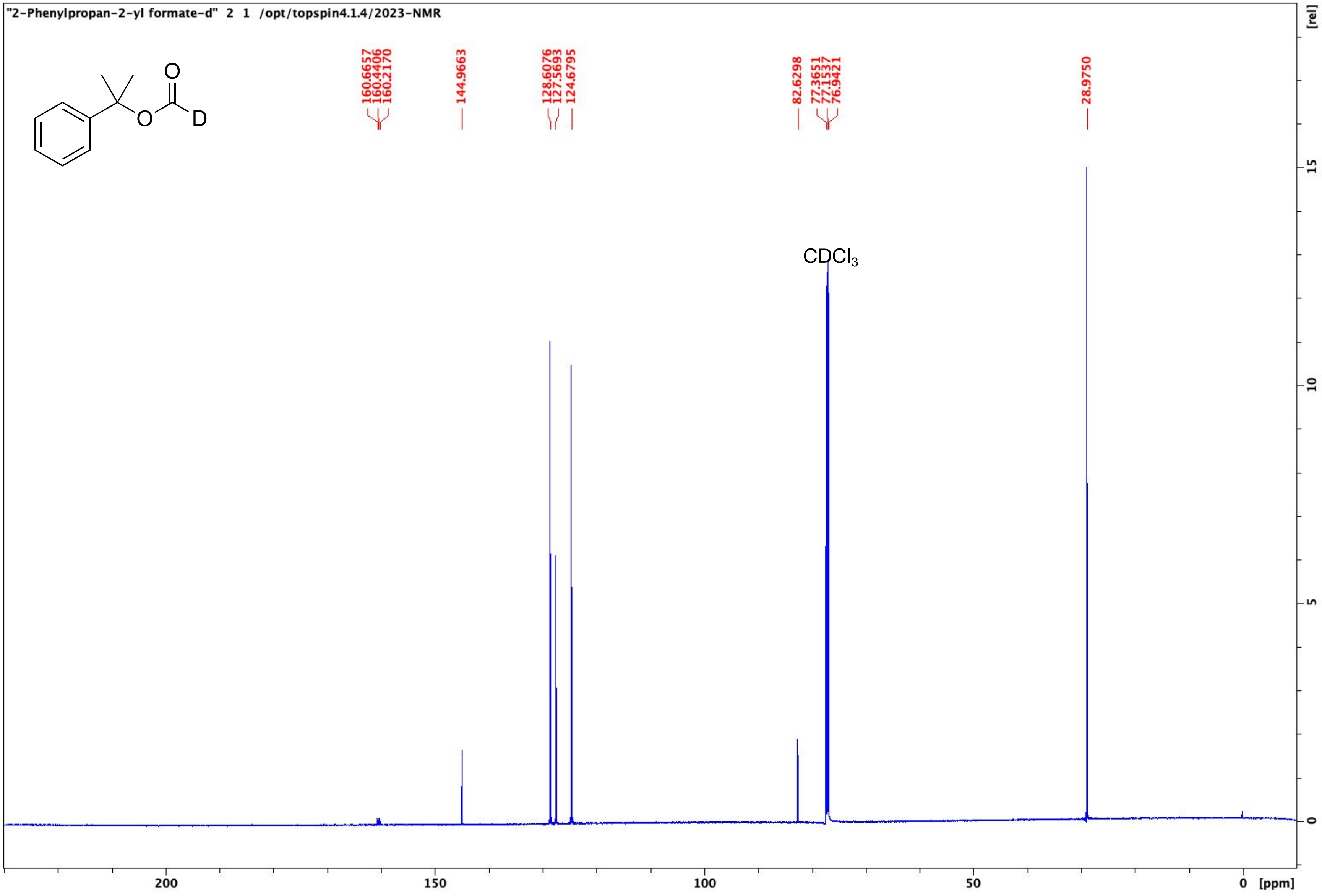

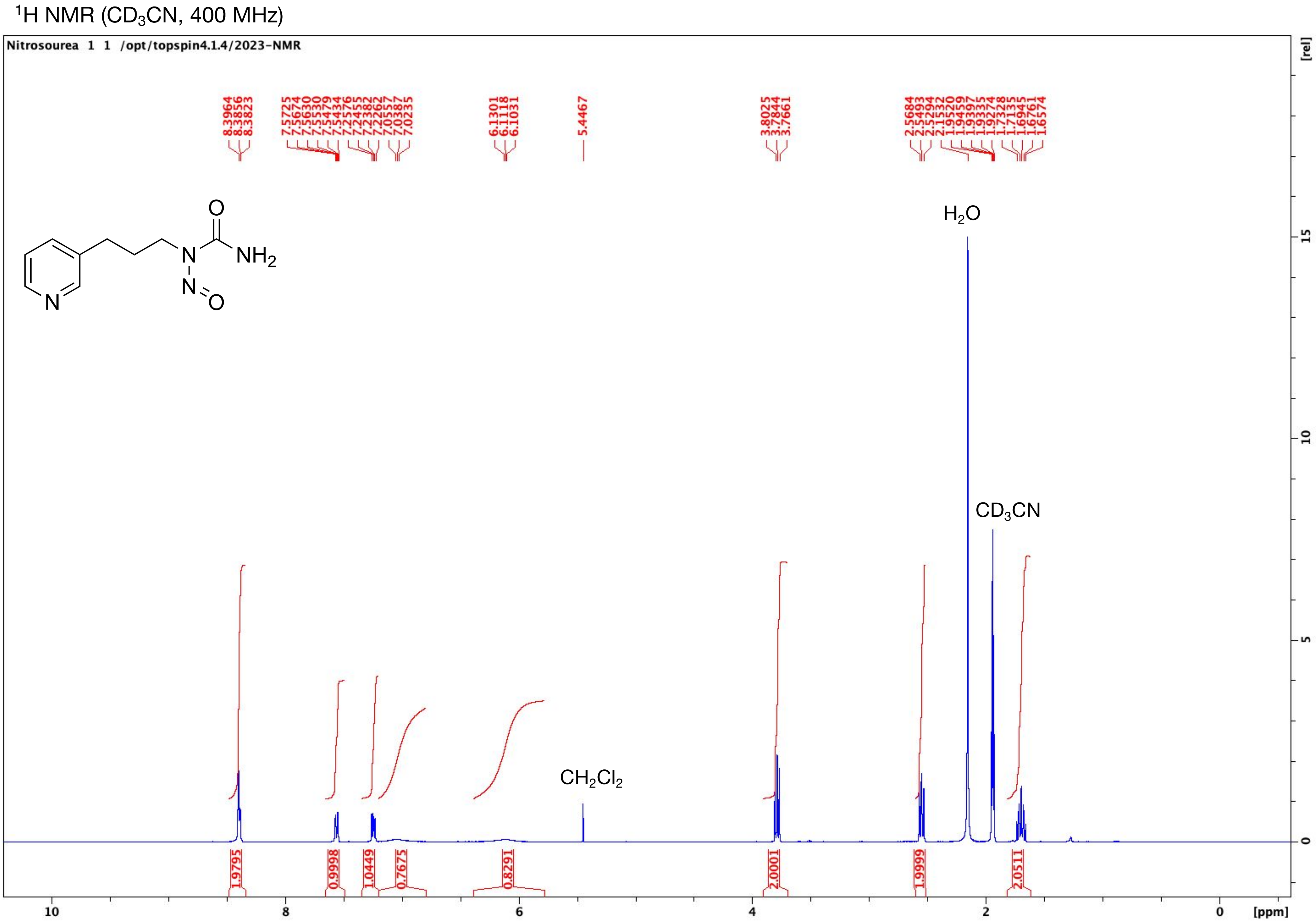

^{1}H NMR (CD$_3$CN, 400 MHz)
Nitrosourea 1 1 /opt/topspin4.1.4/2023-NMR
8.3964
8.3856
8.3823
7.5725
7.5674
7.5630
7.5530
7.5479
7.5434
7.2576
7.2455
7.2382
7.2262
7.0557
7.0387
7.0235
6.1301
6.1118
6.1031
5.4467
3.8025
3.7844
3.7661
2.5684
2.5493
2.5294
2.1532
1.9520
1.9459
1.9397
1.9335
1.9274
1.7328
1.7135
1.6945
1.6761
1.6574
H$_2$O
CD$_3$CN
CH$_2$Cl$_2$
NH$_2$
1.9795
0.9998
1.0449
0.7675
0.8291
2.0001
1.9999
2.0511
[rel]
[ppm]

References (for Supporting Information only)

1. Hill, R. K.; Abächerli, C.; Hagishita, S., Synthesis of (2*S*,4*S*)- and (2S,4*R*)-[5,5,5-$^2H_3$] Leucine from (*R*)-Pulegone. *Canad. J. Chem.* **1994,** *72* (1), 110-113.

2. Dess, D. B.; Martin, J. C., Readily Accessible 12-I-5 Oxidant for the Conversion of Primary and Secondary Alcohols to Aldehydes and Ketones. *J. Org. Chem.* **1983,** *48* (22), 4155-4156.

3. Kawaguchi, Y.; Yasuda, S.; Kaneko, A.; Oura, Y.; Mukai, C., Rhodium(I)-Catalyzed Cycloisomerization of Benzylallene-Alkynes through C-H Activation. *Angew. Chem. Int. Ed.* **2014,** *53* (29), 7608-7612.

4. Vogt, M.; Ceylan, S.; Kirschning, A., Stereocontrolled Palladium-Catalysed Umpolung Allylation of Aldehydes with Allyl Acetates. *Tetrahedron* **2010,** *66* (33), 6450-6456.

5. Yoshimoto, F. K.; Guengerich, F. P., Mechanism of the Third Oxidative Step in the Conversion of Androgens to Estrogens by Cytochrome P450 19A1 Steroid Aromatase. *J. Am. Chem. Soc.* **2014,** *136* (42), 15016-15025.

6. Hoover, D. M.; Lubkowski, J., DNAWorks: An Automated Method for Designing Oligonucleotides for PCR-Based Gene Synthesis. *Nucleic Acids Res.* **2002,** *30* (10), e43.

7. Wu, Z. L.; Bartleson, C. J.; Ham, A. J.; Guengerich, F. P., Heterologous Expression, Purification, and Properties of Human Cytochrome P450 27C1. *Arch. Biochem. Biophys.* **2006,** *445* (1), 138-46.

8. Sandhu, P.; Baba, T.; Guengerich, F. P., Expression of Modified Cytochrome P450 2C10 (2C9) in *Escherichia coli*, Purification, and Reconstitution of Catalytic Activity. *Arch. Biochem. Biophys.* **1993,** *306* (2), 443-50.

9. Brown, R. E.; Jarvis, K. L.; Hyland, K. J., Protein Measurement Using Bicinchoninic Acid: Elimination of Interfering Substances. *Anal. Biochem.* **1989,** *180*, 136-139.

10. Guengerich, F. P.; McCarty, K. D.; Tateishi, Y.; Liu, L., Steroid 17α-hydroxylase/17,20-Lyase (Cytochrome P450 17A1). *Methods Enzymol.* **2023,** *689*, 39-63.

11. Hanna, I. H.; Teiber, J. F.; Kokones, K. L.; Hollenberg, P. F., Role of the Alanine at Position 363 of Cytochrome P450 2B2 in Influencing the NADPH- and Hydroperoxide-Supported Activities. *Arch. Biochem. Biophys.* **1998,** *350*, 324-332.

12. Guengerich, F. P., Analysis and Characterization of Enzymes and Nucleic Acids Relevant to Toxicology. In *Hayes' Principles and Methods of Toxicology*, 6th ed.; Hayes, A. W.; Kruger, C. L., Eds. CRC Press-Taylor & Francis Boca Raton, FL, 2014; pp 1905-1964.

13. Burleigh, B. D., Jr.; Foust, G. P.; Williams, C. H., Jr., A Method for Titrating Oxygen-Sensitive Organic Redox Systems with Reducing Agents in Solution. *Anal. Biochem.* **1969,** *27*, 536-544.

14. Guengerich, F. P.; Johnson, W. W., Kinetics of Ferric Cytochrome P450 Reduction by NADPH-Cytochrome P450 Reductase: Rapid Reduction in the Absence of Substrate and Variations among Cytochrome P450 Systems. *Biochemistry* **1997,** *36* (48), 14741-50.

15. Vorbeck, M. L.; Mattick, L. R.; Lee, F. A.; Pederson, C. S., Preparation of Methyl Esters of Fatty Acids for Gas-Liquid Chromatography. Quantitative Comparison of Methylation Techniques. *Anal. Chem.* **1961,** *33* (11), 1512-1514.

16. Schlenk, H.; Gellerman, J. L., Esterification of Fatty Acids with Diazomethane on a Small Scale. *Anal. Chem.* **1960,** *32* (11), 1412-1414.

17. Knapp, D. R., *Handbook of Analytical Derivatization Reactions*. John Wiley & Sons, New York, 1979, pp. 156-157.

18. McCarty, K. D.; Tateishi, Y.; Hargrove, T. Y.; Lepesheva, G. I.; Guengerich, F. P., Oxygen-18 Labeling Reveals a Mixed Fe–O Mechanism in the Last Step of Cytochrome P450 51 Sterol 14α-Demethylation. *Angew. Chem. Int. Ed.* **2024,** e202317711

19. Glasoe, P. K.; Long, F. A., Use of Glass Electrodes to Measure Acidities in Deuterium Oxide. *J. Phys. Chem.* **1960,** *64*, 188-190.

20. Ecott, E. E.; White, M. A.; Johnson, E. F.; Stout, C. D.; Halpert, J. R., Structure of Mammaliam Cytochrome P450 2B4 Complexed with 4-(4-Chlorophenyl)imidazole at 1.9-Å Resolution: Insight into the Range of P450 Conformations and the Coordination of Redox Partner Binding. *J. Biol. Chem.* **2004,** *279 (26)*, 27294-27301.

21. Omura, T.; Sato, R., The Carbon Monoxide-Binding Pigment of Liver Microsomes. I. Evidence for Its Hemoprotein Nature. *J. Biol. Chem.* **1964,** *239*, 2370-2378.
